# Transcriptome profiling in fast versus slow-growing rainbow trout across seasonal gradients

**DOI:** 10.1186/s12864-016-2363-5

**Published:** 2016-01-15

**Authors:** Roy G. Danzmann, Andrea L. Kocmarek, Joseph D. Norman, Caird E. Rexroad, Yniv Palti

**Affiliations:** Department of Integrative Biology, University of Guelph, 50 Stone Road East, Guelph, ON N1G 2W1 Canada; National Center for Cool and Cold Water Aquaculture, ARS-USDA, 11861 Leetown Road, Kearneysville, WV 25430 USA

**Keywords:** RNAseq, White muscle, Sarcomere assembly, Exercise physiology, Metabolic profile

## Abstract

**Background:**

Circannual rhythms in vertebrates can influence a wide variety of physiological processes. Some notable examples include annual reproductive cycles and for poikilotherms, seasonal changes modulating growth. Increasing water temperature elevates growth rates in fishes, but increases in photoperiod regime can have similar influences even at constant temperature. Therefore, in order to understand the dynamics of growth in fish it is important to consider the background influence of photoperiod regime on gene expression differences. This study examined the influence of a declining photoperiod regime (winter solstice) compared to an increasing photoperiod regime (spring equinox) on white muscle transcriptome profiles in fast and slow-growing rainbow trout from a commercial aquaculture strain.

**Results:**

Slow-growing fish could be characterized as possessing transcriptome profiles that conform in many respects to an endurance training regime in humans. They have elevated mitochondrial and cytosolic creatine kinase expression levels and appear to suppress mTOR-signaling as evidenced by elevated TSC2 expression, and they also have elevated p53 levels. Large fish display a physiological repertoire that may be consistent with strength/resistance physiology having elevated cytoskeletal gene component expression and glycogen metabolism cycling along with higher PI3K levels. In many respects small vs. large fish match eccentric vs. concentric muscle expression patterns, respectively. Lipid metabolic genes are also more elevated in larger fish, the most notable being the G0S2 switch gene. M and Z-line sarcomere remodelling appears to be more prevalent in large fish. Twenty-three out of 26 gene families with previously reported significant SNP-based growth differences were detected as having significant expression differences.

**Conclusions:**

Larger fish display a broader array of genes showing higher expression, and their profiles are more similar to those observed in December lot fish (i.e., an accelerated growth period). Conversely, small fish display gene profiles more similar to seasonal growth decline phases (i.e., September lot fish). Overall, seasonal timing was coupled to greater differences in gene expression compared to differences associated with fish size.

**Electronic supplementary material:**

The online version of this article (doi:10.1186/s12864-016-2363-5) contains supplementary material, which is available to authorized users.

## Background

Growth in fishes is a complex physiological trait involving many interacting environmental and genetic factors. Environmental factors act to both enhance and constrain the expression of underlying genes that may influence growth such that different environments may modulate growth phenotypes to the extent that seemingly identical genotypes may often yield opposite or opposing phenotypic outcomes. Indeed such genotype x environment interactions can be common place in quantitative genetic studies making predictions of growth performance solely on genotypic indices difficult, or potentially flawed. Identifying genes that may be key indicators of potential growth performance in fishes is even more complicated than in other vertebrate classes, given that fish muscle growth is influenced by genes that both regulate muscle cell recruitment and myogenic hyperplasia or cellular proliferation, as well as genes that regulate the growth and fusion of individual myotubes leading to myofibrillar formation and hypertrophy [1]. Hypertrophy may be regarded as a growth stage in muscle development leading to an increase in muscle biomass. Species within other vertebrate classes experience muscle growth primarily via hypertrophy after a period of early juvenile myogenesis of precursor satellite cells. Environmental factors, however, may regulate both hyperplasic and hypertrophic cycles in fishes and understanding how the environment affects both of these processes is critical to understanding fish growth.

Fish growth can be cyclic in nature especially in temperate species where seasonal shifts in both photoperiod and temperature can condition fish into faster growth phases (spring and summer growth profiles) and slower growth periods accompanied by declining water temperatures and photoperiod regimes (fall and winter periods). Additionally in certain groups of fishes such as the salmonids, it has also been demonstrated that individual fish can have variable growth cycles that differ significantly on a daily basis [2, 3], and may also exhibit rhythms dependent upon full and new moon cycles [4, 5]. Over-riding these cycles may be physiological ‘set points’ that can initiate developmental transitions from one life-history stage to another. For example, it is known that previous growth trajectories and lipid metabolic stores may trigger the onset of sexual maturation and smoltification events in species such as Atlantic salmon (*Salmo salar*) and Chinook salmon (*Oncorhynchus tshawytscha*) [6–8]. As such, size thresholds reached by a fish during their early juvenile growth phases can initiate profound physiological changes among individuals at varying ages even within a single family.

Many of the direct environmental inputs that can alter the growth expression of a fish are nutritional in nature and therefore are difficult, if not impossible to control unless studies are undertaken in a laboratory setting. Even within a controlled laboratory or hatchery setting, where experimental lots of fish are fed identical diets, it is impossible to correct for intraspecific social interactions that establish social dominance hierarchies that may in turn influence feeding rates and growth unless fish are held in isolation. This in turn has confounding influences as well, as some species (e.g., Arctic charr, (*Salvelinus alpinus*)) are known to inhibit feeding responses unless they are reared above certain stocking densities [9]. While it is acknowledged that no method of husbandry can produce exactly perfect rearing conditions in which to measure growth performance, insights into the genes that may be important in altering growth rates can be obtained by rearing fish under similar conditions related to biomass densities and feeding rates in controlled hatchery conditions, while altering only one or two environmental conditions to assess their influence.

**Fig. 1:**
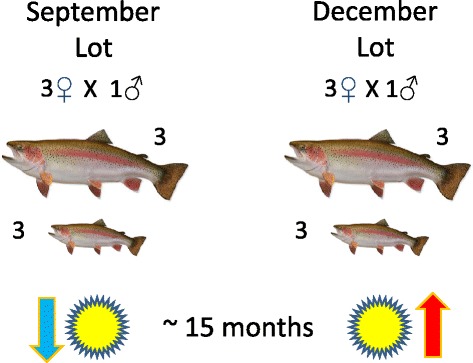
Experimental design depicting the selection of size-matched differences of a large and small fish selected from 3 different paternal half-sib families in each of two seasonal spawning lots (September and December). September fish were sampled during a declining growth phase (winter solstice) while December lot fish were sampled during an increasing growth phase (spring equinox) when they were approximately 15 months of age.

We examined the influence of photoperiod as induced by seasonality on the gene expression profiles of white muscle tissue of large and small size-selected rainbow trout (*Oncorhynchus mykiss*). Two lots of fish derived from a commercial strain were made in September and December of 2008, but were tested when fish in both lots were approximately 15 months of age. The September lot was examined in December when the photoperiod was declining towards winter solstice while the December lot was examined in late February during a period of increasing photoperiod towards spring equinox (Fig. 1). Seasonality was found to have a profound influence on white muscle gene expression profiles in these fish. Despite the strong influence of photoperiod regime on gene expression differences, many salient differences were also evident between large and small fish that were selected for the experiment, which appeared to be consistent patterns of gene expression across growing seasons. We regard the genes showing strong up-regulation of expression in either fast-growing or slow-growing rainbow trout across seasons as potential signature genes for growth differences in salmonid fishes and these genes may serve as a model for other temperate fish species having similar nutritional requirements to salmonid fishes.

## Results and discussion

**Fig. 2:**
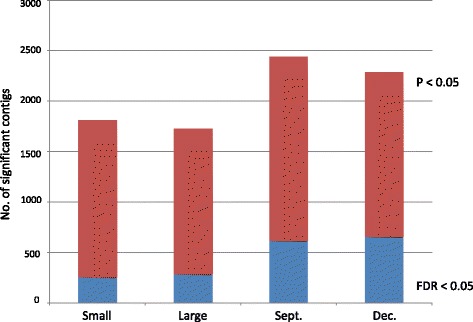
Number of contigs (Y-axis) with differential gene expression at the nominal P ≤ 0.05 and FDR 0.05 level in large and small fish, and across seasonal groupings (December vs. September lots) out of 31,600 possible contigs assessed (edge R analysis)

**Fig. 3:**
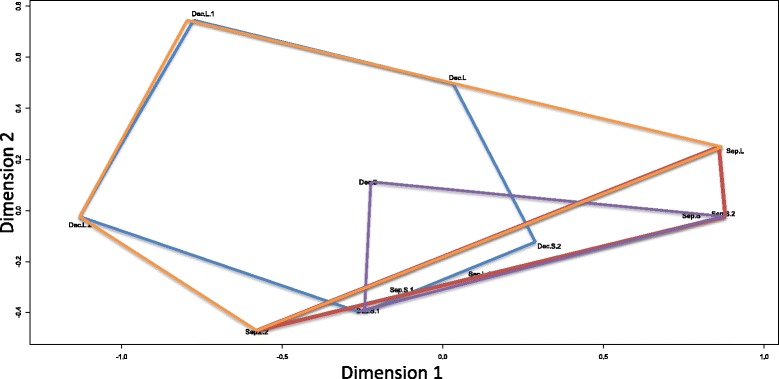
Multidimensional Scaling Plot (edgeR analysis) of gene expression profiles observed within December lot fish (*blue polygon*), September lot fish (*red polygon*), large fish (*orange polygon*), and small fish (*purple polygon*)

On average each read aligned to 4.11 possible contigs using the NextGENe alignment parameters. Following edgeR filtering 31,600 contigs were detected as contributing to the expression profiles among the 12 fish tested for size and seasonal differences. Box plots for the normalized RPKM values of these contigs among the 12 fish are shown in Additional file 1. Seasonal sampling times had a far greater influence on differential transcriptome abundance patterns in the fish than did size differences between the fish. The reads from more than twice as many contigs were observed to be significantly more abundant (FDR < 0.05) in either the September lot (614) or December lot (653) compared to small (256) and large (282) fish (Fig. 2). MDS profiles also indicate that expression profiles are more similar within either September or December lot fish rather than clustering based upon the size categories (Fig. 3).

### Size differences

**Fig. 4:**
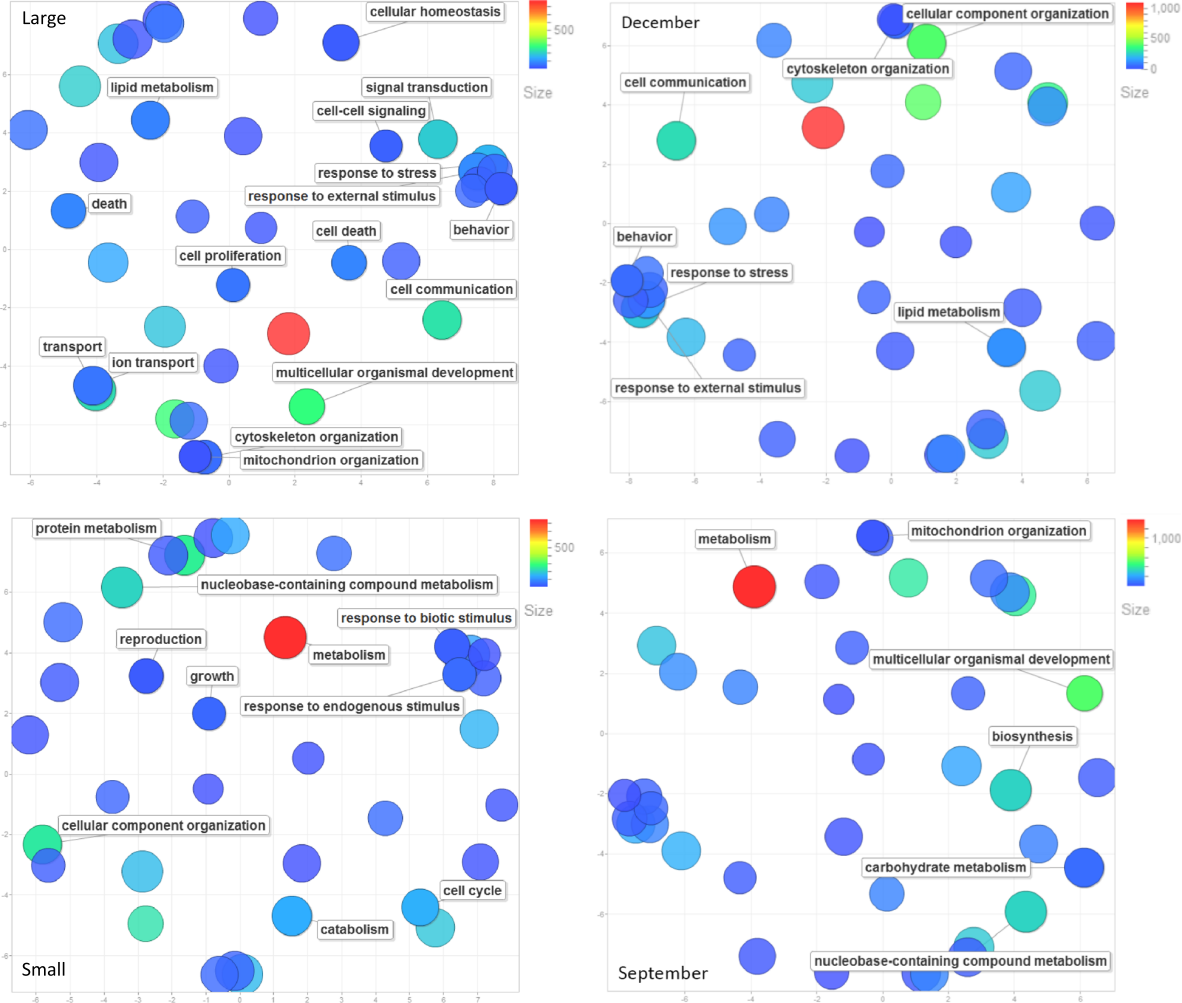
Results from the REVIGO analysis analyzing significant differences in FDR corrected gene counts for biological process GOSlim categories. Large and Small fish differences are depicted in the two left-had panels, while differences between December vs. September fish are shown in the right-hand panels. Gene Ontology categories with significantly different higher gene counts between large vs. small fish and between December vs. September fish are indicated

Genes differentially expressed between large and small fish are shown in Additional files 2 and 3, respectively. Results from the compilation of GO terms into GO Slim categories and their REVIGO estimations indicate that 16 categories possessed higher gene counts in large fish, while 9 categories were more abundant in small fish (Fig. 4). The greater variation in ontology classes upregulated in large fish is also supported by the Panther-AMIGO 2 GSEA analysis (Additional file 4). In total, 99 Biological Process GO terms were associated with over-represented gene classes in the large fish, while 8 GO terms were related to genes having higher counts in the small fish (Additional file 4). Terms related to ‘carbohydrate’, ‘lipid’, ‘stress response’, ‘blood properties’, and ‘response to wounding’ were present in the large fish, while terms related to ‘muscle fiber organization’ were more abundant in small fish, Terms such as  ‘nucleotide/nucleoside processing’ were common to both groups. BROAD Institute GSEA configurations obtained with BP, BIOCARTA, KEGG, and REACTOME pathway analysis yielded essentially the same results as those obtained from the Panther-based analysis in that 99 terms were more highly represented in large fish (FDR q-value < = 0.05), while 42 terms were more highly represented in small fish (Additional files 5 and 6, respectively). This latter GSEA emphasizes the expression of apoptotic pathway genes in larger fish. Differences in the combined analysis relate to ’transport’ components. The GSEA indicates genes within the ‘transport’ GO category are more highly expressed in small fish, whereas the GO Slim CateGOrizer compilations indicate that the terms ‘transport’ and ‘ion transport’ are more highly delineated in large fish.

To assess which gene classes could be considered to contain genes that provide a signature of enhanced growth capabilities we referenced the GSEA Broad Institute analyses output as this gives a listing of signature transcripts aligned to each over-represented pathway category. Within these groupings large fish possessed many transcripts with overlapping functions related to carbohydrate metabolism (i.e., Glycolysis, Gluconeogenesis, Pentose phosphate pathway, Fructose and mannose metabolism, Glucose metabolic process, etc.), lipid metabolism (i.e., Pentose phosphate pathway, PPAR signaling pathway, Metabolism of lipids and lipoproteins, Fatty acid triacylglycerol and ketone body metabolism, Lipid digestion mobilization and transport, etc.), and the stress response including Immune response. This latter category included genes in the p38 downstream pathways, immune system, innate immune system, complement cascade, classical complement pathway, cMyc and cMyb activated pathways, and IL4 pathways (Additional file 5). Pathway analysis also indicated that processes such as Cell death, Apoptosis, Programmed cell death, Regulation of programmed cell death, and pathways related to vascular processes such as Complement and coagulation cascades, and Lymph angiogenesis were more highly expressed in large fish.

Smaller fish were characterized by higher abundance of gene transcripts related to several Catabolic process pathways, as well as regulation of the nervous system, nucleic acids, and translation, several categories related to muscle development and protein metabolism, Cardiomyopathy, and transport categories including Protein transport (Additional file 6). Large and small fish shared genes belonging to Regulation of biological quality, System process, Organ development, System development, Anatomical structure development, Multicellular organism development, and REACTOME immune system suggesting that genes within these categories may not be the best predictors of growth differences between large and small fish. However, none of the 12 or 9 genes detected within large and small fish, respectively, and assigned to the REACTOME Immune system category were shared between the size classes.

Contigs from certain gene sets were highly expressed in both large and small fish. As an example, it was observed that although the repertoire of genes in the Glycolysis pathway was greater in larger fish, two gene groups, Glyceraldehyde phosphate −3-dehydrogenase (GAPDH), and Phosphofructokinase muscle (PFKM) were highly expressed in both large and small fish. These two gene classes were also shared in the cMyc active pathway between the two size classes. Future research may still establish the utility of utilizing subsets of genes within gene set pathways shared by large and small fish in defining growth patterns between the size classes.

The single most abundant class of transcripts detected in the large fish were the G0/G1 switch 2 genes (G0S2). These genes are involved in lipid metabolism in vertebrates via negative regulation of adipose triglyceride lipase enzyme (ATGL) levels, and may also play a role in apoptosis and inflammation [10]. The transcripts from this gene were also discovered to be the most highly up-regulated gene in adipose tissue from a single large rainbow trout sampled from a commercial Japanese strain [11]. The most abundant transcript class found in small fish where the creatine kinase muscle-specific (CKM) genes, with both mitochondrial (mt) and cytosol (s) forms represented by several transcripts. Creatine kinase genes are involved in energy metabolism in shuttling phosphate (p) equivalents from ATP production in the mitochondria for storage as phosphocreatine (pCr) within the mitochondria and cytosol. Both mtCK and sCK forms can interconvert ATP and Cr to pCr and ADP. The location of these genes between the inner and outer mitochondrial matrix and throughout the cytosolic sarcoplasm facilitates and ensures a ready store of ATP to fuel actin/myosin filament sliding and cellular transport functions [12].

Increased CK expression is also a physiological hallmark of endurance training regimes, although in the context of the current study the very high levels seen for both cytosolic and mitochondrial forms of this gene may also be indicative of reactive oxygen stress imbalance. Abnormally high levels of these genes result from over-compensatory reactions to ROS inhibition of CK functions [12], which matches the gene expression profiles observed. Large fish had much higher expression for many ROS inhibiting enzymes such as glutathione peroxidases and reductases, indicating heightened stress response capabilities. Calcium voltage-gated channel genes (CACNG6) were also more up-regulated in small fish and could signify greater imbalance in calcium signaling in small fish. These genes are also associated with KEGG Cardiomyopathy classifications (Additional file 6).

The transcriptome profiles exhibited by large and small fish in this study share many similarities with exercise regime characteristics exhibited by athletes engaged in performance training. Small fish present profiles that share similarities in part with endurance/aerobic muscle performance [13] such as greater expression of genes related to β-oxidation of fats, higher PPRC1, CaMKII, and p53 expression. Small fish also show profiles more consistent with lowered energy status (i.e., higher AMP levels) such as heighted expression of phospholipase C which is linked to activation of AMPK. However, it should be noted that AMPK levels did not differ between large and small fish, although levels of TSC2 were higher in smaller fish. Conversely, larger fish show metabolic profiles that could be categorized as stress/resistance training-like. Characteristic of this syndrome is a greater reliance on glycogen metabolism with increased levels of genes related to glycogen breakdown (PYGM; AGL) and formation (GYS1), and enhanced gluconeogenesis (PC, GPI, FBP2).

Upstream signaling genes of Akt activation such as PI3K were also more abundant in large fish, and cytoskeletal components (e.g., collagen) appear elevated in large fish. The repertoire displayed in large fish, however, is not entirely consistent with enhanced protein accretion given the fact that genes such as MuRF1, calpain, NF-κB, TP53INP2, and TRAF-TNF receptor-associated genes appear to be more highly expressed in this size class. This suggests cellular apoptosis/turnover would be elevated in larger fish. Higher expression of cell cycle component genes such as cyclin D2, and AP-1 complex genes (junB, junD, c-Fos) in large fish conforms to a stress training model. However, the functions of the jun component transcriptional activators is complex and may have both inhibitory and inductive influences on cell cycle progression dependent upon other physiological signals [14]. Additionally, the higher activation of translational machinery subunits such as EIF4E in large fish is indicative of enhanced Akt signaling, however, a greater number of nucleobase processing components (e.g., EIF3A/EIF6, EIF4A, PABP, PAIP, RPL413, RPL37A, RPL11, RPL19, RPL36, RPL4, RPL5, RPL7, RPL12, RPL39 RPL41, RPS16, RPSA, RRS1, MRPL39, several DDX genes) reflecting elevated protein synthesis were in fact more abundant in smaller fish.

The observation that protein synthetic genes are expressed at higher levels in smaller fish is more consistent with gene expression profiles observed after eccentric muscle training regimes in humans [15]. Four different categories of expression profile were noted in the study by Kostek et al. [15] examining load-balanced eccentric and concentric gene expression profiles in young exercise-trained human males. In 2 of these four categories (stress and protein synthesis), genes or gene family member expression patterns were more similar between small rainbow trout and human eccentric muscle profiles (e.g., synaptogyrins, protein phosphatase 1-regulatory subunit 3C, CCHC-type zinc fingers, and ornithine decarboxylase antizyme), and concentric patterns matched those for large fish (e.g., metallothioneins). However, conflicting responses were also observed (e.g., glutathione-S-transferase, muscle pyruvate kinase, and four and a half LIM domain proteins). In two of the four categories, many homologous gene families were detected that exhibited a high abundance in both small and large fish including the most highly expressed genes, the tetraspanins. One gene class, sine oculis homeobox (SIX1) that showed marked upregulation during concentric exercise possessed a homolog that was also observed to be highly abundant in large fish (SIX3). SIX genes are known to regulate myogenesis [16], and although SIX3 is not currently recognized as a myogenic inducer, further research in fishes may reveal broader transcription factor functions for this family of genes. These findings match a model that suggests large fish may exhibit a greater affinity to concentric muscle contraction output. However, the second most highly upregulated gene detected during eccentric contractions [15] (musculoskeletal, embryonic nuclear protein 1, [MUSTN1]) was also more highly expressed in large fish.

Our current knowledge appears to limit the ability to match muscle growth trajectories with performance type expression patterns, if indeed such a match can be made. However, if future research can more directly identify key indicator genes of eccentric vs. concentric muscle physiology, it may be possible to use these gene expression profiles to predict fish growth, and couple these predictions to exercise performance. Large fish in general might be expected to show enhanced expression for both eccentric and concentric expression profiles as stress resistance training involves both types of movements. Nonetheless, it is an attractive hypothesis that suggests small fish may be able to sustain prolonged swimming activity in captivity, which they are expected to display in order to avoid confrontations with larger fish, by utilizing enhanced eccentric contractions which generate increased muscle force while utilizing less energy [17]. The suggestion that titin, a major anchoring protein in sarcomere assembly provides the rigidity necessary to support eccentric stiffness [18] also supports the hypothesis that small fish use enhanced eccentric force to drive their swimming given the fact that titin transcripts were more highly expressed in small vs. large fish (see section below on sarcomere assembly).

Caloric restrictions in fish, which likely afflict smaller fish to a greater degree than large fish, have also been observed to shift muscle fibre growth patterns leading to increased hypertrophy of fibres in transgenic coho salmon growth models [19]. However, larger fish appear to have greater white muscle fibre diameters than smaller fish since hypertrophy appears to minimize energetic costs during muscle growth [20]. Maximal fibre diameter differences attained do not appear to differ more than 2.5x between large and small fish within a species despite having body mass differences that could exceed an order of magnitude [20]. Diffusion constraints of oxygen nutrient supply to growing fibres likely force higher hyperplastic fibre recruitment in large fish [20], and increased hyperplastic growth in larger fish may in part be driven by greater expression of transcription factors (e.g., SIX3 and MUSTN1) [21] that enhance muscle fibre formation rather than hypertrophy. The fact that most fish species including salmonids express hyperplasic fibre formation throughout most of their lives may confound their associated functions with normal muscle physiology.

### Seasonal differences

When comparisons were made between the gene expression profiles of large and small fish within each growing season compared to the profiles obtained across the growing seasons combined, large fish were observed to have greater similarities to Dec. lot fish while small fish had greater similarities to Sept. lot fish. Of the 7164 distinct contigs discovered with significant expression differences (*P* < 0.05) across either size classes or seasons, 432 of these were shared between Sept. lot and small fish, and 562 were common to both large and Dec. fish. When Dec. fish were compared to the combined seasonal profiles for small fish, 68 contigs were identified as shared between both groups, while 60 contigs were shared between Sept. and large fish (Additional file 7). These differences were highly significant (2 × 2 contingency *χ*^2^ = 663.2; 1 df; *P* < 0.001).

The most highly abundant contigs in the Dec. lot fish were those associated with carbohydrate metabolism (e.g., PGK1, PGK2, ALDOA, PKM, ENO1, GAPDH), while Sept. lot fish exhibited high expression levels for genes associated with transport (ALB1, PVALB), transport and defense (HPX, SERPINA1, PON2, APOC2, CFB, RGC32), and signaling (NDRG2, FST, FOXL2). NDRG2 (N-myc downstream regulated gene 2) is of interest given recent findings that downregulation of this gene is linked to increased PIP_3_ signaling leading to enhanced Akt activation [22]. Hence increased upregulation of NDRG2 may be coupled to growth suppression via dampened Akt activation. Many of the contig reads that were highly voluminous in both the Dec. lot fish and large fish included many carbohydrate metabolism genes such as PFKM, PKM, GAPDH, ALDOA, FBP2, GPI, and LDHA as well as GADL1, SAA, APOE, MYH and HSPB6 indicative of transport, anatomical structure and stress response components. Smaller fish shared many CKM, CKMT1A, and MYH component contigs with Sept. lot fish and also notably NDRG2, FOXL2, and PON2 (Additional file 7; Additional file 8; Additional file 9).

### Sarcomere assembly

To address one of the main objectives of the study, we compared the possible expression differences of genes involved in the synthesis of muscle myofibrillar components between large and small fish as well as Sept. vs Dec. lot fish. Generally, greater differences were observed between the seasonal lots compared to the size categories of fish and these differences were also differentially portioned among the sarcomere components. For example, it appeared that a greater proportion of the Z-disc component genes (e.g., desmin, filamin α, β, γ, myozenin 1, 2, obscurin, actinin-associated LIM protein, and nebulin-anchoring protein (N-RAP) were abundant in Dec. lot, and large fish, while actin fibrillar components (e.g., tropomyosin, troponin C, troponin T2, nebulin, and telethonin) were more plentiful in Sept. lot fish. Larger fish also appeared to have higher upregulation of several sarcolemma costamere anchor proteins such as PDZ + PDLIM proteins, ITGB1/2, and Hsp90 stabilizing proteins. Myosin fibrillar components had different contig alignments that were up-regulated in both seasonal lots for genes such as myosin binding protein, myosin light chain, myosin heavy chain components, and titin (Additional file 10). Differences between large and small fish were not as pronounced as those present between Sept. and Dec. fish, although profiles in small fish suggest greater upregulation of the fibrillar components as evidenced by higher expression of MLC, titin, nebulin, and xin-actin compared to large fish. Myotubule fusion may also be higher in this class as evidenced by enhanced CD9 expression [23].

Large fish showed enhanced abundance of the M- and Z-line component gene reads which suggests that ‘scaffolding’ processes may be a general feature of faster growth, or perhaps more precisely during enhanced growth phases, as the M- and Z-line component genes were also general signatures in the fish during accelerated spring equinox growth. Conversely, the observation that certain myofibrillar component genes (e.g., titin, nebulin, MLC, obscurin) appear more highly upregulated in small fish indicates that key I- and A-line component genes may assemble at different times or rates between large and small fish. These findings are somewhat paradoxical given that anatomically larger fish have far greater component assemblies for all these gene classes than do small fish, so it remains to be assessed when these gene products are more highly expressed in large fish. One possibility is that circadian cycling of sarcomere components occurs, whereby larger fish may have enhanced expression for the myofibrillar gene class components during scotic periods of the diurnal cycle, which is preceded by preparatory ‘scaffolding’ (i.e., Z and M-line genes) recruitment during the photic period of the cycle. However, many aquaculture strains of salmonids are reared under constant light conditions as juveniles given that this procedure has been found to increase growth rates. Hence, if circadian rhythms influence growth assembly proteins then they may also be entrained by other external stimuli such as feeding schedules that need not necessarily be reliant upon differential light cycles.

Although not firmly established, it is generally regarded that the M-line component is the last structure to assemble, while Z-line components may initiate sarcomere assembly [24]. The addition of new sarcomere units appears to proceed bidirectionally within each myocyte through reorganization of costamere cytoskeletal sites along the sarcolemma [25]. Key genes involved in this reorganization are desmins, β-integrins, α-actinin, α-actinin LIM-associated proteins, and several heat-shock proteins and as indicated above, these classes have higher expression in larger fish suggesting that during the photic period which corresponds to peak energy acquisition phases of the diurnal cycle, scaffolding initiation is more enhanced in larger fish. An additional signature gene of increased Z-line scaffolding (N-RAP or nebulin-related anchor protein) [26] appears to be more highly expressed in Dec. lot fish, which would be indicative of an increased growth phase response. If Z- and M-line formations represent the nascent and ultimate steps, respectively, in sarcomere assembly, then the current findings are difficult to interpret as large fish have enhanced expression for component genes in both of these anatomical clusters suggestive of simultaneous formation. Whether fish differ from the mammalian model with regard to sarcomere assembly will require further research.

Greater exploration of the circadian and circannual regulation of gene expression differences in rainbow trout would be beneficial as previous studies have indicated the possibility that several carbohydrate and lipid metabolic genes will exhibit circadian fluctuations in expression [25, 27–29]. Circadian coupling to daily energy fluctuations and control of energy status through positive regulators of anabolic processes such as Akt and PI3K, and catabolic processes mediated via AMPK and PGC1α, has also been reported in mammalian models, [27]. Determining whether these processes occur in the same way in fishes requires investigation. Confirmation to expected profiles was evident in the current study, however, as noted by the postulated higher expression of PI3K in large fish and PGC1α in smaller fish. Additionally, the expression profiles for major proposed seasonal regulators of growth (e.g., POMC, MCR4, and leptin) also match expectations as evidenced by the inferred higher expression of leptin-receptors in Sept. fish based upon transcript reads.

### Comparison of RNAseq profiles with previous growth-related expression studies in salmonids

In a comparison of feed-restricted rainbow trout to control normal fed rainbow trout Kondo et al. [30] used both microarray data and quantitative PCR results to examine expression profiles between control or faster growing fish to those of restricted feed (RF) or slower growing fish. Microarray data from this study identified 18 contigs having > 3-fold expression differences between the two groups of fish, and qPCR retesting for 5 of these contigs supported the microarray dataset in three instances (i.e., G0S2, GAPDH, adiponectin). In addition, qPCR data also indicated higher expression levels for both growth hormone receptors I and II (GHR1 and GHR2) in RF-fish. Differential expression for 8 of the 20 genes was not evident within the current dataset, while for 11 of the 12 remaining genes faster growing fish had higher expression levels for G0S2, ALDOA, PFKM, PGAM, TPI (2 forms), FABP2, FABP3, and GAPDH, while slower-growing fish had higher expression levels for GHR1 and GHR2 similar to the findings from Kondo et al. [30]. For one gene, FBP, higher levels were detected in larger fish in the current study in contrast to higher levels found in RF-fish [30]. However, as mentioned previously, in the case of GAPDH and PFKM, it should be pointed out that both large and small fish had higher expression levels for these genes within different contig classes. These likely reflect different duplicate copies and/or splice variants of the genes, and highlight the complex nature of expression profiles that may be obtained from genomes having undergone duplication events.

In a similar study using coho salmon (*Oncorhynchus kisutch*) Overturf et al. [31] used 4 groups of fish to compare gene expression profiles among reduced ration (RR) vs. full-fed (FF) growth hormone-transgenic-crossed fish, domestic fish, and wild caught fish. The transgenic-crossed (TC) families were produced from matings between a growth hormone-transgenic line and wild fish. Growth rates of the domestic strain were very high and similar to FF-TC fish, and hence these two groups were considered models of faster growth compared to the RR-TC and wild fish. Twenty-one different genes were evaluated using qPCR assays, and the majority of these genes did not display any differences between large and small fish in the current study. Nine of these genes are regulators of early myogenesis, and their levels of expression were low in the current study. Differences were detected in both studies, however, among 6 genes regulating tissue growth and turnover. Calpain 1 (CAPN1) and FBP levels were higher in domestic fish and FF-TC fish [31] which match the profiles found in large fish in the current study. MuRF and IGFBP1 expression overlapped in 3 of the 4 coho salmon groups, and the data for IGFBP1 in the current study matches this finding. We found higher expression for different IGFBP1 contigs in both large and small fish in the current study, while MuRF expression for one contig was weakly higher in larger fish. The differential patterns found for IGFBP1, which are similar to those for GAPDH and PFKM, alludes to the possibility that different duplicated gene forms may possess different functions. For two genes, long form of calpastatin (CASTL) and atrogin (fbxO32), higher expression in domestic and FF-TC fish was reported [31], while we observed higher expression in small fish. In contrast to the coho salmon study, however, it was found that CAPN1 and CASTL levels were significantly higher in restricted-feed rainbow trout compared to faster growing controls [32]. These differences reinforce the need for more studies on the functional and triggered transcriptomic states that may characterize physiological growth states in salmonids. Furthermore, there is a need to interpret these findings using information from all gene copies within the genome.

### Sex

Examination of the gene expression profile differences between female and male fish revealed fairly similar levels of contigs detected with significant (FDR < 0.05) differences (292 vs. 354 contigs, respectively) (Additional file 11 and Additional file 12). However, Panther-GSEA revealed a greater diversity of pathways in males (e.g., catabolism and protein and metabolite turnover; and nucleobase processing). Overall, 153 GO terms were enriched in males, versus 29 in females (Additional file 13). Similar results were found from the microarray expression profile comparisons of all-female lots of rainbow trout compared to mixed-sex lots [33]. This led to the suggestion that greater variability in the expression profiles of males could be coupled to the more variable growth responses observed in mixed-sex lots compared to all-female lots derived from neomale (XX) sires [33]. The Broad Institute-GSEA indicated that both sexes had high expression of genes within muscle development, nucleotide/nucleoside processing, translation, transcription, apoptosis, cytoskeletal and anatomical development, and myofibril assembly pathways. Males, however, appeared to have profiles that more closely matched those of faster growing or larger rainbow trout in that they have elevated components for lipid metabolism. Enhanced representation of several component classes of genes related to transport were observed in females. Notable in this regard were several genes involved with calcium metabolism (e.g., two ryanodine receptors RYR1 and RYR3, as well as CACNB1) (Additional file 14; Additional file 15). Enhanced calcium levels have previously been reported in female compared to male fish, as Ca^++^ ion actively binds to vitellogenin and is important in egg development [34, 35]. Thus, even in 15-month old juveniles, calcium sequestration appears to differ between the sexes. This is also supported by the observation that calcitonin gene-related peptide (CRCP) receptors were more elevated in male fish, which would serve to decrease Ca^++^ levels. However, caution needs to be exercised in the interpretation of these findings as enhanced calcium metabolism was also a signature profile of smaller fish and our sampling of male and female fish was biased towards the inclusion of a higher number of small fish.

### SNP analysis

**Fig. 5:**
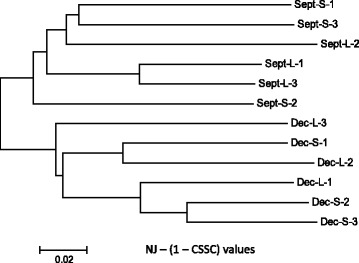
Neighbour-joining tree depicting the SNP allele genetic distances (1-CSSC values) of all 12 fish used in the analysis. September and December seasonal groupings are depicted as Sept. and Dec., respectively, while Large and Small fish are shown as L and S, respectively

**Fig. 6:**
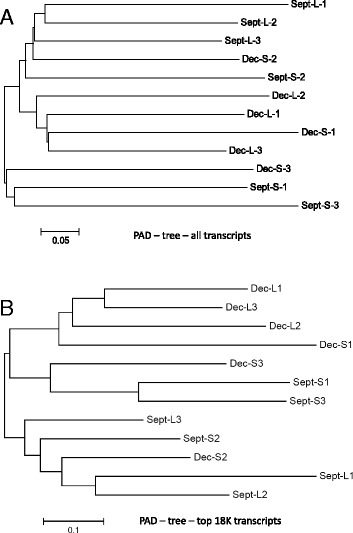
Pairwise Absolute Differences (PAD coefficient) expression levels based upon RPKM counts among all 12 fish sampled. RPKM values among 31,600 contigs, following edgeR filtering, are shown in Panel **a**, while Panel **b** depicts results from the most highly upregulated (RPKM ≥ 5) 18,756 contigs

Not unexpectedly, given the half-sib nature of the family origins within each seasonal grouping, the 1-CSSC value neighbor-joining analysis revealed that fish within each seasonal grouping were more closely related to one another compared to fish from the opposite seasonal lot (Fig. 5). The clustering of SNP allele distances among the sampled fish did not correspond to their pairwise absolute differences (PAD) in expression levels when all transcriptome contigs were evaluated (Fig. 6a). However, when the mostly highly expressed contigs (>5 RPKM in 2 or more fish) were evaluated, it was apparent that the large fish from the December lot shared greater expression affinities to one another (Fig. 6b) which is consistent with the findings among shared transcriptome contigs between seasonal and size groupings (Additional file 9). One of the small Dec. lot fish was also included within this PAD cluster suggesting stronger affinities in expression levels exist among seasonal grouping fish rather that size groupings.

The DI analysis identified 73 SNPs with high levels of genotypic differences (i.e., DI >0.89) between large and small fish in both seasonal lots. Sixty of these SNPs could be identified as falling within coding genes from alignments to the Bethelot et al. [36] rainbow trout genomic sequences. Comparisons to the differentially expressed genes within the current dataset indicated that 39 genes or member genes within families out of the 60 SNP locations identified were also coupled to differential expression levels (Additional file 16). Interestingly, the majority of the SNP locations identified were assigned to chromosomes 8, 22, and 26. We were unable to ascertain the FDR permutation expectations for SNPs having DI values greater than 0.89 given the fact that the parental genotypes were unknown. However, it is highly unlikely that the SNPs matching this cut-off would cluster within adjacent chromosome positions, if they are generated by chance.

To assess the reciprocal proclivity of the currently identified SNP locations and expression profiles to be coupled with reported SNP locations for growth in rainbow trout, we compared the current findings within this study to previously catalogued SNPs linked to growth and stress [37, 38]. Predominant stress and disease resistance QTL have previously been reported to be located on chromosome 8 [38–40] in rainbow trout, as well as stress-resistance QTL on chromosome 12 [38]. Ability to ameliorate the detrimental influences of stress upon growth should be coupled to the phenotypic size differences surveyed in this study. In the current study, three regions on the Berthelot et al. [36] ChrUn_8 assembly contained SNPs with significant associations with size, and the latter of these regions at 44.234 Mb contained a rho-activated kinase gene (ROCK) that may be coupled to the enhanced block of SNPs detected for stress resistance between 43.554 and 44.067 Mb [38].

The study by Salem et al. [37] cataloging growth-related SNPs utilized specific gene sequences and therefore we re-aligned the significant SNPs reported from that study to the rainbow trout transcriptome database. Homology searches using NextGENe were performed at 98-96 % identity allowing 1 mismatch seed. Unmatched reads were then tested down to 70 % identity. Of the 30 nuclear SNPs strongly coupled to growth differences in rainbow trout, we were able to match 27 of these SNPs to the contigs within the transcriptome reference database (Additional file 17). The 3 SNPs that could not be aligned may be from intronic or UTR regions close to the reported genes. Several of these SNPs aligned to different positions within the same gene class such that only 19 different gene sets were notable. All of these SNPs except for three (ATP5L2, TNNC2, and ACTN3-related) were coupled to transcripts that expressed significant expression differences in the current study (Part A of Additional file 17), or were representative of gene families that did exhibit significant expression differences (Part B of Additional file 17). Nine different mitochondrial genes were reported to possess one or more significant SNPs linked to growth differences in rainbow trout [37] and all of these SNPs were matched to transcripts with significant expression differences (Part A Additional file 17), except for ATP5J and MT-ND1. ATP5J did, however, possess two family members exhibiting significant up-regulation in large fish, while several MT-ND1 transcripts were up-regulated in both large and small fish and across both seasonal groupings (Part B Additional file 17).

Genetic mapping of the growth-related nuclear SNPs indicated that several different chromosomes (i.e., Chr-2, −4, −6, −10, −12, −16, −25, −Sex) harbour the SNPs localized with growth [37], and along with Chr-8 [38] represent some of the strongest growth-related QTL locations in rainbow trout. In addition to these locations, growth-related QTL have also been reported on chromosomes −1, −3, −5, −7, −14, −18, −21, and −28 [41] with Chr-2 and −12 QTL locations potentially spanning a large portion of the linkage group surveyed, suggesting the inclusion of several QTL. Interestingly, the high number of SNPs localized to Chr-16 [37] may also relate to a body weight QTL region described on Chr-16 by Wringe et al. [41]. This region was, however, centered in a putative 300 kb homology region to medaka chromosome 19 containing 5 genes (protein transport SEC24C; protein kinase C; homeobox sine oculus SIX3; glutamate dehydrogenase GLUD; and myozenin MYOZ1) [41] which all possess significantly higher expression in larger fish. The closest SNP marker (myosin binding protein C) was located 4 Mb away from this region, but a more precise homology to the medaka (*Oryzias latipes*) genome was difficult to assess as two MYBPC2 genes were mapped to Chr-16 [37] while only 1 copy is reported from medaka chromosome 19. In Dec. fish, SEC24C, GLUD and both MYOZ1 genes were upregulated along with synaptopodin 2-like protein (SYNPO2L) and protein phosphatase 1B (PPM1B) genes which also map to this region. This region shares homology to a region on stickleback (*Gasterosteus aculeatus*) chromosome group V spanning from 1.8 to 2.1 Mb.

## Conclusions

Gene expression patterns observed during active vs. declining seasonal growth periods in the life cycle of a fish may serve as a model in predicting size-related differences in gene expression patterns.  Environmental regimes that are associated with elevated growth rates induce gene expression responses that have a greater overlap with those observed in faster growing fish, while periods of declining environmentally induced growth are more similar to gene expression repertoires observed in smaller fish.  For certain gene classes, different duplicate copies and/or splice variants may also exist (e.g., GAPDH and PFKM) that have enhanced expression across both environments and size classes.  Such differences make the identification of ‘key signatory genes’ regulating growth difficult to assess without greater fundamental knowledge of expression patterns of distinct copies within varying environmental contexts, fish ages, and family backgrounds.  Several GO classifications may distinguish either faster vs. slower-growing rainbow trout but the overlap of some of these categories across seasons confounds their utility in defining growth differences.  However, even though certain GO groupings (e.g., Immune response) overlap in their representation within the two size classes, the specific genes with higher expression within this GO category differ between the size classes.  This suggests that evidence for distinct genes, or copies of genes within the same GO classification coupled to alternate phenotypic classes may become apparent as more research is conducted.  Repeated studies on the most highly up-regulated genes across environmental gradients (e.g., G0S2 in large fish and sCKM/ mtCKM in small fish) are needed.   Similarly, the dynamics of muscle fibre growth itself requires more research as current findings suggest enhanced hyperplastic induction and sarcomere scaffolding in large fish versus hypertrophic enhancement in small fish during photic periods of the circadian cycle.  However, these processes may vary on a circadian and circannual basis.

## Methods

### Fish

White muscle tissue obtained from Lyndon strain rainbow trout (Lyndon Fish Hatcheries, Inc. New Dundee, Ontario) served as the source of the cellular tissue analyzed in this study. Fish embryos were made at the hatchery and transported the same day to the Alma Aquaculture Research Station (R.R.#1, Elmira, Ontario) for subsequent care and rearing according to Animal Care Guidelines at the University of Guelph. All procedures related to rearing, feeding, weighing and measuring of the fish and final euthanasia were approved by the Animal Care Committee (ACC) at the University of Guelph. Two lots of fish were used for this study. The September (Sept.) lot was made Sept. 18, 2008 by crossing 3 females mated to a single male. Similarly, the December (Dec.) lot was made Dec. 10, 2008, by crossing 3 females to a single male. The offspring were reared at 9–11 °C under a natural photoperiod regime. When the fish in both lots were approximately 14.5 months of age, (Dec. 3/2009 for Sept. Lot and Feb. 23/2010 for Dec. Lot), the fish were euthanized, and white muscle tissue (epaxial below the dorsal fin but well above the lateral line to avoid red muscle tissue cross-contamination) was excised into small pellets and stored in an RNA preserving solution (3.75 M (NH_4_)_2_SO_4_, 10 mM EDTA, 25 mM Na_3_C_6_H_5_O_7_, adjusted to pH 5.2) for transport back to the University of Guelph where the tissue was stored at −80 °C until further use. Muscle plugs included connective, and interstitial tissue, as well as myotomal bundles, but care was taken to ensure that overlying dermal and epidermal layers were removed. Fish were size-selected such that one large and one small fish was sampled from each maternal parent, for a total of 3 large and 3 small individual size-selected fish from each growing season (Fig. 1). The size ranges between the large and small fish selected from any given family spanned a range of 364–403 g difference. Mean sizes for the Sept. Lot families at the time of sampling ranged from 250 to 392 g, while Dec. Lot families had mean sizes ranging from 370 to 492 g. The tissue samples were taken close to 12 noon on each sampling day. The fish used in this study represent a subset of the progeny sampled by the Kocmarek et al. [42] study, which may be referred to for documentation on the growth differences between the two seasonal lots.

### RNA sample preparation

Total RNA was isolated from the white muscle tissue using TRIzol (Invitrogen, Carlsbad, CA, USA) kits according to the manufacturer’s specifications, and the samples were stored at −80 °C until further use. Resulting RNA concentrations were determined using a Nanodrop 8000 spectrophotometer (Thermo Scientific, Waltham, MA, USA). The integrity of each sample was assessed by the detection of distinct 18S and 28S rRNA bands after agarose gel electrophoresis and with a Bio-Rad Experion system (Bio-Rad, Hercules, CA, USA) and only samples with an RNA quality index (RQI) of 8.0 or greater were submitted for sequencing.

### Library construction and illumina sequencing

Illumina sequencing was conducted by the Clinical Genomics Centre at Mount Sinai Hospital (Toronto, Ontario, Canada). Libraries were constructed according to the manufacturer’s protocols (Illumina, San Diego, CA, USA) with a brief description as follows: poly-A mRNA was isolated from total RNA and purified using poly-T oligo-attached beads. mRNA was fragmented into pieces using divalent cations under elevated temperature and copied into cDNA using reverse transcriptase and random primers. Following the ligation of adapters, cDNA templates were purified and amplified via PCR. Sequencing was conducted with a HiSeq 2000 platform (Illumina, San Diego, CA, USA) using 100 cycles of bar-coded paired-end sequencing in a single array lane. Image analysis, quality control, and base-calling was performed using Illumina’s sequence analysis software (Casava, v1.8.2). Raw reads have been deposited into NCBI’s Sequence Read Archive (SRA) under BioProject accession PRJNA209213 (SRP026259) and 12 BioSample accession numbers SAMN03946262, SAMN03946263….SAMN03946273, for fish 1–12, respectively, and Sample numbers SRS1019122, SRS1019123….SRS1019133, for fish numbers 1, 11, 2, 4, 12, 3, 6, 5, 10, 9, 8, and 7, respectively. The overall BioSample accession for all experiments was SAMN03944034.

### Filtering of illumina Hi-Seq reads

A total of 25,294.631 Mb of untrimmed DNA obtained from all 12 fish libraries was submitted for analysis. FastQC (http://www.bioinformatics.babraham.ac.uk/projects/fastqc) was used to identify overrepresented reads in the libraries. These were invariably assessed as Illumina adapter sequences with sample-specific barcode labels and reverse primer sequences. FastQC was also used to assess the overall quality parameters in the reads, and averages over the 12 fish indicated that read lengths ≥ 80 bp could contain Phred scores ≤ 20. We therefore set minimum read lengths at 80 bp following Trimmomatic filtering [43] using default parameter settings and an ILLUMINACLIP file containing the overly redundant sequences identified with FastQC.

### Sequence alignments

Sequence alignments were performed using NextGENe software (www.softgenetics.com) with run parameters set to ≥ 70 % homology, allowing large INDEL gaps, and 0 bp mismatches allowed in seed alignments (set to 40 bp footprint with a 12 bp sliding window) along with the other parameters set largely to default conditions. Prior to analysis the reference database was constructed with NextGENe’s ‘Build Index for WGA Tool’. Similarly, Illumina reads that remained paired following Trimmomatic filtering, were assembled into longer reads using default settings with NextGENe’s ‘Overlap Merger Tool’ (i.e., 16 bp overlap). This produced 5 sets of reads for each sample. Unpaired forward and reverse reads obtained from the Trimmomatic analysis; Merged paired reads; and Unmerged forward and reverse reads from the NextGENe Merger Tool (i.e., the remaining paired reads). Two transcriptome sequence alignments were then performed. The first, omitting the paired-read option used unmatched single reads and merged reads. The second using unmerged paired reads with the paired-read option active. Data from both analyses were then combined. All reads were aligned against a transcriptome reference database (described below). Most of the reads submitted (~70 % across all samples) were entered as single reads with paired reads representing a minority, given that the average size of the library clones were only 185 bp in length. Information on the number of merged, paired forward and reverse and unpaired reads for each fish is shown in Table 1. This analysis provided output on the number of reads assigned to each reference contig in the reference database and their associated RPKM values (reads aligned per thousand bp of reference contig per million reads aligned). However, for the generation of RPKM values all reads were entered in a single analysis given that the low percentage of paired reads remaining after merger did not bias assignment values.

**Table 1:** Statistics on the number and type of reads submitted for the transcriptome analysis across all 12 fish sampled in the study, and percentage of reads that were remained unmatched to the reference database

Fish #	Merged reads	Forward paired	Reverse paired	Forward unmatched	Reverse unmatched	Total reads^a^	% unmatched to reference^b^
1	2018242	503176	503177	324139	318662	5685638	6.20 %
2	7439746	1015334	1015328	954321	96864	18833115	8.93 %
3	6854344	7192502	7192481	1685389	1738903	31517963	8.88 %
4	4432100	401143	401139	514270	541751	10722503	8.47 %
5	3305888	131536	131537	389340	402963	7667152	9.36 %
6	7047261	267390	267382	948643	973747	16551684	9.20 %
7	3496699	3161006	3160977	789221	799779	14904381	8.55 %
8	6645641	139886	139911	854658	878138	15303875	8.24 %
9	6493912	276424	276430	840793	879280	15260751	7.47 %
10	7582472	991054	991050	1111996	1102768	19361812	6.88 %
11	4176263	5269718	5269704	1007420	1005070	20904438	7.94 %
12	8892226	1161514	1161518	1436781	1456964	23001229	7.52 %

^a^Includes 2x Merged Read Count

^b^Indicated the percentage of reads listed in the ‘Total Reads’ column that remained unmatched to the Berthelot et al. (2014), DFCI RTGI, SRR020739, SRR020740, and de novo contig build reference database compilation

### Analysis of gene expression differences

Gene expression differences were evaluated using the program edgeR [44] contained within the R bioconductor platform [45]. This program uses raw counts of the reads assigned to each reference contig for analysis. Both seasonal and size differences were evaluated as main effects in separate analyses using a two factor analysis model. To adjust for sampling variation prior to differential expression estimation glm trended dispersion and glm tagwise dispersion matrices were produced. The biological coefficient of variation obtained following adjustment was 0.29, and normalized data coefficients ranged from 1.18 to 0.82 across the 12 samples. Fish size (large vs. small) and sampling season (Sept. vs. Dec.) were treated as factors in the analysis, and all significant hits were adjusted for false positives using a Benjamini-Hochberg FDR threshold set to 0.05. However, all hits with a nominal *P* < 0.05 detected in the study are reported. Prior to analysis the data was filtered where row totals (i.e., number of fish with detectable gene expression) ≤ 3 hits, and when total counts were ≤ 1 CPM (counts per million) were excluded from the analysis. The rationale for this was based upon visual inspection of the data, in that several instances (gene IDs) were noted where all 3 fish in a particular size class x season group showed evidence for gene expression at a particular transcript, while all other fish sampled had no detectable expression. Given that read matches at 70 % identity were allowed in the analysis it is recognized that reads may have aligned to multiple contigs within any given gene class. Hence the current analysis was not targeted at discriminating individual gene copies, rather the focus was on the detection of expression levels within duplicated gene family sets.

### Transcriptome reference database

Illumina reads were aligned against gene sequence data from four sources. 1. The DFCI TIGR Gene Index reads for rainbow trout (Release 8.0; March 11, 2011) available at (ftp://occams.dfci.harvard.edu/pub/bio/tgi/data/) which contained 96,546 sequences compiled into contigs and singletons. 2. Two assemblies kindly provided by Jonathan Liu from SoftGenetics and these were based upon reads from 2 SRA indices (SRR020739 and SRR020740) (http://sra.dnanexus.com) which generated 36,702 and 29,174 contig builds, respectively. The SRR sequences can be obtained by entering the index number under the RUNS tab at the website. The sequences for these contig builds are provided in Additional file 18. The third source of gene reference transcripts was obtained from the gene annotation index for the rainbow trout genomic resource map [36]. A total of 45,582 annotated full length gene reads were available from their database. However, not all of the transcripts provided by these authors had accompanying annotations, and in total 46,585 reference transcripts totalling 53,591,493 bp were available for use. 4. Finally de novo assemblies from the unmatched reads to references sources 1–3 were included as reference contigs in a re-analysis of all read matches (see below).

Prior to building the transcriptome reference database in NextGENe the sequences within the DFCI database were first BLASTN aligned to one another and all shorter sequences falling within larger contigs with greater than 98 % identity and with a match exceeding 95 % of the shorter sequence length were removed from the database. A similar approach was applied to the two SRA libraries. Finally the reads from the DFCI database and the SRA databases were reciprocally BLASTN aligned to one another and shorter overlapping sequences were removed by the same criteria. This resulted in a final combined transcriptome reference database containing 147,718 contig references encompassing 109,680,021 bp of sequence. BLASTN parameters were set at: word size 11; gapopen 5; evalue e^−30^; gapextend 2; reward 2; penalty −3.

All unmatched reads to the above transcriptome reference database (sources 1–3) were further assembled using NextGENe deBrujn ‘de novo’ assembly settings. Contigs were assembled in sequential batches of word size W25, W33, W41, W49, W59, W65, W73, W81, and all separate assemblies from these parsed reads were re-assembled at W99 to build the final contigs. After filtering out duplicate reads (i.e., > 98 % identity within overlaps and an overlap spanning ≥ 95 % of the read length), and removing sequences that matched bacterial and parasite identities from the NCBI database (http://www.ncbi.nlm.nih.gov) a total of 54,759 additional contigs encompassing an additional 24,380,108 bp of sequence was added to the reference contig database. Average size of the new contigs added was 445 bp. A megablast BLASTN (default parameters except for a cut-off of e^−6^ and up to the 10 best matches reported) search for highly similar sequences against the NCBI non-redundant nucleotide database identified matches for 23,045 of newly assembled contigs. Their possible annotations are reported in Additional file 19. This transcriptome Shotgun Assembly project has been deposited at DDBJ/EMBL/GenGank under the accession GDKP00000000. The version described in this paper is the first version, GDKP01000000. This includes reference accession numbers GDKP01000001 – GDKP01054759.

Since the Berthelot et al. [36] annotated sequences encompassed fuller read lengths than those available from the other sources, all the pre-compiled contigs obtained from sources 1–2 above, were BLASTN aligned (using the parameter settings indicated above) to the Berthelot et al. reference database. Contigs with greater than 95 % identity and with read lengths matches exceeding 95 % of the contig length (=29,102 contigs) were considered overlapping regions to the Berthelot et al. reference genes. These contigs were then subsequently removed from the reference transcriptome database. The final compilation from all 4 sources (source 1 & 2 filtered against source 3, and the de novo assembled reads from source 4) contained 219,960 reference contigs encompassing 165,795,606 bp of sequence.

### Identification of contig assemblies and assignment of GO terms

Unidentified contig assemblies from the SRA reference databases and de novo constructed contigs were submitted to Blast2GO (http://www.blast2go.de/b2ghome) for assignment, annotation search, and GO category establishment, and were also BLASTN (default settings) searched against NCBI’s non-redundant vertebrate database for possible matches. GO assignments were re-analyzed using ANNEX supplementation feature for re-assessing terms. This search was only performed for transcript contigs that had significant matches following the edgeR analysis. Similarly, all significant hits detected within the study were re-annotated using the Blast2GO suite, or with the updated NCBI annotations, as some of the identifications listed within the TIGR database were based upon older annotations. Blast2GO was also used to assign GO functional terms to the differentially expressed contig hits. When contig regions were omitted by the Blast2GO searches they were re-queried using the AMIGO 2 software platform within the Gene Ontology database (http://amigo.geneontology.org/amigo) and matches to zebrafish genes were ranked highest. However, when zebrafish matches were not detected or simply assigned to the highest GO level (i.e., BP) GO term matches to *Homo sapiens* were accepted. Searches were only conducted within the Biological Process (BP) category. Abbreviated gene designations listed in this paper follows the HUGO Gene Nomenclature Committee (HGNC) designations (http://www.genenames.org).

Assignment to functional ‘GOSlim’ GO categories was performed using the GO Terms Classification Counter (CateGOrizer) (http://www.animalgenome.org/bioinfo/tools/countgo/). Prior to assignment all designations to the ‘Biological Process’ category were removed as this was the top level term and was considered redundant. Significant differences in counts between pairwise comparisons (Large vs Small and Dec. vs Sept.) were assessed using a backward elimination Heterogeneity G-tests (http://www.uoguelph/~rdanzman by following the links to the software directory). Re-exploration of the dataset was also undertaken with the software program REVIGO (http://revigo.irb.hr) [46]. This program provides greater detail on the GOSlim subcategories contributing to the highly up-regulated contigs within large and small fish and fish derived from the two seasonal groupings. For the analysis, the Relative Similarity (*RelSim*) option was set at medium (0.7) coverage. Gene Set Enrichment Analysis (GSEA) was determined using two sources: The first source was the GSEA module from the Broad Institute (http://www.broadinstitute.org/cancer/software/gsea/). This analysis was based upon overlaps within Biological Process and Canonical, KEGG, BIOCARTA, and REACTOME pathway categories. A second analysis using the gene ontology panther database (http://www.pantherdb.org) [47] utilized the Homo sapiens reference list as background for a Statistical Enrichment analysis using default settings. Comparisons were made between the rainbow size classes for Biological Process, Molecular Function, and Cellular Component categories within the ‘full GO’ designations. Both of the analysis methods used only the subsets of edgeR FDR significant identifiable rainbow trout orthologues to known HGNC annotated genes (large fish = 110 genes; small fish = 119 genes) as the source ID lists.

### Sex

Choice of matching the relative size differences between the largest and smallest fish selected from each of the paternal half-sib families used resulted in unequal sex ratios in the experimental fish chosen. In total 8 males and 4 females were chosen for the size x season study. However, to explore possible sex-related gene expression differences in these juvenile fish, we examined male vs. female gene expression differences in 4 fish from each sex. Two of the fish from each sex were obtained from the Sept. lot, while the other two were taken from the Dec. lot. To minimize size differences confounding the expression patterns all six small fish were analyzed, given that 3 of the 4 females sampled were from the designated ‘small’ size distribution. The one ‘large group’ female sampled was size-matched to a ‘large group’ male, but this male was from the opposite seasonal lot (i.e., the Sept. Lot, BioSample SAMN03946268, Sample SRS1019133). A GSEA (described above) was also performed on the FDR significant contig HGNC orthologues detected in males (=115 contigs) and females (=156 contigs).

### SNP genotyping

All 12 fish used for the RNAseq analysis were genotyped with the Affymetrix Axiom Trout Genotyping Array (#550571) which contains 57,501 SNP markers [48]. We were interested in assessing the relative similarity in SNP alleles among all the fish tested and whether these differences relate to their average overall levels of gene expression. In other words, we were interested in testing whether fish with greater differences in allelic identity were also more divergent in their shared gene expression profiles. To assess the degree of allele sharing among fish we calculated the average pairwise chromosome segment sharing coefficients (CSSC) among all fish (http://www.uoguelph.ca/~rdanzman with links to the software directory), which is a simple count of the proportion of shared alleles (0, 1 or 2, divided by 2) at all markers, divided by all the markers with informative pairwise genotypes. Distances were then obtained as: (1 – CSSC), and were compared using a Neighbour-Joining tree in MEGA 6.0 [49].

Pairwise Absolute Differences (PAD) in gene expression levels were calculated for all contigs between pairs of fish using only those contigs with detectable expression levels (RPKM) in at least 3 or more fish and CPM ≥ 1 (i.e., similar to edgeR filtering levels). This first analysis considered variation at 161,112 transcripts. A second analysis using only a subset of contigs with expression levels matching or higher than 5 RPKM (NextGENe analysis) for 2 or more fish was initiated to compare differences among the most highly expressed transcripts. This represented a total of 18,756 transcripts. To standardize PAD estimates across transcripts, RPKM expression levels recorded for each fish were adjusted to ratios according to the median expression level across all fish for any given transcript. Hence, adjusted RPKM ratio levels within each transcript ranged between 0 and 2. PAD values were then compared using a Neighbour-joining tree in MEGA 6.0 and compared to the 1-CSSC values.

To ascertain the degree of separation in SNP allele complements between small and large fish within each seasonal grouping, a Dissimilarity Index (DI) of observed SNP genotypes was calculated among all pairwise comparisons between small vs. large fish. This index simply assigns a value of 1 if the genotypes are not identical between the pair of individuals being compared and a value of 0 if they are identical. These values are averaged over all informative comparisons. While this index would not be useful in a random population survey lacking a known pedigree structure, in the current study, since each seasonal grouping was composed of paternal half-sib families, the index can highlight differences derived from paternally transmitted alleles. Since nine pairwise comparisons were made within each seasonal grouping (i.e., each small fish compared to all 3 large fish), we only considered DI values greater than 0.89 (i.e., 1 allowed similarity match within each seasonal group) to be representative of possible genomic regions bearing SNPs associated with genes influencing growth. Similarities in the SNPs meeting this criterion in both Dec. and Sept. lot fish were then reported. This analysis will undoubtedly largely underrepresent the actual QTL positions influencing growth in these two paternal half-sib lines. Firstly, female allelic variation was not properly tested and female allelic variation itself will potentially confound some of the DI scores. This would generate more type I error associations given that the sample sizes are extremely low. Secondly, if simply by chance a given SNP was homozygous in one of the two male parents, this region would be not be properly evaluated in the DI calculations. However, it was still considered of interest to compare the general genomic locations of identified significant SNPs to ascertain whether they support the finding from previous studies examining growth QTL in rainbow trout. VB Scripts for performing the PAD and DI calculations are available from the corresponding author upon request.

The flanking sequences surrounding the SNPs identified as having the largest DI values were then BLASTN aligned against the Berthelot et al. [36] chromosomal and unassigned sequences to obtain their most likely position within the rainbow trout genome. These locations were then cross-referenced to the gene annotation file provided by these researchers to establish possible gene identities for SNP markers. Additional information was then provided by one of us (YP pers. Comm.) as to the tentative location of these SNP positions on the new rainbow trout genome map.

## Availability of supporting data

Illumina HiSeq 2000 reads available from the NCBI database for bioproject SRP026259 at: www.ncbi.nlm.nih/sra?term=PRJNA209213. de novo assembly sequences available at: www.ncbi.nlm.nih.gov/Traces/wgs/wgsviewer.cgi?val=GDKP01.

## Additional files

Additional file 1:
**Box plots for the RPKM normalized contig reads for the 31,600 contigs passing edgeR filtering.** Note: the truncated representation of the upper quartile. Maximum RPKM value for the upper quartile is given above the graph. This corresponds to the Parvalbumin beta 1 gene (TC193113) in all fish except large fish 1 in the Dec. lot, where fast myotomal muscle actin (GSONMT00049647001) was the most highly expressed. Parvalbumin beta 1 was the 2^nd^ ranked contig expressed in this fish. (PPTX 85 kb) Additional file 2:
**Contigs with higher expression in large fish.** FDR significant genes used for the GSEA are highlighted in column E and their associated HGNC symbol is given in column I. (XLSX 197 kb) Additional file 3:
**Contigs with higher expression in small fish.** FDR significant genes used for the GSEA are highlighted in column E and their associated HGNC symbol is given in column I. (XLSX 206 kb) Additional file 4:
**Results from the Panther database gene set enrichment analysis (GSEA) comparing differences in GO categories between FDR significant contigs in large and small fish.** FDR significant categories for Biological Process, Molecular Function, and Cellular Components are shown. (XLSX 24 kb) Additional file 5:
**Results from the Broad Institute gene set enrichment analysis (GSEA) highlighting significant pathways and terms found in large fish.** FDR significant categories from the Biological Process and Canonical, KEGG, BIOCARTA, and REACTOME pathway categories are shown. (PDF 467 kb) Additional file 6:
**Results from the Broad Institute gene set enrichment analysis (GSEA) highlighting significant pathways and terms found in small fish.** FDR significant categories from the Biological Process and Canonical, KEGG, BIOCARTA, and REACTOME pathway categories are shown. (PDF 498 kb) Additional file 7:
**Shared contigs between size and season groups.** (XLSX 27 kb) Additional file 8:
**Contigs with higher expression in September fish.** FDR significant genes used for the GSEA are highlighted in column E and their associated HGNC symbol is given in column I. (XLSX 270 kb) Additional file 9:
**Contigs with higher expression in December fish.** FDR significant genes used for the GSEA are highlighted in column E and their associated HGNC symbol is given in column I. (XLSX 264 kb) Additional file 10:
**Contigs with higher expression levels for genes involved in sarcomere assembly process.** Comparisons between sizes (panel A) and seasons (panel B) are given. Note: if different contigs within a gene class exhibited higher expression in both large and small fish, or in both September and December fish, they are depicted in purple font. Figure adapted from: Garcia de la Serrana et al. 2012 (BMC Genomics 13: 181). (PPTX 735 kb) Additional file 11:
**Contigs with higher expression in female fish.** FDR significant genes used for the GSEA are highlighted in column E and their associated HGNC symbol is given in column I. (XLSX 244 kb) Additional file 12:
**Contigs with higher expression in male fish.** FDR significant genes used for the GSEA are highlighted in column E and their associated HGNC symbol is given in column I. (XLSX 255 kb) Additional file 13:
**Results from the Panther database gene set enrichment analysis (GSEA) comparing differences in GO categories between FDR significant contigs in female and male fish.** FDR significant categories for Biological Process are shown. (XLSX 23 kb) Additional file 14:
**Results from the Broad Institute gene set enrichment analysis (GSEA) highlighting significant pathways and terms found in female fish.** FDR significant categories from the Biological Process and Canonical, KEGG, BIOCARTA, and REACTOME pathway categories are shown. (PDF 707 kb) Additional file 15:
**Results from the Broad Institute gene set enrichment analysis (GSEA) highlighting significant pathways and terms found in male fish.** FDR significant categories from the Biological Process and Canonical, KEGG, BIOCARTA, and REACTOME pathway categories are shown. (PDF 528 kb) Additional file 16:
**SNPs surpassing the DI threshold cut-off and their localization to contigs with detected differences in gene expression between large and small fish and seasonally grouped fish.** (XLSX 20 kb) Additional file 17:
**Matches of growth-related SNPs reported in Salem et al. [** 37 **] to contigs exhibiting differential expression between large and small rainbow trout.** (XLSX 19 kb) Additional file 18:
**Fasta sequences for the contigs compiled from the SRR020739 and SRR020740 libraries.** (TXT 24095 kb) Additional file 19:
**Possible annotations for the de novo assembled contigs from the current study.** (XLSX 3810 kb)

## Abbreviations

General Abbreviations: ᅟ
ADP: adenosine diphosphate
AMP: adenosine monophosphate
ATP: adenosine triphosphate
BLASTN: basic local alignment search tool for nucleotides
BP: biological process
CPM: counts per million
CSSC: chromosome segment sharing coefficient
DFCI: Dana Farber Cancer Institute
DI: differentiation index
FDR: false discovery rate
GO: gene ontology
GSEA: gene set enrichment analysis
FF: full-fed
HGNC: HUGO Gene Nomenclature Committee
HUGO: Human Gene Ontology
MEGA: Molecular Evolutionary Genetics Analysis
MDS: multidimensional scaling
NCBI: National Centre for Biotechnology Information
PAD: pairwise absolute distance
PIP_3_: phosphatidylinositol (3,4,5)-triphosphate
QTL: quantitative trait locus
REVIGO: Reduce and Visualize Gene Ontology
ROS: reactive oxygen species
RF: restricted fed
RPKM: reads aligned per thousand bp of reference contig per million reads aligned
RR: reduced ration
SNP: single nucleotide polymorphism
SRA: Short Read Archive
TC: transgenic cross
TIGR: The Institute for Genomic Research
Gene Name Abbreviations: ᅟ
ACTN3: alpha 3 actinin
AGL: amylo-alpha-1,6-glucosidase, 4-alpha-glucanotransferase
Akt: protein kinase B
ALB1: albumin
ALDOA/ALDOB: fructose biphosphate aldolase (includes ALDOA; ALDOB)
AMPK: adenosine monophosphate kinase
AP-1: activating protein-1 complex
APOE: apolipoprotein E
APOC2: apolipoprotein C-II
ATGL: adipose triglyceride lipase
ATP5J/ATP5L2: mitochondrial complex V, ATP synthase subunits
c-FOS: basic leucine zipper proto-oncogene protein
cMyb: proto-oncogene transcription factor b
cMyc: proto-oncogene transcription factor c
CACNB1: calcium channel, voltage-dependent, beta 1 subunit
CACNG6: calcium channel, voltage-dependent, gamma subunit 6
CaMKII: calcium/calmodulin-dependent protein kinase II alpha
CAPN1: calpain I
CASTL: calpastatin (long form)
CFB: complement factor B
CKM: creatine kinase muscle
CKMT1A: creatine kinase, mitochondrial 1A
CRCP: calcitonin gene-related peptide receptor
DDX: Dead-box gene family
EIF: Elongation Initiation Factor genes (includes: EIF3A; EIF4A; EIF4E)
ENO1: enolase 1 (alpha)
FABP2/FABP3: fatty acid binding protein genes (includes, FABP2; FABP3)
FBP2: fructose-biphosphatase 2
FOXL2: forkhead box L2
FST: follistatin
G0S2: G0/G1 switch 2
GADL1: glutamate decarboxylase like 1
GAPDH: glyceraldehyde-3-phosphate dehydrogenase
GHR1/GHR2: growth hormone receptor genes (includes, GHR1; GHR2)
GLUD: glutamate dehydrogenase 1
GPI: glucose-6-phosphate isomerase
GYS1: glycogen synthase 1
HPX: hemopexin
Hsp90: heat shock cognate 90 kDa
HSPB6: heat shock protein family B (small) member 6
IGFBP1: insulin growth factor binding protein 1
IL4: interleukin 4
ITGB1/ITGB2: integrin beta genes (includes: ITGB1; ITGB2)
junB: transcription factor proto-oncogene junB
junD: transcription factor proto-oncogene junD
LDHA: lactate dehydrogenase A (muscle)
MCR4: melanocortin receptor 4
MLC: myosin light chain
MRPL39: mitochondrial ribosomal protein L39
MT-ND1: mitochondrial DNA subunit ND1
MuRF1: muscle ring-finger protein 1
MUSTN1: musculoskeletal, embryonic nuclear protein 1
MYBPC2: myosin binding protein C fast type
MYH: myosin heavy chain
MYOZ1: myozenin 1
NDRG2: NDRG (N-myc downstream regulated gene) family member 2
NF-κB: nuclear factor kappa-light-chain-enhancer of activated B cells
N-RAP: nebulin-related anchor protein
p38: p38 mitogen-activated protein kinase transcription factor
p53: p53 mitogen-activated protein kinase transcription factor
PABP: poly(A) binding protein
PAIP: poly(A) binding protein interacting protein
PC: pyruvate carboxylase
PDLIM: PDZ and LIM domain proteins
PDZ: PDZ domain proteins
PFKM: phosphofructokinase, muscle
PGC1α: peroxisome proliferator-activated receptor-gamma coactivator 1 alpha
PGAM: phosphoglycerate mutase
PGK1/PGK2: phosphoglycerate kinase genes (includes, PGK1; PGK2)
PI3K: phosphatidylinositol-4,5-bisphosphate 3-kinase catalytic subunit alpha
PKM: pyruvate kinase, muscle
POMC: proopiomelanocortin
PON2: paraoxonase 2
PPRC1: peroxisome proliferator-activated receptor gamma, coactivator-related 1
PPM1B: protein phosphatase, Mg2+/Mn2+ dependent 1B
PVALB: parvalbumin
PYGM: phosphorylase, glycogen, muscle
RGC32: regulator of cell cycle
ROCK: Rho-associated, coiled-coil containing protein kinase
RPL: L ribosomal protein genes (includes: RPL4; RPL5; RPL7; RPL11; RPL12; RPL19; RPL36; RPL37A; RPL39; RPL41)
RPS: S ribosomal protein genes (includes: RPSA; RPS16)
RRS1: ribosome biogenesis regulator
RYR1: ryanodine receptor 1 (skeletal)
RYR3: ryanodine receptor 3
SAA: serum amyloid A1
SEC24C: SEC24 homolog C, COPII coat complex component
SERPINA1: serpin peptidase inhibitor, clade A (alpha-1 antiproteinase, antitrypsin), member 1
SIX1/SIX3: sine oculis homeobox transcription factor genes (includes: SIX1; SIX3)
SYNPO2L: synaptopodin 2-like
TNNC2: troponin C type 2 (fast)
TP53INP2: tumor protein p53 inducible nuclear protein 2
TPI: triosephosphate isomerase
TRAF-TNF: TRAF and TNF receptor associated proteins
TSC2: tuberous sclerosis 2
