# Supplementary figures and images for: Transcriptome profiling in fast versus slow-growing rainbow trout across seasonal gradients
Source: BMC Genomics. 2016 Jan 15;17:60. doi: 10.1186/s12864-016-2363-5 (PMC4714434; doi:10.1186/s12864-016-2363-5)

## Slide 1
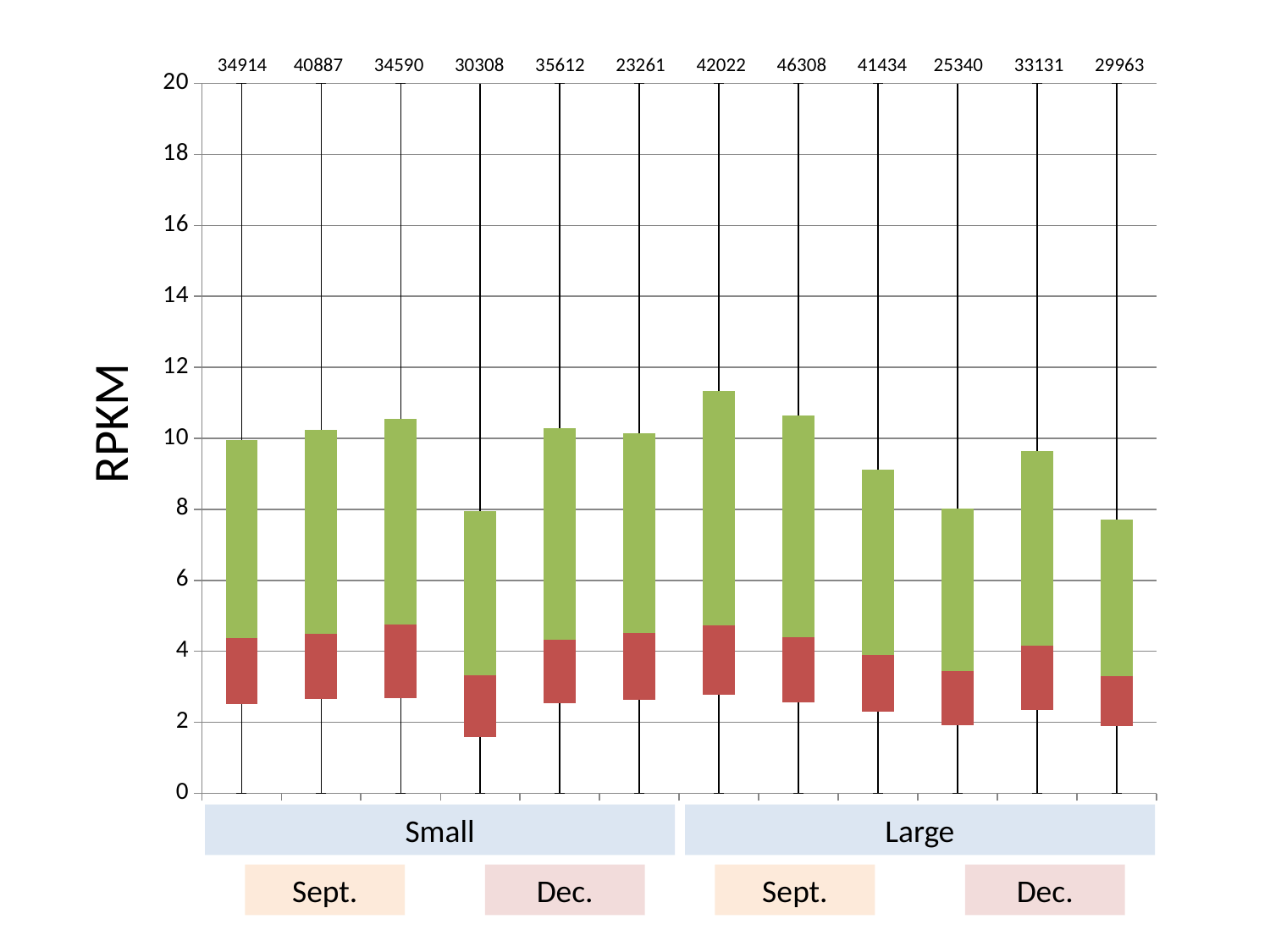

34914 40887 34590 30308 35612 23261 42022 46308 41434 25340 33131 29963
### Chart
| Category | | | |
|---|---|---|---|RPKM
Small
Large
Sept.
Dec.
Sept.
Dec.

Supplement: Additional file 1: — Box plots for the RPKM normalized contig reads for the 31,600 contigs passing edgeR filtering. Note: the truncated representation of the upper quartile. Maximum RPKM value for the upper quartile is given above the graph. This corresponds to the Parvalbumin beta 1 gene (TC193113) in all fish except large fish 1 in the Dec. lot, where fast myotomal muscle actin (GSONMT00049647001) was the most highly expressed. Parvalbumin beta 1 was the 2nd ranked contig expressed in this fish. (PPTX 85 kb) [file 12864_2016_2363_MOESM1_ESM.pptx]
