# Supplementary material for: Transcriptome profiling in fast versus slow-growing rainbow trout across seasonal gradients
Source: BMC Genomics. 2016 Jan 15;17:60. doi: 10.1186/s12864-016-2363-5 (PMC4714434; doi:10.1186/s12864-016-2363-5)
Supplement: Additional file 5: — Results from the Broad Institute gene set enrichment analysis (GSEA) highlighting significant pathways and terms found in large fish. FDR significant categories from the Biological Process and Canonical, KEGG, BIOCARTA, and REACTOME pathway categories are shown. (PDF 467 kb) [file 12864_2016_2363_MOESM5_ESM.pdf]

logged in as rdanzman@uoguelph.ca
logout
BROAD INSTITUTE

Compute Overlaps for Selected Genes

Converted 106 submitted identifiers into 101 entrez genes. click here for details.

Collections	# Overlaps Shown	# Gene Sets in Collections	# Genes in Comparison (n)	# Genes in Universe (N)
BP, CP, CP:BIOCARTA, CP:KEGG, CP:REACTOME	99	2145	101	45956

Click the gene set name to see the gene set page. Click the number of genes [in brackets] to download the list of genes.

Color bar shading from light green to black, where lighter colors indicate more significant FDR q-values (< 0.05) and black indicates less significant FDR q-values (>= 0.05).

Save to: Excel | GenomeSpace

Gene Set Name [# Genes (K)]	Description	# Genes in Overlap (k)	k/K	p-value	FDR q-value
KEGG_GLYCOLYSIS_GLUCONEOGENESIS [62]	Glycolysis / Gluconeogenesis	10		1.63 e^{-16}	3.49 e^{-13}
REACTOME_GLUCOSE_METABOLISM [69]	Genes involved in Glucose metabolism	10		5.08 e^{-16}	5.45 e^{-13}
REACTOME_GLUCONEOGENESIS [34]	Genes involved in Gluconeogenesis	8		7.1 e^{-15}	5.08 e^{-12}
REACTOME_GLYCOLYSIS [29]	Genes involved in Glycolysis	7		3.01 e^{-13}	1.61 e^{-10}
BIOCARTA_GLYCOLYSIS_PATHWAY [10]	Glycolysis Pathway	5		1.16 e^{-11}	4.97 e^{-9}
REACTOME_METABOLISM_OF_CARBOHYDRATES [247]	Genes involved in Metabolism of carbohydrates	10		2.1 e^{-10}	7.5 e^{-8}
KEGG_PENTOSE_PHOSPHATE_PATHWAY [27]	Pentose phosphate pathway	5		3.6 e^{-9}	1.1 e^{-6}
PID_HIF1_TFPATHWAY [66]	HIF-1-alpha transcription factor network	6		7.92 e^{-9}	2.12 e^{-6}
REACTOME_METABOLISM_OF_LIPIDS_AND_LIPOPROTEINS [478]	Genes involved in Metabolism of lipids and lipoproteins	11		9.42 e^{-9}	2.23 e^{-6}
KEGG_PPAR_SIGNALING_PATHWAY [69]	PPAR signaling pathway	6		1.04 e^{-8}	2.23 e^{-6}
REACTOME_PLATELET_ACTIVATION_SIGNALING_AND_AGGREGATION [208]	Genes involved in Platelet activation, signaling and aggregation	8		2.17 e^{-8}	4.23 e^{-6}
CELLULAR_CARBOHYDRATE_METABOLIC_PROCESS [126]	Genes annotated by the GO term GO:0044262. The chemical reactions and pathways involving carbohydrates, any of a group of organic compounds based of the	6		3.86 e^{-7}	6.26 e^{-5}

	general formula Cx(H2O)y, as carried out by individual cells.				
REGULATION_OF_BIOLOGICAL_QUALITY [419]	Genes annotated by the GO term GO:0065008. Any process that modulates the frequency, rate or extent of a biological quality. A biological quality is a measurable attribute of an organism or part of an organism, such as size, mass, shape, color, etc.	9		3.98 e^{-7}	6.26 e^{-5}
HOMEOSTATIC_PROCESS [207]	Genes annotated by the GO term GO:0042592. The biological processes involved in the maintenance of an internal equilibrium.	7		4.08 e^{-7}	6.26 e^{-5}
SYSTEM_PROCESS [563]	Genes annotated by the GO term GO:0003008. A biological process, occurring at the level of an organ system pertinent to the function of the organism. An organ system is a regularly interacting or interdependent group of organs or tissues that work together to carry out a given biological process.	10		4.98 e^{-7}	6.48 e^{-5}
APOPTOSIS_GO [431]	Genes annotated by the GO term GO:0006915. A form of programmed cell death induced by external or internal signals that trigger the activity of proteolytic caspases, whose actions dismantle the cell and result in cell death. Apoptosis begins internally with condensation and subsequent fragmentation of the cell nucleus (blebbing) while the plasma membrane remains intact. Other characteristics of apoptosis include DNA fragmentation and the exposure of phosphatidyl serine on the cell surface.	9		5.04 e^{-7}	6.48 e^{-5}
PROGRAMMED_CELL_DEATH [432]	Genes annotated by the GO term GO:0012501. Cell death resulting from activation of endogenous cellular processes.	9		5.14 e^{-7}	6.48 e^{-5}
CELL_DEVELOPMENT [577]	Genes annotated by the GO term GO:0048468. The process whose specific outcome is the progression of the cell over time, from its formation to the mature structure. Cell development does not include the steps involved in committing a	10		6.22 e^{-7}	7.42 e^{-5}

	cell to a specific fate.				
REACTOME_COMPLEMENT_CASCADE [32]	Genes involved in Complement cascade	4		7.54 e^{-7}	8.51 e^{-5}
REGULATION_OF_APOPTOSIS [341]	Genes annotated by the GO term GO:0042981. Any process that modulates the occurrence or rate of cell death by apoptosis.	8		9.4 e^{-7}	9.03 e^{-5}
REGULATION_OF_PROGRAMMED_CELL_DEATH [342]	Genes annotated by the GO term GO:0043067. Any process that modulates the frequency, rate or extent of programmed cell death, cell death resulting from activation of endogenous cellular processes.	8		9.6 e^{-7}	9.03 e^{-5}
REACTOME_HEMOSTASIS [466]	Genes involved in Hemostasis	9		9.61 e^{-7}	9.03 e^{-5}
KEGG_FRUCTOSE_AND_MANNOSE_METABOLISM [34]	Fructose and mannose metabolism	4		9.69 e^{-7}	9.03 e^{-5}
REACTOME_IMMUNE_SYSTEM [933]	Genes involved in Immune System	12		1.04 e^{-6}	9.31 e^{-5}
REACTOME_RESPONSE_TO_ELEVATED_PLATELET LET_CYTOSOLIC_CA2_ [89]	Genes involved in Response to elevated platelet cytosolic Ca2+	5		1.66 e^{-6}	1.43 e^{-4}
REACTOME_CELL_SURFACE_INTERACTIONS_AT_ AT_THE_VASCULAR_WALL [91]	Genes involved in Cell surface interactions at the vascular wall	5		1.86 e^{-6}	1.53 e^{-4}
REACTOME_PLATELET_ADHESION_TO_EXPOSED_ ED_COLLAGEN [12]	Genes involved in Platelet Adhesion to exposed collagen	3		2.23 e^{-6}	1.77 e^{-4}
ANATOMICAL_STRUCTURE_DEVELOPMENT [1013]	Genes annotated by the GO term GO:0048856. The biological process whose specific outcome is the progression of an anatomical structure from an initial condition to its mature state. This process begins with the formation of the structure and ends with the mature structure, whatever form that may be including its natural destruction. An anatomical structure is any biological entity that occupies space and is distinguished from its surroundings. Anatomical structures can be macroscopic such as a carpel, or microscopic such as an acrosome.	12		2.43 e^{-6}	1.86 e^{-4}
KEGG_AMINO_SUGAR_AND_NUCLEOTIDE_SUGAR_ AR_METABOLISM [44]	Amino sugar and nucleotide sugar metabolism	4		2.79 e^{-6}	2.06 e^{-4}
REACTOME_INNATE_IMMUNE_SYSTEM [279]	Genes involved in Innate Immune System	7		2.98 e^{-6}	2.13 e^{-4}
CARBOHYDRATE_METABOLIC_PROCESS [180]	Genes annotated by the GO term GO:0005975. The chemical reactions and pathways involving carbohydrates, any of a	6		3.09 e^{-6}	2.14 e^{-4}

	group of organic compounds based of the general formula Cx(H2O)y.				
SYSTEM_DEVELOPMENT [861]	Genes annotated by the GO term GO:0048731. The process whose specific outcome is the progression of an organismal system over time, from its formation to the mature structure. A system is a regularly interacting or interdependent group of organs or tissues that work together to carry out a given biological process.	11		$3.22\ e^{-6}$	$2.16\ e^{-4}$
REACTOME_LIPID_DIGESTION_MOBILIZATION_ ON_AND_TRANSPORT [46]	Genes involved in Lipid digestion, mobilization, and transport	4		$3.34\ e^{-6}$	$2.17\ e^{-4}$
MULTICELLULAR_ORGANISMAL_DEVELOPMENT [1049]	Genes annotated by the GO term GO:0007275. The biological process whose specific outcome is the progression of an organism over time from an initial condition (e.g. a zygote or a young adult) to a later condition (e.g. a multicellular animal or an aged adult).	12		$3.48\ e^{-6}$	$2.19\ e^{-4}$
BIOCARTA_CLASSIC_PATHWAY [14]	Classical Complement Pathway	3		$3.68\ e^{-6}$	$2.26\ e^{-4}$
KEGG_GLUTATHIONE_METABOLISM [50]	Glutathione metabolism	4		$4.68\ e^{-6}$	$2.79\ e^{-4}$
REGULATION_OF_DEVELOPMENTAL_PROCESS [440]	Genes annotated by the GO term GO:0050793. Any process that modulates the frequency, rate or extent of development, the biological process whose specific outcome is the progression of a multicellular organism over time from an initial condition (e.g. a zygote, or a young adult) to a later condition (e.g. a multicellular animal or an aged adult).	8		$6.16\ e^{-6}$	$3.57\ e^{-4}$
BIOCARTA_COMP_PATHWAY [19]	Complement Pathway	3		$9.73\ e^{-6}$	$5.49\ e^{-4}$
PID_IL4_2PATHWAY [65]	IL4-mediated signaling events	4		$1.34\ e^{-5}$	$7.38\ e^{-4}$
KEGG_COMPLEMENT_AND_COAGULATION_CASCAD CADES [69]	Complement and coagulation cascades	4		$1.7\ e^{-5}$	$9.13\ e^{-4}$
CELLULAR_HOMEOSTASIS [147]	Genes annotated by the GO term GO:0019725. The processes involved in the maintenance of an internal equilibrium at the level of the cell.	5		$1.93\ e^{-5}$	$1.01\ e^{-3}$
PID_LYMPHANGIOGENESIS_PATHWAY [25]	VEGFR3 signaling in lymphatic endothelium	3		$2.29\ e^{-5}$	$1.17\ e^{-3}$
PID_MYC_ACTIVPATHWAY [79]	Validated targets of C-MYC transcriptional activation	4		$2.91\ e^{-5}$	$1.45\ e^{-3}$
GLUCOSE_METABOLIC_PROCESS [28]	Genes annotated by the	3		$3.24\ e^{-5}$	$1.55\ e^{-3}$

	GO term GO:0006006. The chemical reactions and pathways involving glucose, the aldohexose gluco-hexose. D-glucose is dextrorotatory and is sometimes known as dextrose; it is an important source of energy for living organisms and is found free as well as combined in homo- and hetero-oligosaccharides and polysaccharides.				
REACTOME_LIPOPROTEIN_METABOLISM [28]	Genes involved in Lipoprotein metabolism	3		3.24 e^{-5}	1.55 e^{-3}
REACTOME_FATTY_ACID_TRIACYLGLYCEROL_AN_AND_KETONE_BODY_METABOLISM [168]	Genes involved in Fatty acid, triacylglycerol, and ketone body metabolism	5		3.67 e^{-5}	1.69 e^{-3}
PID_CMYB_PATHWAY [84]	C-MYB transcription factor network	4		3.7 e^{-5}	1.69 e^{-3}
ORGAN_DEVELOPMENT [571]	Genes annotated by the GO term GO:0048513. Development of a tissue or tissues that work together to perform a specific function or functions. Development pertains to the process whose specific outcome is the progression of a structure over time, from its formation to the mature structure. Organs are commonly observed as visibly distinct structures, but may also exist as loosely associated clusters of cells that work together to perform a specific function or functions.	8		3.97 e^{-5}	1.78 e^{-3}
REACTOME_GPVI_MEDIATED_ACTIVATION_CASCASCADE [31]	Genes involved in GPVI-mediated activation cascade	3		4.43 e^{-5}	1.91 e^{-3}
ALCOHOL_METABOLIC_PROCESS [88]	Genes annotated by the GO term GO:0006066. The chemical reactions and pathways involving alcohols, any of a class of alkyl compounds containing a hydroxyl group.	4		4.45 e^{-5}	1.91 e^{-3}
KEGG_PRION_DISEASES [35]	Prion diseases	3		6.41 e^{-5}	2.69 e^{-3}
PID_P38ALPHABETADOWNSTREAMPATHWAY [38]	Signaling mediated by p38-alpha and p38-beta	3		8.22 e^{-5}	3.39 e^{-3}
NEGATIVE_REGULATION_OF_CELLULAR_PROCESCESS [646]	Genes annotated by the GO term GO:0048523. Any process that stops, prevents or reduces the frequency, rate or extent of cellular processes, those that are carried out at the cellular level, but are not necessarily restricted to a single cell. For example, cell communication occurs among more than one cell,	8		9.37 e^{-5}	3.79 e^{-3}

	but occurs at the cellular level.				
IMMUNE_SYSTEM_PROCESS [332]	Genes annotated by the GO term GO:0002376. Any process involved in the development or functioning of the immune system, an organismal system for calibrated responses to potential internal or invasive threats.	6		$9.68\ e^{-5}$	$3.84\ e^{-3}$
PID_INTEGRIN3_PATHWAY [43]	Beta3 integrin cell surface interactions	3		$1.19\ e^{-4}$	$4.65\ e^{-3}$
NEGATIVE_REGULATION_OF_BIOLOGICAL_PROCESS [677]	Genes annotated by the GO term GO:0048519. Any process that stops, prevents or reduces the frequency, rate or extent of a biological process. Biological processes are regulated by many means; examples include the control of gene expression, protein modification or interaction with a protein or substrate molecule.	8		$1.29\ e^{-4}$	$4.95\ e^{-3}$
PID_HNF3BPATHWAY [45]	FOXA2 and FOXA3 transcription factor networks	3		$1.37\ e^{-4}$	$5.14\ e^{-3}$
BIOCARTA_FEEDER_PATHWAY [9]	Feeder Pathways for Glycolysis	2		$1.7\ e^{-4}$	$6.3\ e^{-3}$
KEGG_STARCH_AND_SUCROSE_METABOLISM [52]	Starch and sucrose metabolism	3		$2.11\ e^{-4}$	$7.36\ e^{-3}$
BIOCARTA_SARS_PATHWAY [10]	The SARS-coronavirus Life Cycle	2		$2.13\ e^{-4}$	$7.36\ e^{-3}$
REACTOME_PECAM1_INTERACTIONS [10]	Genes involved in PECAM1 interactions	2		$2.13\ e^{-4}$	$7.36\ e^{-3}$
TRIACYLGLYCEROL_METABOLIC_PROCESS [10]	Genes annotated by the GO term GO:0006641. The chemical reactions and pathways involving triacylglycerol, any triester of glycerol. The three fatty acid residues may all be the same or differ in any permutation. Triacylglycerols are important components of plant oils, animal fats and animal plasma lipoproteins.	2		$2.13\ e^{-4}$	$7.36\ e^{-3}$
MACROMOLECULE_CATABOLIC_PROCESS [137]	Genes annotated by the GO term GO:0009057. The chemical reactions and pathways resulting in the breakdown of a macromolecule, any large molecule including proteins, nucleic acids and carbohydrates.	4		$2.47\ e^{-4}$	$8.4\ e^{-3}$
REACTOME_DEVELOPMENTAL_BIOLOGY [396]	Genes involved in Developmental Biology	6		$2.51\ e^{-4}$	$8.41\ e^{-3}$
GLUCOSE_CATABOLIC_PROCESS [11]	Genes annotated by the GO term GO:0006007. The	2		$2.6\ e^{-4}$	$8.57\ e^{-3}$

	chemical reactions and pathways resulting in the breakdown of glucose, the aldohexose gluco-hexose.				
BIOCARTA_LECTIN_PATHWAY [12]	Lectin Induced Complement Pathway	2		$3.11\ e^{-4}$	$1.01\ e^{-2}$
REACTOME_CYTOKINE_SIGNALING_IN_IMMUNE_ NE_SYSTEM [270]	Genes involved in Cytokine Signaling in Immune system	5		$3.37\ e^{-4}$	$1.08\ e^{-2}$
NEGATIVE_REGULATION_OF_APOPTOSIS [150]	Genes annotated by the GO term GO:0043066. Any process that stops, prevents or reduces the frequency, rate or extent of cell death by apoptosis.	4		$3.48\ e^{-4}$	$1.1\ e^{-2}$
NEGATIVE_REGULATION_OF_PROGRAMMED_CELL ELL_DEATH [151]	Genes annotated by the GO term GO:0043069. Any process that stops, prevents or reduces the frequency, rate or extent of programmed cell death, cell death resulting from activation of endogenous cellular processes.	4		$3.57\ e^{-4}$	$1.11\ e^{-2}$
REACTOME_INTERFERON_GAMMA_SIGNALING [63]	Genes involved in Interferon gamma signaling	3		$3.72\ e^{-4}$	$1.14\ e^{-2}$
CHEMICAL_HOMEOSTASIS [155]	Genes annotated by the GO term GO:0048878. The biological processes involved in the maintenance of an internal equilibrium of a chemical.	4		$3.94\ e^{-4}$	$1.19\ e^{-2}$
PID_INTEGRIN1_PATHWAY [66]	Beta1 integrin cell surface interactions	3		$4.26\ e^{-4}$	$1.26\ e^{-2}$
STRIATED_MUSCLE_CONTRACTION_GO_0006941 [14]	Genes annotated by the GO term GO:0006941. A process whereby force is generated within striated muscle tissue, resulting in a change in muscle geometry. Force generation involves a chemo-mechanical energy conversion step. The chemo-mechanical energy conversion step is carried out by the actin/myosin complex activity, which generates force through ATP hydrolysis. Striated muscle is a type of muscle in which the repeating units (sarcomeres) of the contractile myofibrils are arranged in registry throughout the cell, resulting in transverse or oblique striations observable at the level of the light microscope.	2		$4.28\ e^{-4}$	$1.26\ e^{-2}$
REACTOME_INTERFERON_SIGNALING [159]	Genes involved in Interferon Signaling	4		$4.34\ e^{-4}$	$1.26\ e^{-2}$

REACTOME_HDL_MEDIATED_LIPID_TRANSPORT [15]	Genes involved in HDL-mediated lipid transport	2		4.93 e-4	1.41 e-2
REACTOME_TRANSCRIPTIONAL_REGULATION_OF_OF_WHITE_ADIPOCYTE_DIFFERENTIATION [72]	Genes involved in Transcriptional Regulation of White Adipocyte Differentiation	3		5.5 e-4	1.55 e-2
REACTOME_CHYLOMICRON_MEDIATED_LIPID_TR_TRANSPORT [16]	Genes involved in Chylomicron-mediated lipid transport	2		5.62 e-4	1.57 e-2
PID_AVB3_INTEGRIN_PATHWAY [75]	Integrins in angiogenesis	3		6.2 e-4	1.71 e-2
BIOCARTA_LAIR_PATHWAY [17]	Cells and Molecules involved in local acute inflammatory response	2		6.37 e-4	1.71 e-2
REACTOME_REGULATION_OF_KIT_SIGNALING [17]	Genes involved in Regulation of KIT signaling	2		6.37 e-4	1.71 e-2
RESPONSE_TO_CHEMICAL_STIMULUS [314]	Genes annotated by the GO term GO:0042221. A change in state or activity of a cell or an organism (in terms of movement, secretion, enzyme production, gene expression, etc.) as a result of a chemical stimulus.	5		6.69 e-4	1.77 e-2
REACTOME_INTEGRIN_CELL_SURFACE_INTERACRACTIONS [79]	Genes involved in Integrin cell surface interactions	3		7.22 e-4	1.89 e-2
BIOCARTA_MTA3_PATHWAY [19]	Downregulated of MTA-3 in ER-negative Breast Tumors	2		7.98 e-4	2.06 e-2
KEGG_ECM_RECEPTOR_INTERACTION [84]	ECM-receptor interaction	3		8.63 e-4	2.2 e-2
NEGATIVE_REGULATION_OF_DEVELOPMENTAL_PL_PROCESS [197]	Genes annotated by the GO term GO:0051093. Any process that stops, prevents or reduces the rate or extent of development, the biological process whose specific outcome is the progression of an organism over time from an initial condition (e.g. a zygote, or a young adult) to a later condition (e.g. a multicellular animal or an aged adult).	4		9.67 e-4	2.44 e-2
KEGG_FOCAL_ADHESION [201]	Focal adhesion	4		1.04 e-3	2.6 e-2
CELLULAR_CARBOHYDRATE_CATABOLIC_PROCESCESS [23]	Genes annotated by the GO term GO:0044275. The chemical reactions and pathways resulting in the breakdown of carbohydrates, any of a group of organic compounds based of the general formula Cx(H2O)y, as carried out by individual cells.	2		1.17 e-3	2.89 e-2
CELLULAR_CATABOLIC_PROCESS [212]	Genes annotated by the GO term GO:0044248. The chemical reactions and pathways resulting in the breakdown of substances, carried out by individual cells.	4		1.27 e-3	3.01 e-2

CARBOHYDRATE_CATABOLIC_PROCESS [24]	Genes annotated by the GO term GO:0016052. The chemical reactions and pathways resulting in the breakdown of carbohydrates, any of a group of organic compounds based of the general formula Cx(H2O)y.	2		$1.28\ e^{-3}$	$3.01\ e^{-2}$
PID_RHODOPSIN_PATHWAY [24]	Visual signal transduction: Rods	2		$1.28\ e^{-3}$	$3.01\ e^{-2}$
REACTOME_GROWTH_HORMONE_RECEPTOR_SIGNAGNALING [24]	Genes involved in Growth hormone receptor signaling	2		$1.28\ e^{-3}$	$3.01\ e^{-2}$
POSITIVE_REGULATION_OF_DEVELOPMENTAL_PL_PROCESS [218]	Genes annotated by the GO term GO:0051094. Any process that activates or increases the rate or extent of development, the biological process whose specific outcome is the progression of an organism over time from an initial condition (e.g. a zygote, or a young adult) to a later condition (e.g. a multicellular animal or an aged adult).	4		$1.4\ e^{-3}$	$3.27\ e^{-2}$
KEGG_GALACTOSE_METABOLISM [26]	Galactose metabolism	2		$1.5\ e^{-3}$	$3.46\ e^{-2}$
NEUROLOGICAL_SYSTEM_PROCESS [379]	Genes annotated by the GO term GO:0050877. The processes pertaining to the functions of the nervous system of an organism.	5		$1.54\ e^{-3}$	$3.51\ e^{-2}$
SKELETAL_DEVELOPMENT [103]	Genes annotated by the GO term GO:0001501. The process whose specific outcome is the progression of the skeleton over time, from its formation to the mature structure. The skeleton is the bony framework of the body in vertebrates (endoskeleton) or the hard outer envelope of insects (exoskeleton or dermoskeleton).	3		$1.55\ e^{-3}$	$3.51\ e^{-2}$
CATABOLIC_PROCESS [225]	Genes annotated by the GO term GO:0009056. The chemical reactions and pathways resulting in the breakdown of substances, including the breakdown of carbon compounds with the liberation of energy for use by the cell or organism.	4		$1.58\ e^{-3}$	$3.52\ e^{-2}$
CELLULAR_MACROMOLECULE_CATABOLIC_PROCEOCESS [104]	Genes annotated by the GO term GO:0044265. The chemical reactions and pathways resulting in the breakdown of a macromolecule, any large molecule including proteins, nucleic acids and carbohydrates, as carried	3		$1.6\ e^{-3}$	$3.53\ e^{-2}$

	out by individual cells.				
IMMUNE_RESPONSE [235]	Genes annotated by the GO term GO:0006955. Any immune system process that functions in the calibrated response of an organism to a potential internal or invasive threat.	4		1.85 e^{-3}	4.04 e^{-2}
ANTI_APOPTOSIS [118]	Genes annotated by the GO term GO:0006916. A process which directly inhibits any of the steps required for cell death by apoptosis.	3		2.29 e^{-3}	4.96 e^{-2}

Gene/geneset overlap matrix

Entrez Gene Id	Gene Symbol	KEGG_GLYCOLYSIS_GLUCONEOGENESIS	REACTOME_GLUCOSE_METABOLISM	REACTOME_GLUCONEOGENESIS	REACTOME_GLYCOLYSIS	BIOCARTA_GLYCOLYSIS_PATHWAY	REACTOME_METABOLISM_OF_CARBOHYDRATES	KEGG_PENTOSE_PHOSPHATE_PATHWAY	PID_HIF1_TFPATHWAY	REACTOME_METABOLISM_OF_LIPIDS_AND_LIPOPROTEINS	KEGG_PPAR_SIGNALING_PATHWAY	REACTOME_PLATELET_ACTIVATION_SIGNALING_AND_AGGREGATION	CELLULAR_CARBOHYDRATE_METABOLIC_PROCESS	REGULATION_OF_BIOLOGICAL_QUALITY	HOMEOSTATIC_PROCESS	SYSTEM_PROCESS	APOPTOSIS_GO	PROGRAMMED_CELL_DEATH	CELL_DEVELOPMENT	REACTOME_COMPLEMENT_CASCADE	REGULATION_OF_APOPTOSIS	REGULATION_OF_PROGRAMMED_CELL_DEATH	REACTOME_HEMOSTASIS	KEGG_FRUCTOSE_AND_MANNOSE_METABOLISM	REACTOME_IMMUNE_SYSTEM	REACTOME_RESPONSE_TO_ELEVATED_PLATELET_CYTOSOLIC_CA2_	REACTOME_CELL_SURFACE_INTERACTIONS_AT_THE_VASCULAR_WALL	REACTOME_PLATELET_ADHESION_TO_EXPOSED_COLLAGEN	ANATOMICAL_STRUCTURE_DEVELOPMENT	KEGG_AMINO_SUGAR_AND_NUCLEOTIDE_SUGAR_METABOLISM	REACTOME_INNATE_IMMUNE_SYSTEM	CARBOHYDRATE_METABOLIC_PROCESS	SYSTEM_DEVELOPMENT	REACTOME_LIPID_DIGESTION_MOBILIZATION_AND_TRANSPORT	MULTICELLULAR_ORGANISMAL_DEVELOPMENT	BIOCARTA_CLASSIC_PATHWAY	KEGG_GLUTATHIONE_METABOLISM	REGULATION_OF_DEVELOPMENTAL_PROCESS	BIOCARTA_COMP_PATHWAY	PID_IL4_2PATHWAY	KEGG_COMPLEMENT_AND_COAGULATION_CASCADES	CELLULAR_HOMEOSTASIS	PID_LYMPHANGIOGENESIS_PATHWAY	PID_MYC_ACTIVPATHWAY	GLUCOSE_METABOLIC_PROCESS	REACTOME_LIPOPROTEIN_METABOLISM	REACTOME_FATTY_ACID_TRIACYLGLYCEROL_AND_KETONE_BODY_METABOLISM	PID_CMYB_PATHWAY	ORGAN_DEVELOPMENT	REACTOME_GPVI_MEDIATED_ACTIVATION_CASCADE	ALCOHOL_METABOLIC_PROCESS	KEGG_PRION_DISEASES	PID_P38ALPHABETADOWNSTREAMPATHWAY	[illegible]
229	ALDOB	■	■	■	■	■	■	■					■											■								■													■						■			
2821	GPI	■	■	■	■	■	■	■						■																■																								
2023	ENO1	■	■	■	■	■	■		■					■																														■										■
7167	TPI1	■	■	■	■	■	■																	■																														
2597	GAPDH	■	■	■	■	■	■																																					■										

226	ALDOA
5224	PGAM2
8789	FBP2
5236	PGM1
3939	LDHA
7360	UGP2
123	PLIN2
7018	TF
4023	LPL
5360	PLTP
5346	PLIN1
3417	IDH1
348	APOE
84649	DGAT2
2194	FASN
50486	G0S2
5019	OXCT1
55224	ETNK2
2170	FABP3
2169	FABP2
6319	SCD
4067	LYN
2335	FN1
5216	PFN1
5341	PLEK
1278	COL1A2
1277	COL1A1
27159	CHIA
2876	GPX1
1134	CHRNA1
145226	RDH12

6288	SAA1
4502	MT2A
6496	SIX3
2779	GNAT1
7168	TPM1
2950	GSTP1
3875	KRT18
3313	HSPA9
7164	TPD52L1
4832	NME3
63924	CIDEC
5777	PTPN6
729	C6
730	C7
713	C1QB
3426	CFI
1520	CTSS
7295	TXN
1977	EIF4E
2634	GBP2
10631	POSTN
302	ANXA2
3860	KRT13
4440	MSI1
4281	MID1
2877	GPX2
1051	CEBPB
1050	CEBPA
4069	LYZ
3856	KRT8
3490	IGFBP7

6310	ATXN1
267	AMFR
4659	PPP1R12A
1522	CTSZ
3959	LGALS3BP
1915	EEF1A1
954	ENTPD2
23523	CABIN1
1757	SARDH
6462	SHBG
10611	PDLIM5
10962	MLLT11
116362	RBP7
116842	LEAP2
126393	HSPB6
134145	FAM173B
136319	MTPN
22837	COBLL1
27346	TMEM97
283985	FADS6
339896	GADL1
3697	ITIH1
389125	MUSTN1
3936	LCP1
51778	MYOZ2

5569	PKIA																																																					
58476	TP53INP2																																																					
6271	S100A1																																																					
6691	SPINK2																																																					
79639	TMEM53																																																					
79845	RNF122																																																					
79856	SNX22																																																					
84569	LYZL1																																																					
92521	SPECC1																																																					

Broad Home | Cancer Genomics

MSigDB database v4.0 updated May 31, 2013
GSEA/MSigDB web site v4.05 released June 8, 2014
