# Supplementary material for: Transcriptome profiling in fast versus slow-growing rainbow trout across seasonal gradients
Source: BMC Genomics. 2016 Jan 15;17:60. doi: 10.1186/s12864-016-2363-5 (PMC4714434; doi:10.1186/s12864-016-2363-5)
Supplement: Additional file 6: — Results from the Broad Institute gene set enrichment analysis (GSEA) highlighting significant pathways and terms found in small fish. FDR significant categories from the Biological Process and Canonical, KEGG, BIOCARTA, and REACTOME pathway categories are shown. (PDF 498 kb) [file 12864_2016_2363_MOESM6_ESM.pdf]

Compute Overlaps for Selected Genes

Converted 116 submitted identifiers into 107 entrez genes. click here for details.

Collections	# Overlaps Shown	# Gene Sets in Collections	# Genes in Comparison (n)	# Genes in Universe (N)
BP, CP, CP:BIOCARTA, CP:KEGG, CP:REACTOME	42	2145	107	45956

Click the gene set name to see the gene set page. Click the number of genes [in brackets] to download the list of genes.

Color bar shading from light green to black, where lighter colors indicate more significant FDR q-values (< 0.05) and black indicates less significant FDR q-values (>= 0.05).

Save to: Excel | GenomeSpace

Gene Set Name [# Genes (K)]	Description	# Genes in Overlap (k)	k/K	p-value	FDR q-value
KEGG_TIGHT_JUNCTION [134]	Tight junction	6		7.79 e^{-7}	9.15 e^{-4}
SYSTEM_PROCESS [563]	Genes annotated by the GO term GO:0003008. A biological process, occurring at the level of an organ system pertinent to the function of the organism. An organ system is a regularly interacting or interdependent group of organs or tissues that work together to carry out a given biological process.	10		8.53 e^{-7}	9.15 e^{-4}
KEGG_HYPERTROPHIC_CARDIOMYOPATHY_HCM [85]	Hypertrophic cardiomyopathy (HCM)	5		1.76 e^{-6}	1.26 e^{-3}
KEGG_DILATED_CARDIOMYOPATHY [92]	Dilated cardiomyopathy	5		2.61 e^{-6}	1.4 e^{-3}
REACTOME_METABOLISM_OF_AMINO_ACIDS_AND AND_DERIVATIVES [200]	Genes involved in Metabolism of amino acids and derivatives	6		7.9 e^{-6}	3.39 e^{-3}
KEGG_VIRAL_MYOCARDITIS [73]	Viral myocarditis	4		2.67 e^{-5}	9.14 e^{-3}
PROTEIN_METABOLIC_PROCESS [1231]	Genes annotated by the GO term GO:0019538. The chemical reactions and pathways involving a specific protein, rather than of proteins in general. Includes protein modification.	12		3.09 e^{-5}	9.14 e^{-3}
SYSTEM_DEVELOPMENT [861]	Genes annotated by the GO term GO:0048731. The	10		3.46 e^{-5}	9.14 e^{-3}

	process whose specific outcome is the progression of an organismal system over time, from its formation to the mature structure. A system is a regularly interacting or interdependent group of organs or tissues that work together to carry out a given biological process.				
KEGG_CARDIAC_MUSCLE_CONTRACTION [80]	Cardiac muscle contraction	4		$3.83\ e^{-5}$	$9.14\ e^{-3}$
SKELETAL_MUSCLE_DEVELOPMENT [31]	Genes annotated by the GO term GO:0007519. The developmental sequence of events leading to the formation of adult muscle that occurs in the anima. In vertebrate skeletal muscle the main events are: the fusion of myoblasts to form myotubes that increase in size by further fusion to them of myoblasts, the formation of myofibrils within their cytoplasm and the establishment of functional neuromuscular junctions with motor neurons. At this stage they can be regarded as mature muscle fibers.	3		$5.26\ e^{-5}$	$1.06\ e^{-2}$
CELLULAR_PROTEIN_METABOLIC_PROCESS [1117]	Genes annotated by the GO term GO:0044267. The chemical reactions and pathways involving a specific protein, rather than of proteins in general, occurring at the level of an individual cell. Includes protein modification.	11		$6.16\ e^{-5}$	$1.06\ e^{-2}$
CELLULAR_MACROMOLECULE_METABOLIC_PROCE OCESS [1131]	Genes annotated by the GO term GO:0044260. The chemical reactions and pathways involving macromolecules, large molecules including proteins, nucleic acids and carbohydrates, as carried out by individual cells.	11		$6.88\ e^{-5}$	$1.06\ e^{-2}$
MUSCLE_DEVELOPMENT [93]	Genes annotated by the GO term GO:0007517. The process whose specific outcome is the progression of the muscle over time, from its formation to the mature structure. The muscle is an organ consisting of a tissue made up of various elongated cells that are specialized to contract and thus to produce movement and mechanical work.	4		$6.91\ e^{-5}$	$1.06\ e^{-2}$

REGULATION_OF_TRANSLATION [93]	Genes annotated by the GO term GO:0006417. Any process that modulates the frequency, rate or extent of the chemical reactions and pathways resulting in the formation of proteins by the translation of mRNA.	4		6.91 e^{-5}	1.06 e^{-2}
TRANSMISSION_OF_NERVE_IMPULSE [189]	Genes annotated by the GO term GO:0019226. The sequential electrochemical polarization and depolarization that travels across the membrane of a nerve cell (neuron) in response to stimulation.	5		8.45 e^{-5}	1.21 e^{-2}
STRIATED_MUSCLE_DEVELOPMENT [40]	Genes annotated by the GO term GO:0014706. The process whose specific outcome is the progression of a striated muscle over time, from its formation to the mature structure. Striated muscle contain fibers that are divided by transverse bands into striations, and cardiac and skeletal muscle are types of striated muscle. Skeletal muscle myoblasts fuse to form myotubes and eventually multinucleated muscle fibers. The fusion of cardiac cells is very rare and can only form binucleate cells.	3		1.14 e^{-4}	1.53 e^{-2}
ANATOMICAL_STRUCTURE_DEVELOPMENT [1013]	Genes annotated by the GO term GO:0048856. The biological process whose specific outcome is the progression of an anatomical structure from an initial condition to its mature state. This process begins with the formation of the structure and ends with the mature structure, whatever form that may be including its natural destruction. An anatomical structure is any biological entity that occupies space and is distinguished from its surroundings. Anatomical structures can be macroscopic such as a carpel, or microscopic such as an acrosome.	10		1.32 e^{-4}	1.67 e^{-2}
BIOPOLYMER_METABOLIC_PROCESS [1684]	Genes annotated by the GO term GO:0043283. The chemical reactions and pathways involving biopolymers, long, repeating chains of monomers found in nature	13		1.51 e^{-4}	1.79 e^{-2}

	e.g. polysaccharides and proteins.				
NUCLEOBASENUCLEOSIDENUCLEOTIDE_AND_NUC NUCLEIC_ACID_METABOLIC_PROCESS [1244]	Genes annotated by the GO term GO:0006139. The chemical reactions and pathways involving nucleobases, nucleosides, nucleotides and nucleic acids.	11		$1.59\ e^{-4}$	$1.79\ e^{-2}$
MULTICELLULAR_ORGANISMAL_DEVELOPMENT [1049]	Genes annotated by the GO term GO:0007275. The biological process whose specific outcome is the progression of an organism over time from an initial condition (e.g. a zygote or a young adult) to a later condition (e.g. a multicellular animal or an aged adult).	10		$1.75\ e^{-4}$	$1.88\ e^{-2}$
KEGG_ARGININE_AND_PROLINE_METABOLISM [54]	Arginine and proline metabolism	3		$2.79\ e^{-4}$	$2.85\ e^{-2}$
PID_P53DOWNSTREAMPATHWAY [137]	Direct p53 effectors	4		$3.07\ e^{-4}$	$2.94\ e^{-2}$
REACTOME_CLASS_I_MHC_MEDIATED_ANTIGEN_ EN_PROCESSING_PRESENTATION [251]	Genes involved in Class I MHC mediated antigen processing & presentation	5		$3.15\ e^{-4}$	$2.94\ e^{-2}$
REACTOME_IMMUNE_SYSTEM [933]	Genes involved in Immune System	9		$3.43\ e^{-4}$	$3.07\ e^{-2}$
INTRACELLULAR_PROTEIN_TRANSPORT [145]	Genes annotated by the GO term GO:0006886. The directed movement of proteins in a cell, including the movement of proteins between specific compartments or structures within a cell, such as organelles of a eukaryotic cell.	4		$3.81\ e^{-4}$	$3.25\ e^{-2}$
ORGAN_DEVELOPMENT [571]	Genes annotated by the GO term GO:0048513. Development of a tissue or tissues that work together to perform a specific function or functions. Development pertains to the process whose specific outcome is the progression of a structure over time, from its formation to the mature structure. Organs are commonly observed as visibly distinct structures, but may also exist as loosely associated clusters of cells that work together to perform a specific function or functions.	7		$3.94\ e^{-4}$	$3.25\ e^{-2}$
REGULATION_OF_MULTICELLULAR_ORGANISMAL MAL_PROCESS [151]	Genes annotated by the GO term GO:0051239. Any process that modulates the frequency, rate or extent of an organismal process, the	4		$4.44\ e^{-4}$	$3.53\ e^{-2}$

	processes pertinent to the function of an organism above the cellular level; includes the integrated processes of tissues and organs.				
REGULATION_OF_BIOLOGICAL_QUALITY [419]	Genes annotated by the GO term GO:0065008. Any process that modulates the frequency, rate or extent of a biological quality. A biological quality is a measurable attribute of an organism or part of an organism, such as size, mass, shape, color, etc.	6		$4.61 e^{-4}$	$3.53 e^{-2}$
STRIATED_MUSCLE_CONTRACTION_GO_0006941 [14]	Genes annotated by the GO term GO:0006941. A process whereby force is generated within striated muscle tissue, resulting in a change in muscle geometry. Force generation involves a chemo-mechanical energy conversion step. The chemo-mechanical energy conversion step is carried out by the actin/myosin complex activity, which generates force through ATP hydrolysis. Striated muscle is a type of muscle in which the repeating units (sarcomeres) of the contractile myofibrils are arranged in registry throughout the cell, resulting in transverse or oblique striations observable at the level of the light microscope.	2		$4.8 e^{-4}$	$3.55 e^{-2}$
PROTEIN_TRANSPORT [157]	Genes annotated by the GO term GO:0015031. The directed movement of proteins into, out of, within or between cells.	4		$5.15 e^{-4}$	$3.59 e^{-2}$
INTRACELLULAR_TRANSPORT [280]	Genes annotated by the GO term GO:0046907. The directed movement of substances within a cell.	5		$5.19 e^{-4}$	$3.59 e^{-2}$
TRANSPORT [795]	Genes annotated by the GO term GO:0006810. The directed movement of substances (such as macromolecules, small molecules, ions) into, out of, within or between cells.	8		$5.61 e^{-4}$	$3.76 e^{-2}$
REGULATION_OF_CELLULAR_PROTEIN_METABOL BOLIC_PROCESS [162]	Genes annotated by the GO term GO:0032268. Any process that modulates the frequency, rate or extent of the chemical reactions and pathways involving a	4		$5.79 e^{-4}$	$3.76 e^{-2}$

	protein, occurring at the level of an individual cell.				
CELLULAR_COMPONENT_ASSEMBLY [298]	Genes annotated by the GO term GO:0022607. A cellular process that results in the assembly of a part of the cell.	5		6.87 e^{-4}	4.34 e^{-2}
REGULATION_OF_ACTION_POTENTIAL [17]	Genes annotated by the GO term GO:0001508. Any process that modulates the frequency, rate or extent of action potential creation, propagation or termination.	2		7.14 e^{-4}	4.38 e^{-2}
REGULATION_OF_PROTEIN_METABOLIC_PROCESCESS [173]	Genes annotated by the GO term GO:0051246. Any process that modulates the frequency, rate or extent of the chemical reactions and pathways involving a protein.	4		7.4 e^{-4}	4.38 e^{-2}
SYNAPTIC_TRANSMISSION [174]	Genes annotated by the GO term GO:0007268. The process of communication from a neuron to a target (neuron, muscle, or secretory cell) across a synapse.	4		7.56 e^{-4}	4.38 e^{-2}
GROWTH [77]	Genes annotated by the GO term GO:0040007. The increase in size or mass of an entire organism, a part of an organism or a cell.	3		7.92 e^{-4}	4.47 e^{-2}
BIOSYNTHETIC_PROCESS [470]	Genes annotated by the GO term GO:0009058. The energy-requiring part of metabolism in which simpler substances are transformed into more complex ones, as in growth and other biosynthetic processes.	6		8.39 e^{-4}	4.54 e^{-2}
TRANSLATION [180]	Genes annotated by the GO term GO:0006412. The chemical reactions and pathways resulting in the formation of a protein. This is a ribosome-mediated process in which the information in messenger RNA (mRNA) is used to specify the sequence of amino acids in the protein.	4		8.58 e^{-4}	4.54 e^{-2}
ORGANELLE_ORGANIZATION_AND_BIOGENESIS [473]	Genes annotated by the GO term GO:0006996. A process that is carried out at the cellular level which results in the formation, arrangement of constituent parts, or disassembly of any organelle within a cell.	6		8.67 e^{-4}	4.54 e^{-2}
CELLULAR_BIOSYNTHETIC_PROCESS [321]	Genes annotated by the GO term GO:0044249. The chemical reactions and	5		9.58 e^{-4}	4.89 e^{-2}

pathways resulting in the formation of substances, carried out by individual cells.

Gene/geneset overlap matrix

Entrez Gene Id	Gene Symbol	KEGG_TIGHT_JUNCTION	SYSTEM_PROCESS	KEGG_HYPERTROPHIC_CARDIOMYOPATHY_HCM	KEGG_DILATED_CARDIOMYOPATHY	REACTOME_METABOLISM_OF_AMINO_ACIDS_AND_DERIVATIVES	KEGG_VIRAL_MYOCARDITIS	PROTEIN_METABOLIC_PROCESS	SYSTEM_DEVELOPMENT	KEGG_CARDIAC_MUSCLE_CONTRACTION	SKELETAL_MUSCLE_DEVELOPMENT	CELLULAR_PROTEIN_METABOLIC_PROCESS	CELLULAR_MACROMOLECULE_METABOLIC_PROCESS	MUSCLE_DEVELOPMENT	REGULATION_OF_TRANSLATION	TRANSMISSION_OF_NERVE_IMPULSE	STRIATED_MUSCLE_DEVELOPMENT	ANATOMICAL_STRUCTURE_DEVELOPMENT	BIOPOLYMER_METABOLIC_PROCESS	NUCLEOBASENUCLEOSIDENUCLEOTIDE_AND_NUCLEIC_ACID_METABOLIC_PROCESS	MULTICELLULAR_ORGANISMAL_DEVELOPMENT	KEGG_ARGININE_AND_PROLINE_METABOLISM	PID_P53DOWNSTREAMPATHWAY	REACTOME_CLASS_I_MHC_MEDIATED_ANTIGEN_PROCESSING_PRESENTATION	REACTOME_IMMUNE_SYSTEM	INTRACELLULAR_PROTEIN_TRANSPORT	ORGAN_DEVELOPMENT	REGULATION_OF_MULTICELLULAR_ORGANISMAL_PROCESS	REGULATION_OF_BIOLOGICAL_QUALITY	STRIATED_MUSCLE_CONTRACTION_GO_0006941	PROTEIN_TRANSPORT	INTRACELLULAR_TRANSPORT	TRANSPORT	REGULATION_OF_CELLULAR_PROTEIN_METABOLIC_PROCESS	CELLULAR_COMPONENT_ASSEMBLY	REGULATION_OF_ACTION_POTENTIAL	REGULATION_OF_PROTEIN_METABOLIC_PROCESS	SYNAPTIC_TRANSMISSION	GROWTH	BIOSYNTHETIC_PROCESS	TRANSLATION	ORGANELLE_ORGANIZATION_AND_BIOGENESIS	CELLULAR_BIOSYNTHETIC_PROCESS	Entrez	Source	Gene Description
4625	MYH7	X	X	X	X		X		X	X				X				X			X						X	X	X			X	X									X			S	myosin, heavy chain 7, cardiac muscle, beta
4633	MYL2	X	X	X	X					X																		X		X															S	myosin, light chain 2, regulatory, cardiac, slow
4620	MYH2	X					X																																						S	myosin, heavy chain 2, skeletal muscle, adult
4619	MYH1	X					X																																						S	myosin, heavy chain 1, skeletal muscle, adult
4622	MYH4	X					X																																						S	myosin, heavy chain 4,

		skeletal muscle
23136	EPB41L3	erythrocyte membrane protein band 4.1-like 3
7273	TTN	titin
70	ACTC1	actin, alpha, cardiac muscle 1
928	CD9	CD9 molecule
4155	MBP	myelin basic protein
5376	PMP22	peripheral myelin protein 22
4359	MPZ	myelin protein zero
2890	GRIA1	glutamate receptor, ionotropic, AMPA 1
6329	SCN4A	sodium channel, voltage-gated, type IV, alpha subunit
59285	CACNG6	calcium channel, voltage-dependent, gamma subunit 6
5704	PSMC4	proteasome (prosome, macropain) 26S subunit, ATPase, 4
26227	PHGDH	phosphoglycerate dehydrogenase
1158	CKM	creatine kinase, muscle
262	AMD1	adenosylmethionine decarboxylase 1
548596	CKMT1A	creatine kinase, mitochondrial 1A
36	ACADSB	acyl-CoA dehydrogenase, short/branched chain
322	APBB1	amyloid beta (A4) precursor protein-binding, family B, member 1 (Fe65)
1974	EIF4A2	eukaryotic translation initiation factor 4A2
80381	CD276	CD276 molecule
51247	PAIP2	poly(A) binding protein interacting protein 2
3276	PRMT1	protein arginine methyltransferase 1
7326	UBE2G1	ubiquitin-conjugating enzyme E2G 1
28951	TRIB2	tribbles homolog 2 (Drosophila)
23192	ATG4B	ATG4 autophagy related 4 homolog B (S. cerevisiae)
10575	CCT4	chaperonin containing TCP1, subunit 4 (delta)
668	FOXL2	forkhead box L2
6840	SVIL	supervillin
2690	GHR	growth hormone receptor
1310	COL19A1	collagen, type XIX, alpha 1
10153	CEBPZ	CCAAT/enhancer binding protein (C/EBP), zeta
51602	NOP58	NOP58 ribonucleoprotein homolog (yeast)
2969	GTF2I	general transcription factor IIi
6944	VPS72	vacuolar protein sorting 72 homolog (S. cerevisiae)

54880	BCOR	S	BCL6 corepressor
10521	DDX17	S	DEAD (Asp-Glu-Ala-Asp) box polypeptide 17
6100	RP9	S	retinitis pigmentosa 9 (autosomal dominant)
472	ATM	S	ataxia telangiectasia mutated
159	ADSS	S	adenylosuccinate synthase
7249	TSC2	S	tuberous sclerosis 2
54541	DDIT4	S	DNA-damage-inducible transcript 4
948	CD36	S	CD36 molecule (thrombospondin receptor)
51676	ASB2	S	ankyrin repeat and SOCS box containing 2
9730	VPRBP	S	Vpr (HIV-1) binding protein
714	C1QC	S	complement component 1, q subcomponent, C chain
23303	KIF13B	S	kinesin family member 13B
26088	GGA1	S	golgi-associated, gamma adaptin ear containing, ARF binding protein 1
9135	RABEP1	S	rabaptin, RAB GTPase binding effector protein 1
5833	PCYT2	S	phosphate cytidylyltransferase 2, ethanolamine
4703	NEB	S	nebulin
5313	PKLR	S	pyruvate kinase, liver and RBC
5208	PFKFB2	S	6-phosphofructo-2-kinase/fructose-2,6-biphosphatase 2
22978	NT5C2	S	5'-nucleotidase, cytosolic II
5507	PPP1R3C	S	protein phosphatase 1, regulatory subunit 3C
3484	IGFBP1	S	insulin-like growth factor binding protein 1
3628	INPP1	S	inositol polyphosphate-1-phosphatase
113026	PLCD3	S	phospholipase C, delta 3
340267	COL28A1	S	collagen, type XXVIII, alpha 1
57447	NDRG2	S	NDRG family member 2
3371	TNC	S	tenascin C
6168	RPL37A	S	ribosomal protein L37a
23163	GGA3	S	golgi-associated, gamma adaptin ear containing, ARF binding protein 3
9110	MTMR4	S	myotubularin related protein 4
100507436	MICA	S	MHC class I polypeptide-related sequence A
154091	SLC2A12	S	solute carrier family 2 (facilitated glucose transporter), member 12
55749	CCAR1	S	cell division cycle and apoptosis regulator 1
219770	GJD4	S	gap junction protein, delta 4, 40.1kDa
977	CD151	S	CD151 molecule (Raph

			blood group)
3308	HSPA4	S	heat shock 70kDa protein 4
7145	TNS1	S	tensin 1
10241	CALCOCO2	S	calcium binding and coiled-coil domain 2
8780	RIOK3	S	RIO kinase 3 (yeast)
129446	XIRP2	S	xin actin-binding repeat containing 2
221476	PI16	S	peptidase inhibitor 16
23082	PPRC1	S	peroxisome proliferator-activated receptor gamma, coactivator-related 1
23212	RRS1	S	RRS1 ribosome biogenesis regulator homolog (S. cerevisiae)
27146	FAM184B	S	family with sequence similarity 184, member B
2813	GP2	S	glycoprotein 2 (zymogen granule membrane)
28987	NOB1	S	NIN1/RPN12 binding protein 1 homolog (S. cerevisiae)
30836	DNTTIP2	S	deoxynucleotidyltransferase, terminal, interacting protein 2
339291	LRRC30	S	leucine rich repeat containing 30
388228	SBK1	S	SH3-binding domain kinase 1
51105	PHF20L1	S	PHD finger protein 20-like 1
51673	TPPP3	S	tubulin polymerization-promoting protein family member 3
5445	PON2	S	paraoxonase 2
55075	UACA	S	uveal autoantigen with coiled-coil domains and ankyrin repeats
55127	HEATR1	S	HEAT repeat containing 1
55720	TSR1	S	TSR1, 20S rRNA accumulation, homolog (S. cerevisiae)
56997	ADCK3	S	aarF domain containing kinase 3
5816	PVALB	S	parvalbumin
64518	TEKT3	S	tektin 3
646643	SBK2	S	SH3-binding domain kinase family, member 2
64779	MTHFSD	S	methenyltetrahydrofolate synthetase domain containing
64794	DDX31	S	DEAD (Asp-Glu-Ala-Asp) box polypeptide 31
79026	AHNAK	S	AHNAK nucleoprotein
79442	LRRC2	S	leucine rich repeat containing 2
79670	ZCCHC6	S	zinc finger, CCHC domain containing 6
80060	GNL3LP1	S	guanine nucleotide binding protein-like 3 (nucleolar)-like pseudogene 1
80315	CPEB4	S	cytoplasmic polyadenylation element binding protein 4

8209	C21orf33																																												S	chromosome 21 open reading frame 33
831	CAST																																												S	calpastatin
9099	USP2																																												S	ubiquitin specific peptidase 2
9144	SYNGR2																																												S	synaptogyrin 2

MSigDB database v4.0 updated May 31, 2013
GSEA/MSigDB web site v4.05 released June 8, 2014
