# Supplementary material for: Transcriptome profiling in fast versus slow-growing rainbow trout across seasonal gradients
Source: BMC Genomics. 2016 Jan 15;17:60. doi: 10.1186/s12864-016-2363-5 (PMC4714434; doi:10.1186/s12864-016-2363-5)
Supplement: Additional file 10: — Contigs with higher expression levels for genes involved in sarcomere assembly process. Comparisons between sizes (panel A) and seasons (panel B) are given. Note: if different contigs within a gene class exhibited higher expression in both large and small fish, or in both September and December fish, they are depicted in purple font. Figure adapted from: Garcia de la Serrana et al. 2012 (BMC Genomics 13: 181). (PPTX 735 kb) [file 12864_2016_2363_MOESM10_ESM.pptx]

## Slide 1
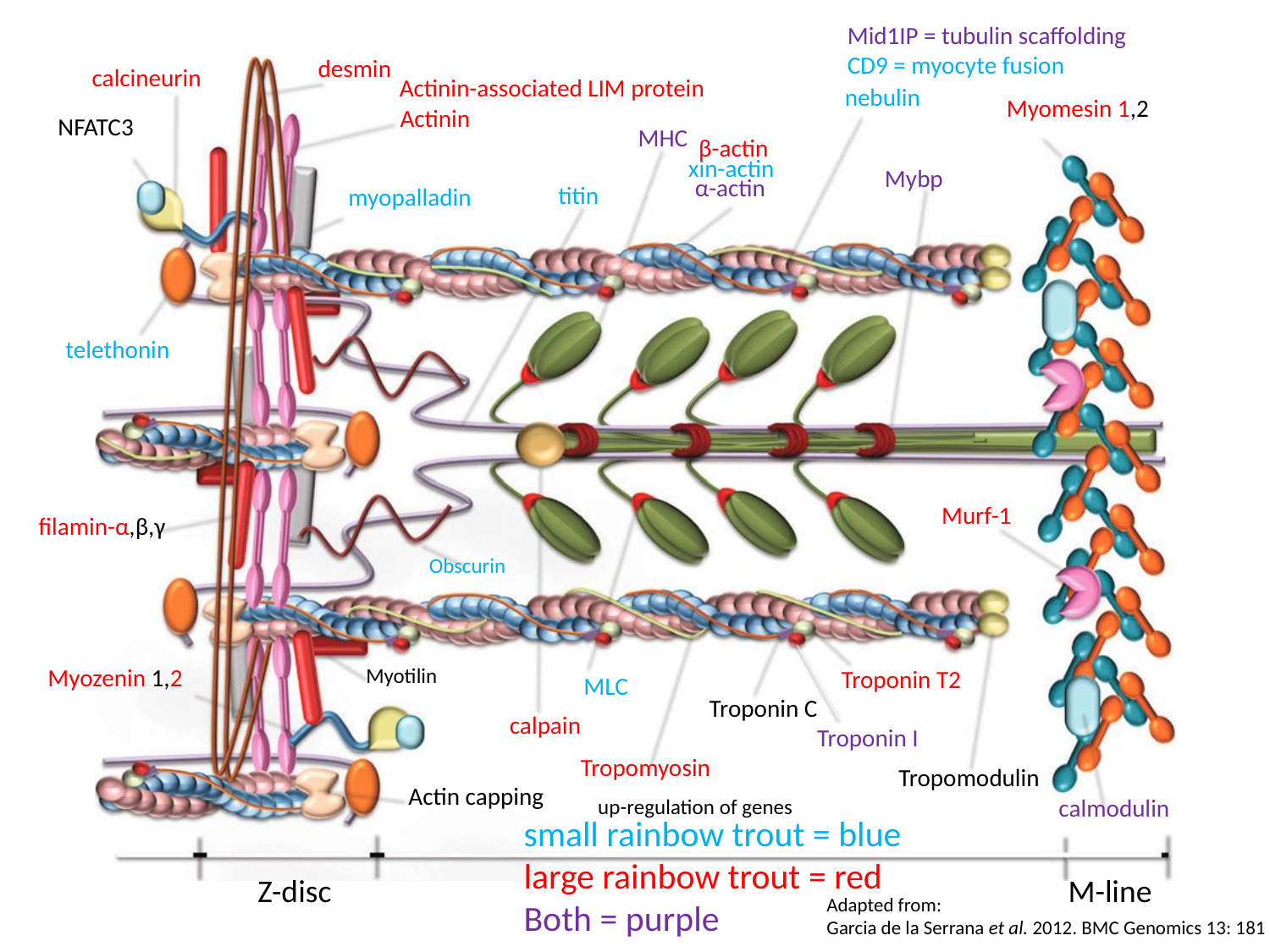

Mid1IP = tubulin scaffolding
CD9 = myocyte fusion
desmin
calcineurin
nebulin
Myomesin 1,2
Actinin
NFATC3
MHC
Mybp
α-actin
titin
myopalladin
telethonin
Murf-1
filamin-α,β,γ
Obscurin
Myozenin 1,2
Myotilin
Troponin T2
MLC
Troponin C
calpain
Troponin I
Tropomyosin
Tropomodulin
Actin capping
calmodulin
Z-disc
M-line
Actinin-associated LIM protein
β-actin
xin-actin
up-regulation of genes
small rainbow trout = blue
large rainbow trout = red
Both = purple
Adapted from:
Garcia de la Serrana et al. 2012. BMC Genomics 13: 181

## Slide 2
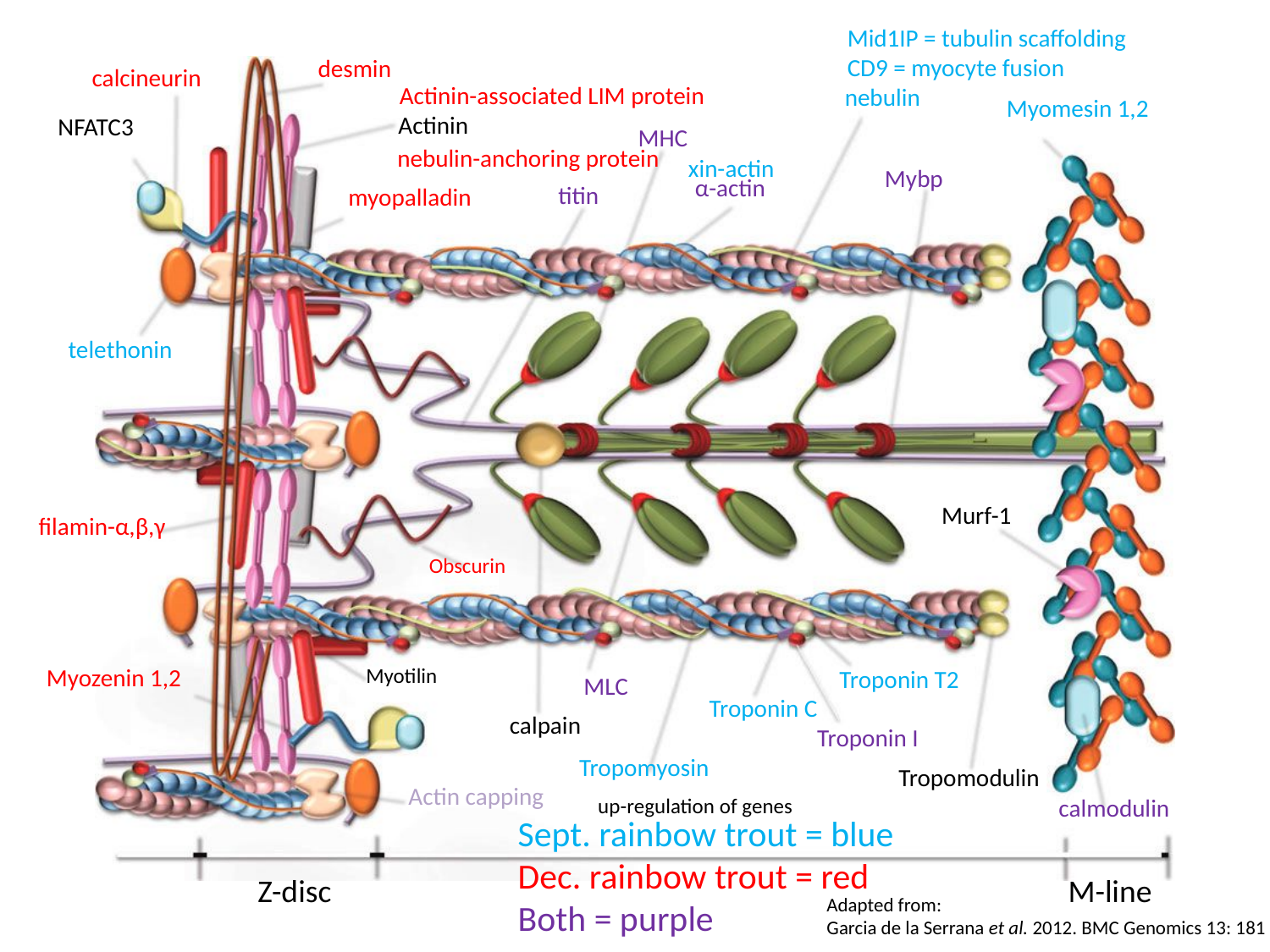

Mid1IP = tubulin scaffolding
CD9 = myocyte fusion
desmin
calcineurin
nebulin
Myomesin 1,2
Actinin
NFATC3
MHC
Mybp
α-actin
titin
myopalladin
telethonin
Murf-1
filamin-α,β,γ
Obscurin
Myozenin 1,2
Myotilin
Troponin T2
MLC
Troponin C
calpain
Troponin I
Tropomyosin
Tropomodulin
Actin capping
calmodulin
Z-disc
M-line
Actinin-associated LIM protein
nebulin-anchoring protein
xin-actin
up-regulation of genes
Sept. rainbow trout = blue
Dec. rainbow trout = red
Both = purple
Adapted from:
Garcia de la Serrana et al. 2012. BMC Genomics 13: 181
