# Supplementary material for: Transcriptome profiling in fast versus slow-growing rainbow trout across seasonal gradients
Source: BMC Genomics. 2016 Jan 15;17:60. doi: 10.1186/s12864-016-2363-5 (PMC4714434; doi:10.1186/s12864-016-2363-5)
Supplement: Additional file 14: — Results from the Broad Institute gene set enrichment analysis (GSEA) highlighting significant pathways and terms found in female fish. FDR significant categories from the Biological Process and Canonical, KEGG, BIOCARTA, and REACTOME pathway categories are shown. (PDF 707 kb) [file 12864_2016_2363_MOESM14_ESM.pdf]

Compute Overlaps for Selected Genes

Converted 156 submitted identifiers into 151 entrez genes. click here for details.

Collections	# Overlaps Shown	# Gene Sets in Collections	# Genes in Comparison (n)	# Genes in Universe (N)
BP, CP, CP:BIOCARTA, CP:KEGG, CP:REACTOME	81	2155	151	45956

Click the gene set name to see the gene set page. Click the number of genes [in brackets] to download the list of genes.

Color bar shading from light green to black, where lighter colors indicate more significant FDR q-values (< 0.05) and black indicates less significant FDR q-values (>= 0.05).

Save to: Excel | GenomeSpace

Gene Set Name [# Genes (K)]	Description	# Genes in Overlap (k)	k/K	p-value	FDR q-value
BIOPOLYMER_METABOLIC_PROCESS [1684]	Genes annotated by the GO term GO:0043283. The chemical reactions and pathways involving biopolymers, long, repeating chains of monomers found in nature e.g. polysaccharides and proteins.	26		5.27 e-11	1.14 e-7
NUCLEOBASENUCLEOSIDENUCLEOTIDE_AND_NUCNUCLEIC_ACID_METABOLIC_PROCESS [1244]	Genes annotated by the GO term GO:0006139. The chemical reactions and pathways involving nucleobases, nucleosides, nucleotides and nucleic acids.	20		5.6 e-9	6.04 e-6
RNA_METABOLIC_PROCESS [841]	Genes annotated by the GO term GO:0016070. The chemical reactions and pathways involving RNA, ribonucleic acid, one of the two main type of nucleic acid, consisting of a long, unbranched macromolecule formed from ribonucleotides joined in 3',5'-phosphodiester linkage.	16		2.12 e-8	1.53 e-5
PID_INTEGRIN4_PATHWAY [11]	Alpha6 beta4 integrin-ligand interactions	4		3.63 e-8	1.96 e-5
ANATOMICAL_STRUCTURE_DEVELOPMENT [1013]	Genes annotated by the GO term GO:0048856. The biological process whose specific outcome is the progression of an anatomical structure from an initial condition to its mature state. This process begins with the formation of the structure and ends with the mature structure, whatever form that may be including its natural destruction. An anatomical structure is any biological entity that occupies space and is distinguished from its surroundings. Anatomical structures can be macroscopic such as a carpel, or microscopic such as an acrosome.	17		4.61 e-8	1.99 e-5
ORGAN_DEVELOPMENT [571]	Genes annotated by the GO term GO:0048513.	13		6.17 e-8	2.22 e-5

	Development of a tissue or tissues that work together to perform a specific function or functions. Development pertains to the process whose specific outcome is the progression of a structure over time, from its formation to the mature structure. Organs are commonly observed as visibly distinct structures, but may also exist as loosely associated clusters of cells that work together to perform a specific function or functions.				
NABA_BASEMENT_MEMBRANES [40]	Genes encoding structural components of basement membranes	5		$2.15\ e^{-7}$	$6.62\ e^{-5}$
KEGG_ECM_RECEPTOR_INTERACTION [84]	ECM-receptor interaction	6		$3.75\ e^{-7}$	$8.89\ e^{-5}$
REGULATION_OF_CELLULAR_METABOLIC_PROCE OCESS [787]	Genes annotated by the GO term GO:0031323. Any process that modulates the frequency, rate or extent of the chemical reactions and pathways by which individual cells transform chemical substances.	14		$3.79\ e^{-7}$	$8.89\ e^{-5}$
REGULATION_OF_TRANSCRIPTION [566]	Genes annotated by the GO term GO:0045449. Any process that modulates the frequency, rate or extent of the synthesis of either RNA on a template of DNA or DNA on a template of RNA.	12		$4.31\ e^{-7}$	$8.89\ e^{-5}$
REGULATION_OF_METABOLIC_PROCESS [799]	Genes annotated by the GO term GO:0019222. Any process that modulates the frequency, rate or extent of the chemical reactions and pathways within a cell or an organism.	14		$4.54\ e^{-7}$	$8.89\ e^{-5}$
MUSCLE_DEVELOPMENT [93]	Genes annotated by the GO term GO:0007517. The process whose specific outcome is the progression of the muscle over time, from its formation to the mature structure. The muscle is an organ consisting of a tissue made up of various elongated cells that are specialized to contract and thus to produce movement and mechanical work.	6		$6.86\ e^{-7}$	$1.23\ e^{-4}$
REGULATION_OF_NUCLEOBASENUCLEOSIDENUCL UCLEOTIDE_AND_NUCLEIC_ACID_METABOLIC_P C_PROCESS [618]	Genes annotated by the GO term GO:0019219. Any process that modulates the frequency, rate or extent of the chemical reactions and pathways involving nucleobases, nucleosides, nucleotides and nucleic acids.	12		$1.08\ e^{-6}$	$1.68\ e^{-4}$
SYSTEM_DEVELOPMENT [861]	Genes annotated by the GO term GO:0048731. The process whose specific outcome is the progression of an organismal system over time, from its formation to the mature structure. A system is a regularly interacting or interdependent group of organs or tissues that work together to carry out a given biological	14		$1.09\ e^{-6}$	$1.68\ e^{-4}$

	process.				
TRANSCRIPTION [753]	Genes annotated by the GO term GO:0006350. The synthesis of either RNA on a template of DNA or DNA on a template of RNA.	13		$1.4 e^{-6}$	$1.91 e^{-4}$
TRANSCRIPTION_DNA_DEPENDENT [636]	Genes annotated by the GO term GO:0006351. The synthesis of RNA on a template of DNA.	12		$1.45 e^{-6}$	$1.91 e^{-4}$
RNA_BIOSYNTHETIC_PROCESS [638]	Genes annotated by the GO term GO:0032774. The chemical reactions and pathways resulting in the formation of RNA, ribonucleic acid, one of the two main type of nucleic acid, consisting of a long, unbranched macromolecule formed from ribonucleotides joined in 3',5'-phosphodiester linkage. Includes polymerization of ribonucleotide monomers.	12		$1.5 e^{-6}$	$1.91 e^{-4}$
MULTICELLULAR_ORGANISMAL_DEVELOPMENT [1049]	Genes annotated by the GO term GO:0007275. The biological process whose specific outcome is the progression of an organism over time from an initial condition (e.g. a zygote or a young adult) to a later condition (e.g. a multicellular animal or an aged adult).	15		$2.17 e^{-6}$	$2.6 e^{-4}$
TRANSPORT [795]	Genes annotated by the GO term GO:0006810. The directed movement of substances (such as macromolecules, small molecules, ions) into, out of, within or between cells.	13		$2.53 e^{-6}$	$2.8 e^{-4}$
REGULATION_OF_GENE_EXPRESSION [673]	Genes annotated by the GO term GO:0010468. Any process that modulates the frequency, rate or extent of gene expression. Gene expression is the process in which a gene's coding sequence is converted into a mature gene product or products (proteins or RNA). This includes the production of an RNA transcript as well as any processing to produce a mature RNA product or an mRNA (for protein-coding genes) and the translation of that mRNA into protein. Some protein processing events may be included when they are required to form an active form of a product from an inactive precursor form.	12		$2.6 e^{-6}$	$2.8 e^{-4}$
PID_INTEGRIN1_PATHWAY [66]	Beta1 integrin cell surface interactions	5		$2.72 e^{-6}$	$2.8 e^{-4}$
PROTEIN_METABOLIC_PROCESS [1231]	Genes annotated by the GO term GO:0019538. The chemical reactions and pathways involving a specific protein, rather than of proteins in general. Includes protein modification.	16		$3.32 e^{-6}$	$3.12 e^{-4}$
REGULATION_OF_TRANSCRIPTIONDNA_DEPENDENDENT [461]	Genes annotated by the GO term GO:0006355. Any process that modulates the frequency, rate or extent of DNA-dependent transcription.	10		$3.33 e^{-6}$	$3.12 e^{-4}$

REGULATION_OF_RNA_METABOLIC_PROCESS [471]	Genes annotated by the GO term GO:0051252. Any process that modulates the frequency, rate or extent of the chemical reactions and pathways involving RNA.	10		$4.02\ e^{-6}$	$3.61\ e^{-4}$
KEGG_FOCAL_ADHESION [201]	Focal adhesion	7		$5\ e^{-6}$	$4.31\ e^{-4}$
PID_ARF6_PATHWAY [35]	Arf6 signaling events	4		$5.42\ e^{-6}$	$4.49\ e^{-4}$
REACTOME_INTEGRIN_CELL_SURFACE_INTERACRACTIONS [79]	Genes involved in Integrin cell surface interactions	5		$6.64\ e^{-6}$	$5.15\ e^{-4}$
ESTABLISHMENT_OF_LOCALIZATION [870]	Genes annotated by the GO term GO:0051234. The directed movement of a cell, substance or cellular entity, such as a protein complex or organelle, to a specific location.	13		$6.69\ e^{-6}$	$5.15\ e^{-4}$
CELLULAR_COMPONENT_ASSEMBLY [298]	Genes annotated by the GO term GO:0022607. A cellular process that results in the assembly of a part of the cell.	8		$7.09\ e^{-6}$	$5.27\ e^{-4}$
KEGG_SMALL_CELL_LUNG_CANCER [84]	Small cell lung cancer	5		$8.97\ e^{-6}$	$6.45\ e^{-4}$
POSITIVE_REGULATION_OF_CELLULAR_PROCESCESS [668]	Genes annotated by the GO term GO:0048522. Any process that activates or increases the frequency, rate or extent of cellular processes, those that are carried out at the cellular level, but are not necessarily restricted to a single cell. For example, cell communication occurs among more than one cell, but occurs at the cellular level.	11		$1.44\ e^{-5}$	$1\ e^{-3}$
PID_A6B1_A6B4_INTEGRIN_PATHWAY [46]	a6b1 and a6b4 Integrin signaling	4		$1.64\ e^{-5}$	$1.11\ e^{-3}$
POSITIVE_REGULATION_OF_BIOLOGICAL_PROCROCESS [709]	Genes annotated by the GO term GO:0048518. Any process that activates or increases the frequency, rate or extent of a biological process. Biological processes are regulated by many means; examples include the control of gene expression, protein modification or interaction with a protein or substrate molecule.	11		$2.49\ e^{-5}$	$1.63\ e^{-3}$
PID_NFAT_3PATHWAY [54]	Role of Calcineurin-dependent NFAT signaling in lymphocytes	4		$3.12\ e^{-5}$	$1.98\ e^{-3}$
NABA_CORE_MATRISOME [275]	Ensemble of genes encoding core extracellular matrix including ECM glycoproteins, collagens and proteoglycans	7		$3.77\ e^{-5}$	$2.32\ e^{-3}$
MACROMOLECULAR_COMPLEX_ASSEMBLY [280]	Genes annotated by the GO term GO:0065003. The aggregation, arrangement and bonding together of a set of macromolecules to form a complex.	7		$4.22\ e^{-5}$	$2.53\ e^{-3}$
CYTOSKELETON_ORGANIZATION_AND_BIOGENESNESIS [208]	Genes annotated by the GO term GO:0007010. A process that is carried out at the cellular level which results in the formation, arrangement of constituent parts, or disassembly of cytoskeletal structures.	6		$6.9\ e^{-5}$	$4.02\ e^{-3}$
KEGG_VIRAL_MYOCARDITIS [73]	Viral myocarditis	4		$1.02\ e^{-4}$	$5.8\ e^{-3}$
KEGG_PATHWAYS_IN_CANCER [328]	Pathways in cancer	7		$1.13\ e^{-4}$	$6.27\ e^{-3}$

PID_TCRCALCIUMPATHWAY [29]	Calcium signaling in the CD4+ TCR pathway	3		$1.19\ e^{-4}$	$6.28\ e^{-3}$
POSITIVE_REGULATION_OF_TRANSCRIPTION [144]	Genes annotated by the GO term GO:0045941. Any process that activates or increases the frequency, rate or extent of transcription.	5		$1.19\ e^{-4}$	$6.28\ e^{-3}$
REACTOME_APOPTOSIS [148]	Genes involved in Apoptosis	5		$1.36\ e^{-4}$	$6.97\ e^{-3}$
TRANSCRIPTION_FROM_RNA_POLYMERASE_II_PI_PROMOTER [457]	Genes annotated by the GO term GO:0006366. The synthesis of RNA from a DNA template by RNA polymerase II (Pol II), originating at a Pol II-specific promoter. Includes transcription of messenger RNA (mRNA) and certain small nuclear RNAs (snRNAs).	8		$1.45\ e^{-4}$	$7.16\ e^{-3}$
SKELETAL_MUSCLE_DEVELOPMENT [31]	Genes annotated by the GO term GO:0007519. The developmental sequence of events leading to the formation of adult muscle that occurs in the anima. In vertebrate skeletal muscle the main events are: the fusion of myoblasts to form myotubes that increase in size by further fusion to them of myoblasts, the formation of myofibrils within their cytoplasm and the establishment of functional neuromuscular junctions with motor neurons. At this stage they can be regarded as mature muscle fibers.	3		$1.46\ e^{-4}$	$7.16\ e^{-3}$
POSITIVE_REGULATION_OF_NUCLEOBASENUCLEOSIDENUCLEOTIDE_AND_NUCLEIC_ACID_ME_METABOLIC_PROCESS [154]	Genes annotated by the GO term GO:0045935. Any process that activates or increases the frequency, rate or extent of the chemical reactions and pathways involving nucleobases, nucleosides, nucleotides and nucleic acids.	5		$1.63\ e^{-4}$	$7.83\ e^{-3}$
ORGANELLE_ORGANIZATION_AND_BIOGENESIS [473]	Genes annotated by the GO term GO:0006996. A process that is carried out at the cellular level which results in the formation, arrangement of constituent parts, or disassembly of any organelle within a cell.	8		$1.83\ e^{-4}$	$8.46\ e^{-3}$
KEGG_HYPERTROPHIC_CARDIOMYOPATHY_HCM [85]	Hypertrophic cardiomyopathy (HCM)	4		$1.84\ e^{-4}$	$8.46\ e^{-3}$
BIOCARTA_AGR_PATHWAY [36]	Agrin in Postsynaptic Differentiation	3		$2.29\ e^{-4}$	$1.03\ e^{-2}$
PROTEIN_COMPLEX_ASSEMBLY [167]	Genes annotated by the GO term GO:0006461. The aggregation, arrangement and bonding together of a set of components to form a protein complex.	5		$2.38\ e^{-4}$	$1.05\ e^{-2}$
KEGG_DILATED_CARDIOMYOPATHY [92]	Dilated cardiomyopathy	4		$2.5\ e^{-4}$	$1.08\ e^{-2}$
REACTOME_APOPTOTIC_CLEAVAGE_OF_CELLULAULAR_PROTEINS [40]	Genes involved in Apoptotic cleavage of cellular proteins	3		$3.14\ e^{-4}$	$1.3\ e^{-2}$
STRIATED_MUSCLE_DEVELOPMENT [40]	Genes annotated by the GO term GO:0014706. The process whose specific outcome is the progression of a striated muscle over time, from its formation to the mature structure. Striated muscle contain fibers that	3		$3.14\ e^{-4}$	$1.3\ e^{-2}$

	are divided by transverse bands into striations, and cardiac and skeletal muscle are types of striated muscle. Skeletal muscle myoblasts fuse to form myotubes and eventually multinucleated muscle fibers. The fusion of cardiac cells is very rare and can only form binucleate cells.				
CELLULAR_PROTEIN_METABOLIC_PROCESS [1117]	Genes annotated by the GO term GO:0044267. The chemical reactions and pathways involving a specific protein, rather than of proteins in general, occurring at the level of an individual cell. Includes protein modification.	12		3.42 e^{-4}	1.39 e^{-2}
ION_TRANSPORT [185]	Genes annotated by the GO term GO:0006811. The directed movement of charged atoms or small charged molecules into, out of, within or between cells.	5		3.81 e^{-4}	1.5 e^{-2}
CELLULAR_MACROMOLECULE_METABOLIC_PROCE OCESS [1131]	Genes annotated by the GO term GO:0044260. The chemical reactions and pathways involving macromolecules, large molecules including proteins, nucleic acids and carbohydrates, as carried out by individual cells.	12		3.82 e^{-4}	1.5 e^{-2}
PID_INTEGRIN3_PATHWAY [43]	Beta3 integrin cell surface interactions	3		3.9 e^{-4}	1.5 e^{-2}
ACTIN_CYTOSKELETON_ORGANIZATION_AND_BI _BIOGENESIS [105]	Genes annotated by the GO term GO:0030036. A process that is carried out at the cellular level which results in the formation, arrangement of constituent parts, or disassembly of cytoskeletal structures comprising actin filaments and their associated proteins.	4		4.14 e^{-4}	1.56 e^{-2}
POSITIVE_REGULATION_OF_EPITHELIAL_CELL ELL_PROLIFERATION [10]	Genes annotated by the GO term GO:0050679. Any process that activates or increases the rate or extent of epithelial cell proliferation.	2		4.74 e^{-4}	1.73 e^{-2}
REACTOME_SYNTHESIS_OF_PIPS_AT_THE_LATE ATE_ENDOSOME_MEMBRANE [10]	Genes involved in Synthesis of PIPs at the late endosome membrane	2		4.74 e^{-4}	1.73 e^{-2}
REACTOME_MUSCLE_CONTRACTION [48]	Genes involved in Muscle contraction	3		5.4 e^{-4}	1.94 e^{-2}
ACTIN_FILAMENT_BASED_PROCESS [115]	Genes annotated by the GO term GO:0030029. Any cellular process that depends upon or alters the actin cytoskeleton, that part of the cytoskeleton comprising actin filaments and their associated proteins.	4		5.83 e^{-4}	2.06 e^{-2}
METAL_ION_TRANSPORT [117]	Genes annotated by the GO term GO:0030001. The directed movement of metal ions, any metal ion with an electric charge, into, out of, within or between cells.	4		6.22 e^{-4}	2.16 e^{-2}
REACTOME_SYNTHESIS_OF_PIPS_AT_THE_EARL ARLY_ENDOSOME_MEMBRANE [12]	Genes involved in Synthesis of PIPs at the early endosome membrane	2		6.93 e^{-4}	2.37 e^{-2}
REACTOME_APOPTOTIC_EXECUTION_PHASE [54]	Genes involved in Apoptotic execution phase	3		7.63 e^{-4}	2.57 e^{-2}

REACTOME_CASPASE_MEDIATED_CLEAVAGE_OF_OF_CYTOSKELETAL_PROTEINS [13]	Genes involved in Caspase-mediated cleavage of cytoskeletal proteins	2		8.17 e^{-4}	2.71 e^{-2}
STRIATED_MUSCLE_CONTRACTION_GO_0006941 [14]	Genes annotated by the GO term GO:0006941. A process whereby force is generated within striated muscle tissue, resulting in a change in muscle geometry. Force generation involves a chemo-mechanical energy conversion step. The chemo-mechanical energy conversion step is carried out by the actin/myosin complex activity, which generates force through ATP hydrolysis. Striated muscle is a type of muscle in which the repeating units (sarcomeres) of the contractile myofibrils are arranged in registry throughout the cell, resulting in transverse or oblique striations observable at the level of the light microscope.	2		9.51 e^{-4}	3.11 e^{-2}
POSITIVE_REGULATION_OF_CELLULAR_METABOLIC_PROCESS [229]	Genes annotated by the GO term GO:0031325. Any process that activates or increases the frequency, rate or extent of the chemical reactions and pathways by which individual cells transform chemical substances.	5		9.98 e^{-4}	3.21 e^{-2}
POST_TRANSLATIONAL_PROTEIN_MODIFICATION [476]	Genes annotated by the GO term GO:0043687. The covalent alteration of one or more amino acids occurring in a protein after the protein has been completely translated and released from the ribosome.	7		1.05 e^{-3}	3.33 e^{-2}
PID_AR_PATHWAY [61]	Coregulation of Androgen receptor activity	3		1.09 e^{-3}	3.37 e^{-2}
CYTOPLASM_ORGANIZATION_AND_BIOGENESIS [15]	Genes annotated by the GO term GO:0007028. A process that is carried out at the cellular level which results in the formation, arrangement of constituent parts, or disassembly of the cytoplasm and its components.	2		1.09 e^{-3}	3.37 e^{-2}
POSITIVE_REGULATION_OF_METABOLIC_PROCEOCESS [236]	Genes annotated by the GO term GO:0009893. Any process that activates or increases the frequency, rate or extent of the chemical reactions and pathways within a cell or an organism.	5		1.14 e^{-3}	3.45 e^{-2}
KEGG_UBIQUITIN_MEDIATED_PROTEOLYSIS [138]	Ubiquitin mediated proteolysis	4		1.15 e^{-3}	3.45 e^{-2}
PROTEIN_MODIFICATION_PROCESS [631]	Genes annotated by the GO term GO:0006464. The covalent alteration of one or more amino acids occurring in proteins, peptides and nascent polypeptides (co-translational, post-translational modifications). Includes the modification of charged tRNAs that are destined to occur in a protein (pre-translation modification).	8		1.21 e^{-3}	3.56 e^{-2}
CELL_MATURATION [16]	Genes annotated by the	2		1.25 e^{-3}	3.64 e^{-2}

	GO term GO:0048469. A developmental process, independent of morphogenetic (shape) change, that is required for a cell to attain its fully functional state.				
MYOBLAST_DIFFERENTIATION [17]	Genes annotated by the GO term GO:0045445. The process whereby a relatively unspecialized cell acquires specialized features of a myoblast. A myoblast is a mononucleate cell type that, by fusion with other myoblasts, gives rise to the myotubes that eventually develop into skeletal muscle fibers.	2		$1.41\ e^{-3}$	$4.06\ e^{-2}$
CATION_TRANSPORT [147]	Genes annotated by the GO term GO:0006812. The directed movement of cations, atoms or small molecules with a net positive charge, into, out of, within or between cells.	4		$1.45\ e^{-3}$	$4.07\ e^{-2}$
BIOPOLYMER_MODIFICATION [650]	Genes annotated by the GO term GO:0043412. The covalent alteration of one or more monomeric units in a polypeptide, polynucleotide, polysaccharide, or other biological polymer, resulting in a change in its properties.	8		$1.45\ e^{-3}$	$4.07\ e^{-2}$
REACTOME_AXON_GUIDANCE [251]	Genes involved in Axon guidance	5		$1.5\ e^{-3}$	$4.13\ e^{-2}$
DEVELOPMENTAL_MATURATION [18]	Genes annotated by the GO term GO:0021700. A developmental process, independent of morphogenetic (shape) change, that is required for an anatomical structure, cell or cellular component to attain its fully functional state.	2		$1.59\ e^{-3}$	$4.28\ e^{-2}$
ANATOMICAL_STRUCTURE_MORPHOGENESIS [376]	Genes annotated by the GO term GO:0009653. The process by which anatomical structures are generated and organized. Morphogenesis pertains to the creation of form.	6		$1.59\ e^{-3}$	$4.28\ e^{-2}$
INTRACELLULAR_SIGNALING_CASCADE [667]	Genes annotated by the GO term GO:0007242. A series of reactions within the cell that occur as a result of a single trigger reaction or compound.	8		$1.71\ e^{-3}$	$4.55\ e^{-2}$

Gene/geneset overlap matrix

Entrez Gene Id	Gene Symbol	BIOPOLYMER_METABOLIC_PROCESS	NUCLEOBASENUCLEOSIDENUCLEOTIDE_AND_NUCLEIC_ACID_METABOLIC_PROCESS	RNA_METABOLIC_PROCESS	PID_INTEGRIN4_PATHWAY	ANATOMICAL_STRUCTURE_DEVELOPMENT	ORGAN_DEVELOPMENT	NABA_BASEMENT_MEMBRANES	KEGG_ECM_RECEPTOR_INTERACTION	REGULATION_OF_CELLULAR_METABOLIC_PROCESS	REGULATION_OF_TRANSCRIPTION	REGULATION_OF_METABOLIC_PROCESS	MUSCLE_DEVELOPMENT	REGULATION_OF_NUCLEOBASENUCLEOSIDENUCLEOTIDE_AND_NUCLEIC_ACID_METABOLIC_PROCESS	SYSTEM_DEVELOPMENT	TRANSCRIPTION	TRANSCRIPTION_DNA_DEPENDENT	RNA_BIOSYNTHETIC_PROCESS	MULTICELLULAR_ORGANISMAL_DEVELOPMENT	TRANSPORT	REGULATION_OF_GENE_EXPRESSION	PID_INTEGRIN1_PATHWAY	PROTEIN_METABOLIC_PROCESS	REGULATION_OF_TRANSCRIPTIONDNA_DEPENDENT	REGULATION_OF_RNA_METABOLIC_PROCESS	KEGG_FOCAL_ADHESION	PID_ARF6_PATHWAY	REACTOME_INTEGRIN_CELL_SURFACE_INTERACTIONS
6096	RORB	■	■	■		■	■			■	■	■		■	■	■	■	■	■		■			■	■			
4853	NOTCH2	■	■	■		■	■			■	■	■		■	■	■	■	■	■		■			■	■			
6939	TCF15	■	■	■		■	■			■	■	■		■	■	■	■	■	■		■			■	■			
4297	MLL	■	■	■		■	■								■	■	■	■	■				■					
6239	RREB1	■	■	■						■	■	■		■		■	■	■	■		■			■	■			
22906	TRAK1	■	■	■						■	■	■		■		■	■	■		■	■		■	■	■			
9149	DYRK1B	■	■	■						■	■	■		■		■	■	■			■		■	■	■			
9969	MED13	■	■	■						■	■	■		■		■	■	■			■			■	■			
4775	NFATC3	■	■	■						■	■	■		■		■	■	■			■			■	■			
10847	SRCAP	■	■	■						■	■	■		■		■	■	■			■			■	■			
1112	FOXN3	■	■	■						■	■	■		■		■	■	■			■			■	■			
5927	KDM5A	■	■	■						■	■	■		■		■	■	■			■							
23381	SMG5	■	■	■						■		■								■								
7536	SF1	■	■	■																								
6421	SFPQ	■	■	■																								
6625	SNRNP70	■	■	■																								
4595	MUTYH	■	■																									

Gene Symbol	ESTABLISHMENT_OF_LOCALIZATION	CELLULAR_COMPONENT_ASSEMBLY	KEGG_SMALL_CELL_LUNG_CANCER	POSITIVE_REGULATION_OF_CELLULAR_PROCESS	PID_A6B1_A6B4_INTEGRIN_PATHWAY	POSITIVE_REGULATION_OF_BIOLOGICAL_PROCESS	PID_NFAT_3PATHWAY	NABA_CORE_MATRISOME	MACROMOLECULAR_COMPLEX_ASSEMBLY	CYTOSKELETON_ORGANIZATION_AND_BIOGENESIS	KEGG_VIRAL_MYOCARDITIS	KEGG_PATHWAYS_IN_CANCER	PID_TCRCALCIUMPATHWAY	POSITIVE_REGULATION_OF_TRANSCRIPTION	REACTOME_APOPTOSIS	TRANSCRIPTION_FROM_RNA_POLYMERASE_II_PROMOTER	SKELETAL_MUSCLE_DEVELOPMENT	POSITIVE_REGULATION_OF_NUCLEOBASENUCLEOSIDENUCLEOTIDE_AND_NUCLEIC_ACID_METABOLIC_PROCESS	ORGANELLE_ORGANIZATION_AND_BIOGENESIS	KEGG_HYPERTROPHIC_CARDIOMYOPATHY_HCM	BIOCARTA_AGR_PATHWAY	PROTEIN_COMPLEX_ASSEMBLY	KEGG_DILATED_CARDIOMYOPATHY	REACTOME_APOPTOTIC_CLEAVAGE_OF_CELLULAR_PROTEINS	STRIATED_MUSCLE_DEVELOPMENT	CELLULAR_PROTEIN_METABOLIC_PROCESS	ION_TRANSPORT
RORB				■		■								■				■									
NOTCH2				■		■																					
TCF15																■											
MLL		■							■							■						■					
RREB1																■											
TRAK1	■															■										■	
DYRK1B				■		■								■				■								■	
MED13		■		■		■			■					■		■		■									
NFATC3							■						■			■											
SRCAP																■											
FOXN3																											
KDM5A				■		■								■		■		■									
SMG5	■																										
SF1		■							■																		
SFPQ																											
SNRNP70																											
MUTYH																											

Gene Symbol	CELLULAR_MACROMOLECULE_METABOLIC_PROCESS	PID_INTEGRIN3_PATHWAY	ACTIN_CYTOSKELETON_ORGANIZATION_AND_BIOGENESIS	POSITIVE_REGULATION_OF_EPITHELIAL_CELL_PROLIFERATION	REACTOME_SYNTHESIS_OF_PIPS_AT_THE_LATE_ENDOSOME_MEMBRANE	REACTOME_MUSCLE_CONTRACTION	ACTIN_FILAMENT_BASED_PROCESS	METAL_ION_TRANSPORT	REACTOME_SYNTHESIS_OF_PIPS_AT_THE_EARLY_ENDOSOME_MEMBRANE	REACTOME_APOPTOTIC_EXECUTION_PHASE	REACTOME_CASPASE_MEDIATED_CLEAVAGE_OF_CYTOSKELETAL_PROTEINS	STRIATED_MUSCLE_CONTRACTION_GO_0006941	POSITIVE_REGULATION_OF_CELLULAR_METABOLIC_PROCESS	POST_TRANSLATIONAL_PROTEIN_MODIFICATION	PID_AR_PATHWAY	CYTOPLASM_ORGANIZATION_AND_BIOGENESIS	POSITIVE_REGULATION_OF_METABOLIC_PROCESS	KEGG_UBIQUITIN_MEDIATED_PROTEOLYSIS	PROTEIN_MODIFICATION_PROCESS	CELL_MATURATION	MYOBLAST_DIFFERENTIATION	CATION_TRANSPORT	BIOPOLYMER_MODIFICATION	REACTOME_AXON_GUIDANCE	DEVELOPMENTAL_MATURATION	ANATOMICAL_STRUCTURE_MORPHOGENESIS	INTRACELLULAR_SIGNALING_CASCADE	Entrez	Source	Gene Description
RORB													■				■									■			S	RAR-related orphan receptor B
NOTCH2																										■	■		S	notch 2
TCF15																													S	transcription factor 15 (basic helix-loop-helix)
MLL																													S	myeloid/lymphoid or mixed-lineage leukemia (trithorax homolog, Drosophila)
RREB1																											■		S	ras responsive element binding protein 1
TRAK1	■																		■				■						S	trafficking protein, kinesin binding 1
DYRK1B	■												■	■			■		■				■						S	dual-specificity tyrosine-(Y)-phosphorylation regulated kinase 1B
MED13													■				■										■		S	mediator complex subunit 13
NFATC3																													S	nuclear factor of activated T-cells, cytoplasmic, calcineurin-dependent 3
SRCAP																													S	Snf2-related CREBBP activator protein
FOXN3																											■		S	forkhead box N3
KDM5A													■				■												S	lysine (K)-specific demethylase 5A
SMG5																													S	smg-5 homolog, nonsense mediated mRNA decay factor (C. elegans)
SF1																													S	splicing factor 1
SFPQ																													S	splicing factor proline/glutamine-rich
SNRNP70																													S	small nuclear ribonucleoprotein 70kDa (U1)
MUTYH																													S	mutY homolog (E.

			coli)
5886	RAD23A	S	RAD23 homolog A (S. cerevisiae)
7273	TTN	S	titin
9578	CDC42BPB	S	CDC42 binding protein kinase beta (DMPK-like)
65125	WNK1	S	WNK lysine deficient protein kinase 1
10075	HUWE1	S	HECT, UBA and WWE domain containing 1
79813	EHMT1	S	euchromatic histone-lysine N-methyltransferase 1
10114	HIPK3	S	homeodomain interacting protein kinase 3
7326	UBE2G1	S	ubiquitin-conjugating enzyme E2G 1
23392	KIAA0368	S	KIAA0368
9915	ARNT2	S	aryl-hydrocarbon receptor nuclear translocator 2
2030	SLC29A1	S	solute carrier family 29 (nucleoside transporters), member 1
3908	LAMA2	S	laminin, alpha 2
3915	LAMC1	S	laminin, gamma 1 (formerly LAMB2)
3912	LAMB1	S	laminin, beta 1
3913	LAMB2	S	laminin, beta 2 (laminin S)
1277	COL1A1	S	collagen, type I, alpha 1
4629	MYH11	S	myosin, heavy chain 11, smooth muscle
4209	MEF2D	S	myocyte enhancer factor 2D
6840	SVIL	S	supervillin
10290	SPEG	S	SPEG complex locus
23580	CDC42EP4	S	CDC42 effector protein (Rho GTPase binding) 4
4653	MYOC	S	myocilin, trabecular meshwork inducible glucocorticoid response
3339	HSPG2	S	heparan sulfate proteoglycan 2
2934	GSN	S	gelsolin
23303	KIF13B	S	kinesin family member 13B
367	AR	S	androgen receptor
6261	RYR1	S	ryanodine receptor 1 (skeletal)
6263	RYR3	S	ryanodine receptor 3
538	ATP7A	S	ATPase, Cu++ transporting, alpha polypeptide
6330	SCN4B	S	sodium channel, voltage-gated, type IV, beta
7249	TSC2	S	tuberous sclerosis 2
26088	GGA1	S	golgi-associated, gamma adaptin ear containing, ARF binding protein 1
10749	KIF1C	S	kinesin family member 1C
5829	PXN	S	paxillin

7082	TJP1	S	tight junction protein 1 (zona occludens 1)
2318	FLNC	S	filamin C, gamma
9922	IQSEC1	S	IQ motif and Sec7 domain 1
9098	USP6	S	ubiquitin specific peptidase 6 (Tre-2 oncogene)
8517	IKBKG	S	inhibitor of kappa light polypeptide gene enhancer in B-cells, kinase gamma
142678	MIB2	S	mindbomb homolog 2 (Drosophila)
5534	PPP3R1	S	protein phosphatase 3, regulatory subunit B, alpha
23523	CABIN1	S	calcineurin binding protein 1
340267	COL28A1	S	collagen, type XXVIII, alpha 1
1756	DMD	S	dystrophin
4619	MYH1	S	myosin, heavy chain 1, skeletal muscle, adult
5339	PLEC	S	plectin
5716	PSMD10	S	proteasome (prosome, macropain) 26S subunit, non-ATPase, 10
782	CACNB1	S	calcium channel, voltage-dependent, beta 1 subunit
30849	PIK3R4	S	phosphoinositide-3-kinase, regulatory subunit 4
9110	MTMR4	S	myotubularin related protein 4
4703	NEB	S	nebulin
8736	MYOM1	S	myomesin 1, 185kDa
9320	TRIP12	S	thyroid hormone receptor interactor 12
8925	HERC1	S	hect (homologous to the E6-AP (UBE3A) carboxyl terminus) domain and RCC1 (CHC1)-like domain (RLD) 1
2262	GPC5	S	glypican 5
947	CD34	S	CD34 molecule
977	CD151	S	CD151 molecule (Raph blood group)
4359	MPZ	S	myelin protein zero
10659	CELF2	S	CUGBP, Elav-like family member 2
1180	CLCN1	S	chloride channel 1, skeletal muscle
57690	TNRC6C	S	trinucleotide repeat containing 6C
100507436	MICA	S	MHC class I polypeptide-related sequence A
7145	TNS1	S	tensin 1
1738	DLD	S	dihydrolipoamide dehydrogenase
32	ACACB	S	acetyl-CoA carboxylase beta
10919	EHMT2	S	euchromatic histone-lysine N-methyltransferase 2

847	CAT	S	catalase
6168	RPL37A	S	ribosomal protein L37a
4849	CNOT3	S	CCR4-NOT transcription complex, subunit 3
8239	USP9X	S	ubiquitin specific peptidase 9, X-linked
678	ZFP36L2	S	zinc finger protein 36, C3H type-like 2
7037	TFRC	S	transferrin receptor (p90, CD71)
54453	RIN2	S	Ras and Rab interactor 2
58513	EPS15L1	S	epidermal growth factor receptor pathway substrate 15-like 1
57187	THOC2	S	THO complex 2
25946	ZNF385A	S	zinc finger protein 385A
140453	MUC17	S	mucin 17, cell surface associated
84033	OBSCN	S	obscurin, cytoskeletal calmodulin and titin-interacting RhoGEF
10730	YME1L1	S	YME1-like 1 (S. cerevisiae)
388698	FLG2	S	filaggrin family member 2
54778	RNF111	S	ring finger protein 111
4122	MAN2A2	S	mannosidase, alpha, class 2A, member 2
11156	PTP4A3	S	protein tyrosine phosphatase type IVA, member 3
10129	FRY	S	furry homolog (Drosophila)
10365	KLF2	S	Kruppel-like factor 2 (lung)
11273	ATXN2L	S	ataxin 2-like
120	ADD3	S	adducin 3 (gamma)
140890	SREK1	S	splicing regulatory glutamine/lysine-rich protein 1
171024	SYNPO2	S	synaptopodin 2
221937	FOXK1	S	forkhead box K1
22828	SCAF8	S	SR-related CTD-associated factor 8
22864	R3HDM2	S	R3H domain containing 2
23022	PALLD	S	palladin, cytoskeletal associated protein
23031	MAST3	S	microtubule associated serine/threonine kinase 3
23066	CAND2	S	cullin-associated and neddylation-dissociated 2 (putative)
23130	ATG2A	S	ATG2 autophagy related 2 homolog A (S. cerevisiae)
23195	MDN1	S	MDN1, midasin homolog (yeast)
23211	ZC3H4	S	zinc finger CCCH-type containing 4
23215	PRRC2C	S	proline-rich coiled-coil 2C
23230	VPS13A	S	vacuolar protein sorting 13 homolog A (S.

			cerevisiae)
23499	MACF1	S	microtubule-actin crosslinking factor 1
254102	EHBP1L1	S	EH domain binding protein 1-like 1
25938	HEATR5A	S	HEAT repeat containing 5A
26053	AUTS2	S	autism susceptibility candidate 2
26057	ANKRD17	S	ankyrin repeat domain 17
282996	RBM20	S	RNA binding motif protein 20
375775	PNPLA7	S	patatin-like phospholipase domain containing 7
377007	KLHL30	S	kelch-like 30 (Drosophila)
401265	KLHL31	S	kelch-like 31 (Drosophila)
4134	MAP4	S	microtubule-associated protein 4
4892	NRAP	S	nebulin-related anchoring protein
50626	CYHR1	S	cysteine/histidine-rich 1
54882	ANKHD1	S	ankyrin repeat and KH domain containing 1
55075	UACA	S	uveal autoantigen with coiled-coil domains and ankyrin repeats
55324	ABCF3	S	ATP-binding cassette, sub-family F (GCN20), member 3
55553	SOX6	S	SRY (sex determining region Y)-box 6
55719	FAM178A	S	family with sequence similarity 178, member A
55833	UBAP2	S	ubiquitin associated protein 2
56658	TRIM39	S	tripartite motif containing 39
56704	JPH1	S	junctophilin 1
56946	C11orf30	S	chromosome 11 open reading frame 30
57182	ANKRD50	S	ankyrin repeat domain 50
57488	ESYT2	S	extended synaptotagmin-like protein 2
57571	CARNS1	S	carnosine synthase 1
57606	SLAIN2	S	SLAIN motif family, member 2
57643	ZSWIM5	S	zinc finger, SWIM-type containing 5
80315	CPEB4	S	cytoplasmic polyadenylation element binding protein 4
84665	MYPN	S	myopalladin
84726	PRRC2B	S	proline-rich coiled-coil 2B
9144	SYNGR2	S	synaptogyrin 2
9229	DLGAP1	S	discs, large (Drosophila) homolog-associated protein 1
9659	PDE4DIP	S	phosphodiesterase 4D interacting protein

9710	KIAA0355																																																																																			S	KIAA0355
9885	OSBPL2																																																																																				S	oxysterol binding protein-like 2
