# Supplementary material for: Transcriptome profiling in fast versus slow-growing rainbow trout across seasonal gradients
Source: BMC Genomics. 2016 Jan 15;17:60. doi: 10.1186/s12864-016-2363-5 (PMC4714434; doi:10.1186/s12864-016-2363-5)
Supplement: Additional file 15: — Results from the Broad Institute gene set enrichment analysis (GSEA) highlighting significant pathways and terms found in male fish. FDR significant categories from the Biological Process and Canonical, KEGG, BIOCARTA, and REACTOME pathway categories are shown. (PDF 528 kb) [file 12864_2016_2363_MOESM15_ESM.pdf]

logged in as rdanzman@uoguelph.ca
logout
BROAD INSTITUTE
GSEA Gene Set Enrichment Analysis
GSEA Home Downloads Molecular Signatures Database Documentation Contact

- MSigDB Home
- About Collections
- Browse Gene Sets
- Search Gene Sets
- Investigate Gene Sets
- View Gene Families
- Help

Compute Overlaps for Selected Genes

Converted 115 submitted identifiers into 104 entrez genes. click here for details.

Collections	# Overlaps Shown	# Gene Sets in Collections	# Genes in Comparison (n)	# Genes in Universe (N)
BP, CP, CP:BIOCARTA, CP:KEGG, CP:REACTOME	93	2155	104	45956

Click the gene set name to see the gene set page. Click the number of genes [in brackets] to download the list of genes.

Color bar shading from light green to black, where lighter colors indicate more significant FDR q-values (< 0.05) and black indicates less significant FDR q-values (>= 0.05).

Save to: Excel | GenomeSpace

Gene Set Name [# Genes (K)]	Description	# Genes in Overlap (k)	k/K	p-value	FDR q-value
KEGG_RIBOSOME [88]	Ribosome	20		4.83 e-35	1.04 e-31
REACTOME_3_UTR_MEDIATED_TRANSLATIONAL_AL_REGULATION [176]	Genes involved in 3' -UTR-mediated translational regulation	21		2 e-30	2.15 e-27
REACTOME_TRANSLATION [222]	Genes involved in Translation	22		5.24 e-30	3.77 e-27
REACTOME_PEPTIDE_CHAIN_ELONGATION [153]	Genes involved in Peptide chain elongation	20		7.77 e-30	4.19 e-27
REACTOME_INFLUENZA_VIRAL_RNA_TRANSCRIPRIPTION_AND_REPLICATION [169]	Genes involved in Influenza Viral RNA Transcription and Replication	20		6.28 e-29	2.71 e-26
REACTOME_NONSENSE_MEDIATED_DECAY_ENHANHANCED_BY_THE_EXON_JUNCTION_COMPLEX [176]	Genes involved in Nonsense Mediated Decay Enhanced by the Exon Junction Complex	20		1.47 e-28	5.26 e-26
REACTOME_SRP_DEPENDENT_COTRANSLATIONALNAL_PROTEIN_TARGETING_TO_MEMBRANE [179]	Genes involved in SRP-dependent cotranslational protein targeting to membrane	20		2.08 e-28	6.42 e-26
REACTOME_INFLUENZA_LIFE_CYCLE [203]	Genes involved in Influenza Life Cycle	20		2.83 e-27	7.63 e-25
REACTOME_METABOLISM_OF_MRNA [284]	Genes involved in Metabolism of mRNA	21		6.22 e-26	1.49 e-23
REACTOME_METABOLISM_OF_RNA [330]	Genes involved in Metabolism of RNA	21		1.5 e-24	3.23 e-22
REACTOME_METABOLISM_OF_PROTEINS [518]	Genes involved in Metabolism of proteins	23		2.85 e-23	5.58 e-21
TRANSLATION [180]	Genes annotated by the GO term GO:0006412. The chemical reactions and pathways resulting in the formation of a protein. This is a ribosome-mediated process in which the information in messenger RNA (mRNA) is used to specify the sequence of amino acids in the protein.	14		7 e-18	1.26 e-15
CELLULAR_BIOSYNTHETIC_PROCESS [321]	Genes annotated by the GO term GO:0044249. The chemical reactions and pathways resulting in the formation of substances, carried out by individual cells.	16		3.37 e-17	5.58 e-15
BIOSYNTHETIC_PROCESS [470]	Genes annotated by the GO term GO:0009058. The energy-requiring	16		1.29 e-14	1.99 e-12

	part of metabolism in which simpler substances are transformed into more complex ones, as in growth and other biosynthetic processes.				
MACROMOLECULE_BIOSYNTHETIC_PROCESS [321]	Genes annotated by the GO term GO:0009059. The chemical reactions and pathways resulting in the formation of macromolecules, large molecules including proteins, nucleic acids and carbohydrates.	14		$2.24\ e^{-14}$	$3.22\ e^{-12}$
REACTOME_FORMATION_OF_THE_TERNARY_COMPLEX_AND_SUBSEQUENTLY_THE_43S_COMPLEX [74]	Genes involved in Formation of the ternary complex, and subsequently, the 43S complex	8		$6.96\ e^{-12}$	$9.38\ e^{-10}$
REACTOME_ACTIVATION_OF_THE_MRNA_UPON_BINDING_OF_THE_CAP_BINDING_COMPLEX_AND_EIFS_AND_SUBSEQUENT_BINDING_TO_43S [84]	Genes involved in Activation of the mRNA upon binding of the cap-binding complex and eIFs, and subsequent binding to 43S	8		$1.98\ e^{-11}$	$2.51\ e^{-9}$
CELLULAR_PROTEIN_METABOLIC_PROCESS [1117]	Genes annotated by the GO term GO:0044267. The chemical reactions and pathways involving a specific protein, rather than of proteins in general, occurring at the level of an individual cell. Includes protein modification.	18		$7.11\ e^{-11}$	$8.52\ e^{-9}$
CELLULAR_MACROMOLECULE_METABOLIC_PROCEOCESS [1131]	Genes annotated by the GO term GO:0044260. The chemical reactions and pathways involving macromolecules, large molecules including proteins, nucleic acids and carbohydrates, as carried out by individual cells.	18		$8.69\ e^{-11}$	$9.86\ e^{-9}$
PROTEIN_METABOLIC_PROCESS [1231]	Genes annotated by the GO term GO:0019538. The chemical reactions and pathways involving a specific protein, rather than of proteins in general. Includes protein modification.	18		$3.37\ e^{-10}$	$3.63\ e^{-8}$
KEGG_ALZHEIMERS_DISEASE [169]	Alzheimer's disease	7		$1.26\ e^{-7}$	$1.3\ e^{-5}$
REACTOME_GLYCOLYSIS [29]	Genes involved in Glycolysis	4		$5.63\ e^{-7}$	$5.51\ e^{-5}$
REACTOME_GLUCONEOGENESIS [34]	Genes involved in Gluconeogenesis	4		$1.09\ e^{-6}$	$1.02\ e^{-4}$
BIOCARTA_GLYCOLYSIS_PATHWAY [10]	Glycolysis Pathway	3		$1.34\ e^{-6}$	$1.2\ e^{-4}$
REACTOME_RESPIRATORY_ELECTRON_TRANSPORT_ATP_SYNTHESIS_BY_CHEMIOSMOTIC_COUPLING_AND_HEAT_PRODUCTION_BY_UNCOUPLING_PROTEINS_ [98]	Genes involved in Respiratory electron transport, ATP synthesis by chemiosmotic coupling, and heat production by uncoupling proteins.	5		$3.09\ e^{-6}$	$2.67\ e^{-4}$
STRIATED_MUSCLE_CONTRACTION_GO_0006941 [14]	Genes annotated by the GO term GO:0006941. A process whereby force is generated within striated muscle tissue, resulting in a change in muscle geometry. Force generation involves a chemo-mechanical energy conversion step. The chemo-mechanical energy conversion step is carried out by the actin/myosin complex activity, which generates force through	3		$4.02\ e^{-6}$	$3.34\ e^{-4}$

	ATP hydrolysis. Striated muscle is a type of muscle in which the repeating units (sarcomeres) of the contractile myofibrils are arranged in registry throughout the cell, resulting in transverse or oblique striations observable at the level of the light microscope.				
BIOCARTA_MTA3_PATHWAY [19]	Downregulated of MTA-3 in ER-negative Breast Tumors	3		$1.06\ e^{-5}$	$8.18\ e^{-4}$
REGULATION_OF_MUSCLE_CONTRACTION [19]	Genes annotated by the GO term GO:0006937. Any process that modulates the frequency, rate or extent of muscle contraction.	3		$1.06\ e^{-5}$	$8.18\ e^{-4}$
KEGG_GLYCOLYSIS_GLUCONEOGENESIS [62]	Glycolysis / Gluconeogenesis	4		$1.25\ e^{-5}$	$9.27\ e^{-4}$
KEGG_PARKINSONS_DISEASE [133]	Parkinson's disease	5		$1.38\ e^{-5}$	$9.88\ e^{-4}$
KEGG_OXIDATIVE_PHOSPHORYLATION [135]	Oxidative phosphorylation	5		$1.48\ e^{-5}$	$1.03\ e^{-3}$
REACTOME_TCA_CYCLE_AND_RESPIRATORY_ELECTRON_TRANSPORT [141]	Genes involved in The citric acid (TCA) cycle and respiratory electron transport	5		$1.82\ e^{-5}$	$1.21\ e^{-3}$
KEGG_COMPLEMENT_AND_COAGULATION_CASCADES [69]	Complement and coagulation cascades	4		$1.91\ e^{-5}$	$1.21\ e^{-3}$
REACTOME_GLUCOSE_METABOLISM [69]	Genes involved in Glucose metabolism	4		$1.91\ e^{-5}$	$1.21\ e^{-3}$
REACTOME_METABOLISM_OF_NUCLEOTIDES [72]	Genes involved in Metabolism of nucleotides	4		$2.26\ e^{-5}$	$1.39\ e^{-3}$
REACTOME_STRIATED_MUSCLE_CONTRACTION [27]	Genes involved in Striated Muscle Contraction	3		$3.17\ e^{-5}$	$1.89\ e^{-3}$
PID_MYC_ACTIVPATHWAY [79]	Validated targets of C-MYC transcriptional activation	4		$3.26\ e^{-5}$	$1.9\ e^{-3}$
KEGG_CARDIAC_MUSCLE_CONTRACTION [80]	Cardiac muscle contraction	4		$3.43\ e^{-5}$	$1.94\ e^{-3}$
REACTOME_LIPOPROTEIN_METABOLISM [28]	Genes involved in Lipoprotein metabolism	3		$3.54\ e^{-5}$	$1.96\ e^{-3}$
SYSTEM_PROCESS [563]	Genes annotated by the GO term GO:0003008. A biological process, occurring at the level of an organ system pertinent to the function of the organism. An organ system is a regularly interacting or interdependent group of organs or tissues that work together to carry out a given biological process.	8		$4.44\ e^{-5}$	$2.39\ e^{-3}$
KEGG_FRUCTOSE_AND_MANNOSE_METABOLISM [34]	Fructose and mannose metabolism	3		$6.4\ e^{-5}$	$3.36\ e^{-3}$
KEGG_HUNTINGTONS_DISEASE [185]	Huntington's disease	5		$6.67\ e^{-5}$	$3.42\ e^{-3}$
REACTOME_HEMOSTASIS [466]	Genes involved in Hemostasis	7		$9.61\ e^{-5}$	$4.81\ e^{-3}$
ANATOMICAL_STRUCTURE_DEVELOPMENT [1013]	Genes annotated by the GO term GO:0048856. The biological process whose specific outcome is the progression of an anatomical structure from an initial condition to its mature state. This process begins with the formation of the structure and ends with the mature structure, whatever form that may be including its natural destruction. An anatomical structure is	10		$1.04\ e^{-4}$	$5.11\ e^{-3}$

	any biological entity that occupies space and is distinguished from its surroundings. Anatomical structures can be macroscopic such as a carpel, or microscopic such as an acrosome.				
NEGATIVE_REGULATION_OF_CELLULAR_PROCESS [646]	Genes annotated by the GO term GO:0048523. Any process that stops, prevents or reduces the frequency, rate or extent of cellular processes, those that are carried out at the cellular level, but are not necessarily restricted to a single cell. For example, cell communication occurs among more than one cell, but occurs at the cellular level.	8		$1.15 e^{-4}$	$5.51 e^{-3}$
PID_HNF3APATHWAY [44]	FOXA1 transcription factor network	3		$1.39 e^{-4}$	$6.53 e^{-3}$
SYSTEM_DEVELOPMENT [861]	Genes annotated by the GO term GO:0048731. The process whose specific outcome is the progression of an organismal system over time, from its formation to the mature structure. A system is a regularly interacting or interdependent group of organs or tissues that work together to carry out a given biological process.	9		$1.54 e^{-4}$	$7 e^{-3}$
NEGATIVE_REGULATION_OF_BIOLOGICAL_PROCESS [677]	Genes annotated by the GO term GO:0048519. Any process that stops, prevents or reduces the frequency, rate or extent of a biological process. Biological processes are regulated by many means; examples include the control of gene expression, protein modification or interaction with a protein or substrate molecule.	8		$1.59 e^{-4}$	$7 e^{-3}$
REACTOME_LIPID_DIGESTION_MOBILIZATION_AND_TRANSPORT [46]	Genes involved in Lipid digestion, mobilization, and transport	3		$1.59 e^{-4}$	$7 e^{-3}$
REACTOME_MUSCLE_CONTRACTION [48]	Genes involved in Muscle contraction	3		$1.81 e^{-4}$	$7.79 e^{-3}$
POSITIVE_REGULATION_OF_EPITHELIAL_CELL_PROLIFERATION [10]	Genes annotated by the GO term GO:0050679. Any process that activates or increases the rate or extent of epithelial cell proliferation.	2		$2.26 e^{-4}$	$9.53 e^{-3}$
KEGG_ARGININE_AND_PROLINE_METABOLISM [54]	Arginine and proline metabolism	3		$2.57 e^{-4}$	$1.06 e^{-2}$
GLUCOSE_CATABOLIC_PROCESS [11]	Genes annotated by the GO term GO:0006007. The chemical reactions and pathways resulting in the breakdown of glucose, the aldohexose gluco-hexose.	2		$2.75 e^{-4}$	$1.12 e^{-2}$
ORGAN_DEVELOPMENT [571]	Genes annotated by the GO term GO:0048513. Development of a tissue or tissues that work together to perform a specific function or functions. Development pertains to the process	7		$3.31 e^{-4}$	$1.32 e^{-2}$

	whose specific outcome is the progression of a structure over time, from its formation to the mature structure. Organs are commonly observed as visibly distinct structures, but may also exist as loosely associated clusters of cells that work together to perform a specific function or functions.				
REACTOME_APOPTOSIS [148]	Genes involved in Apoptosis	4		$3.7\ e^{-4}$	$1.45\ e^{-2}$
BIOCARTA_AHSP_PATHWAY [13]	Hemoglobin's Chaperone	2		$3.89\ e^{-4}$	$1.47\ e^{-2}$
REACTOME_PURINE_SALVAGE [13]	Genes involved in Purine salvage	2		$3.89\ e^{-4}$	$1.47\ e^{-2}$
REGULATION_OF_MULTICELLULAR_ORGANISMAL_PROCESS [151]	Genes annotated by the GO term GO:0051239. Any process that modulates the frequency, rate or extent of an organismal process, the processes pertinent to the function of an organism above the cellular level; includes the integrated processes of tissues and organs.	4		$3.99\ e^{-4}$	$1.48\ e^{-2}$
PID_ERA_GENOMIC_PATHWAY [65]	Validated nuclear estrogen receptor alpha network	3		$4.44\ e^{-4}$	$1.58\ e^{-2}$
NEGATIVE_REGULATION_OF_CELL_PROLIFERATION [156]	Genes annotated by the GO term GO:0008285. Any process that stops, prevents or reduces the rate or extent of cell proliferation.	4		$4.51\ e^{-4}$	$1.58\ e^{-2}$
NEGATIVE_REGULATION_OF_HYDROLASE_ACTIVITY [14]	Genes annotated by the GO term GO:0051346. Any process that stops or reduces the rate of hydrolase activity, the catalysis of the hydrolysis of various bonds.	2		$4.53\ e^{-4}$	$1.58\ e^{-2}$
REACTOME_REGULATION_OF_COMPLEMENT_CASCADE [14]	Genes involved in Regulation of Complement cascade	2		$4.53\ e^{-4}$	$1.58\ e^{-2}$
KEGG_PURINE_METABOLISM [159]	Purine metabolism	4		$4.85\ e^{-4}$	$1.66\ e^{-2}$
NABA_MATRISOME [807]	Ensemble of genes encoding extracellular matrix and extracellular matrix-associated proteins	8		$5.12\ e^{-4}$	$1.72\ e^{-2}$
PID_THROMBIN_PAR4_PATHWAY [15]	PAR4-mediated thrombin signaling events	2		$5.22\ e^{-4}$	$1.73\ e^{-2}$
KEGG_PPAR_SIGNALING_PATHWAY [69]	PPAR signaling pathway	3		$5.29\ e^{-4}$	$1.73\ e^{-2}$
PID_CDC42_PATHWAY [70]	CDC42 signaling events	3		$5.52\ e^{-4}$	$1.75\ e^{-2}$
REACTOME_IMMUNOREGULATORY_INTERACTIONS_BETWEEN_A_LYMPHOID_AND_A_NON_LYMPHOID_CELL [70]	Genes involved in Immunoregulatory interactions between a Lymphoid and a non-Lymphoid cell	3		$5.52\ e^{-4}$	$1.75\ e^{-2}$
EPIDERMIS_DEVELOPMENT [71]	Genes annotated by the GO term GO:0008544. The process whose specific outcome is the progression of the epidermis over time, from its formation to the mature structure. The epidermis is the outer epithelial layer of a plant or animal, it may be a single layer that produces an extracellular material (e.g. the cuticle of arthropods) or a	3		$5.75\ e^{-4}$	$1.78\ e^{-2}$

	complex stratified squamous epithelium, as in the case of many vertebrate species.				
REACTOME_CHYLOMICRON_MEDIATED_LIPID_TR_TRANSPORT [16]	Genes involved in Chylomicron-mediated lipid transport	2		$5.96 e^{-4}$	$1.78 e^{-2}$
REACTOME_FORMATION_OF_ATP_BY_CHEMIOSMOSMOTIC_COUPLING [16]	Genes involved in Formation of ATP by chemiosmotic coupling	2		$5.96 e^{-4}$	$1.78 e^{-2}$
RIBONUCLEOTIDE_METABOLIC_PROCESS [16]	Genes annotated by the GO term GO:0009259. The chemical reactions and pathways involving a ribonucleotide, a compound consisting of ribonucleoside (a base linked to a ribose sugar) esterified with a phosphate moiety at either the 3' or 5'-hydroxyl group of its glycose moiety.	2		$5.96 e^{-4}$	$1.78 e^{-2}$
MULTICELLULAR_ORGANISMAL_DEVELOPMENT [1049]	Genes annotated by the GO term GO:0007275. The biological process whose specific outcome is the progression of an organism over time from an initial condition (e.g. a zygote or a young adult) to a later condition (e.g. a multicellular animal or an aged adult).	9		$6.45 e^{-4}$	$1.9 e^{-2}$
ORGANELLE_ORGANIZATION_AND_BIOGENESIS [473]	Genes annotated by the GO term GO:0006996. A process that is carried out at the cellular level which results in the formation, arrangement of constituent parts, or disassembly of any organelle within a cell.	6		$7.47 e^{-4}$	$2.18 e^{-2}$
REACTOME_RESPIRATORY_ELECTRON_TRANSPORPORT [79]	Genes involved in Respiratory electron transport	3		$7.86 e^{-4}$	$2.24 e^{-2}$
REACTOME_METABOLISM_OF_LIPIDS_AND_LIPOIPOPROTEINS [478]	Genes involved in Metabolism of lipids and lipoproteins	6		$7.89 e^{-4}$	$2.24 e^{-2}$
ECTODERM_DEVELOPMENT [80]	Genes annotated by the GO term GO:0007398. The process whose specific outcome is the progression of the ectoderm over time, from its formation to the mature structure. In animal embryos, the ectoderm is the outer germ layer of the embryo, formed during gastrulation.	3		$8.15 e^{-4}$	$2.28 e^{-2}$
REACTOME_SYNTHESIS_AND_INTERCONVERSIONION_OF_NUCLEOTIDE_DI_AND_TRIPHOSPHATES [19]	Genes involved in Synthesis and interconversion of nucleotide di- and triphosphates	2		$8.46 e^{-4}$	$2.34 e^{-2}$
KEGG_HYPERTROPHIC_CARDIOMYOPATHY_HCM [85]	Hypertrophic cardiomyopathy (HCM)	3		$9.72 e^{-4}$	$2.65 e^{-2}$
BIOCARTA_NDKDYNAMIN_PATHWAY [21]	Endocytotic role of NDK, Phosphins and Dynamin	2		$1.04 e^{-3}$	$2.79 e^{-2}$
REACTOME_RESPONSE_TO_ELEVATED_PLATELETLET_CYTOSOLIC_CA2_ [89]	Genes involved in Response to elevated platelet cytosolic Ca2+	3		$1.11 e^{-3}$	$2.95 e^{-2}$
REACTOME_METABOLISM_OF_AMINO_ACIDS_ANDAND_DERIVATIVES [200]	Genes involved in Metabolism of amino acids and derivatives	4		$1.14 e^{-3}$	$3 e^{-2}$
POSITIVE_REGULATION_OF_BIOLOGICAL_PROCROCESS [709]	Genes annotated by the GO term GO:0048518. Any process that activates or increases the frequency, rate or extent of a biological	7		$1.18 e^{-3}$	$3.06 e^{-2}$

	process. Biological processes are regulated by many means; examples include the control of gene expression, protein modification or interaction with a protein or substrate molecule.				
KEGG_DILATED_CARDIOMYOPATHY [92]	Dilated cardiomyopathy	3		$1.22\ e^{-3}$	$3.13\ e^{-2}$
CELLULAR_CARBOHYDRATE_CATABOLIC_PROCESS [23]	Genes annotated by the GO term GO:0044275. The chemical reactions and pathways resulting in the breakdown of carbohydrates, any of a group of organic compounds based of the general formula Cx(H2O)y, as carried out by individual cells.	2		$1.24\ e^{-3}$	$3.15\ e^{-2}$
CYTOSKELETON_ORGANIZATION_AND_BIOGENESIS [208]	Genes annotated by the GO term GO:0007010. A process that is carried out at the cellular level which results in the formation, arrangement of constituent parts, or disassembly of cytoskeletal structures.	4		$1.32\ e^{-3}$	$3.26\ e^{-2}$
REACTOME_PLATELET_ACTIVATION_SIGNALING_AND_AGGREGATION [208]	Genes involved in Platelet activation, signaling and aggregation	4		$1.32\ e^{-3}$	$3.26\ e^{-2}$
CARBOHYDRATE_CATABOLIC_PROCESS [24]	Genes annotated by the GO term GO:0016052. The chemical reactions and pathways resulting in the breakdown of carbohydrates, any of a group of organic compounds based of the general formula Cx(H2O)y.	2		$1.36\ e^{-3}$	$3.32\ e^{-2}$
POSITIVE_REGULATION_OF_CELL_DIFFERENTIATION [25]	Genes annotated by the GO term GO:0045597. Any process that activates or increases the frequency, rate or extent of cell differentiation.	2		$1.47\ e^{-3}$	$3.52\ e^{-2}$
REGULATION_OF_HEART_CONTRACTION [25]	Genes annotated by the GO term GO:0008016. Any process that modulates the frequency, rate or extent of heart contraction. Heart contraction is the process by which the heart decreases in volume in a characteristic way to propel blood through the body.	2		$1.47\ e^{-3}$	$3.52\ e^{-2}$
KEGG_PENTOSE_PHOSPHATE_PATHWAY [27]	Pentose phosphate pathway	2		$1.72\ e^{-3}$	$4.06\ e^{-2}$
GLUCOSE_METABOLIC_PROCESS [28]	Genes annotated by the GO term GO:0006006. The chemical reactions and pathways involving glucose, the aldohexose gluco-hexose. D-glucose is dextrorotatory and is sometimes known as dextrose; it is an important source of energy for living organisms and is found free as well as combined in homo- and hetero-oligosaccharides and polysaccharides.	2		$1.85\ e^{-3}$	$4.32\ e^{-2}$
PID_INTEGRIN2_PATHWAY [29]	Beta2 integrin cell	2			

	surface interactions			$1.98\ e^{-3}$	$4.59\ e^{-2}$

Gene/geneset overlap matrix

Entrez Gene Id	Gene Symbol	KEGG_RIBOSOME	REACTOME_3_UTR_MEDIATED_TRANSLATIONAL_REGULATION	REACTOME_TRANSLATION	REACTOME_PEPTIDE_CHAIN_ELONGATION	REACTOME_INFLUENZA_VIRAL_RNA_TRANSCRIPTION_AND_REPLICATION	REACTOME_NONSENSE_MEDIATED_DECAY_ENHANCED_BY_THE_EXON_JUNCTION_COMPLEX	REACTOME_SRP_DEPENDENT_COTRANSLATIONAL_PROTEIN_TARGETING_TO_MEMBRANE	REACTOME_INFLUENZA_LIFE_CYCLE	REACTOME_METABOLISM_OF_MRNA	REACTOME_METABOLISM_OF_RNA	REACTOME_METABOLISM_OF_PROTEINS	TRANSLATION	CELLULAR_BIOSYNTHETIC_PROCESS	BIOSYNTHETIC_PROCESS	MACROMOLECULE_BIOSYNTHETIC_PROCESS	REACTOME_FORMATION_OF_THE_TERNARY_COMPLEX_AND_SUBSEQUENTLY_THE_43S_COMPLEX	REACTOME_ACTIVATION_OF_THE_MRNA_UPON_BINDING_OF_THE_CAP_BINDING_COMPLEX_AND_EIFS_AND_SUBSEQUENT_BINDING_TO_43S	CELLULAR_PROTEIN_METABOLIC_PROCESS	CELLULAR_MACROMOLECULE_METABOLIC_PROCESS	PROTEIN_METABOLIC_PROCESS	KEGG_ALZHEIMERS_DISEASE	REACTOME_GLYCOLYSIS	REACTOME_GLUCONEOGENESIS	BIOCARTA_GLYCOLYSIS_PATHWAY	REACTOME_RESPIRATORY_ELECTRON_TRANSPORT_ATP_SYNTHESIS_BY_CHEMIOSMOTIC_COUPLING_AND_HEAT_PRODUCTION_BY_UNCOUPLING_PROTEINS_	STRIATED_MUSCLE_CONTRACTION_GO_0006941	BIOCARTA_MTA3_PATHWAY	REGULATION_OF_MUSCLE_CONTRACTION	KEGG_GLYCOLYSIS_GLUCONEOGENESIS	KEGG_PARKINSONS_DISEASE	KEGG_OXIDATIVE_PHOSPHORYLATION	REACTOME_TCA_CYCLE_AND_RESPIRATORY_ELECTRON_TRANSPORT	KEGG_COMPLEMENT_AND_COAGULATION_CASCADES	REACTOME_GLUCOSE_METABOLISM	REACTOME_METABOLISM_OF_NUCLEOTIDES	REACTOME_STRIATED_MUSCLE_CONTRACTION	PID_MYC_ACTIVPATHWAY	KEGG_CARDIAC_MUSCLE_CONTRACTION	REACTOME_LIPOPROTEIN_METABOLISM	SYSTEM_PROCESS	KEGG_FRUCTOSE_AND_MANNOSE_METABOLISM	KEGG_HUNTINGTONS_DISEASE	REACTOME_HEMOSTASIS	ANATOMICAL_STRUCTURE_DEVELOPMENT	NEGATIVE_REGULATION_OF_CELLULAR_PROCESS	PID_HNF3APATHWAY	SYSTEM_DEVELOPMENT	NEGATIVE_REGULATION_OF_BIOLOGICAL_PROCESS	REACTOME_LIPID_DIGESTION_MOBILIZATION_AND_TRANSPORT	REACTOME_MUSCLE_CONTRACTION	POSITIVE_REGULATION_OF_EPITHELIAL_CELL_PROLIFERATION	KEGG_ARGININE_AND_PROLINE_METABOLISM	GLUCOSE_CATABOLIC_PROCESS	ORGAN_DEVELOPMENT	REACTOME_APOPTOSIS	BIOCARTA_AHSP_PATHWAY	REACTOME_PURINE_SALVAGE	REGULATION_OF_MULTICELLULAR_ORGANISMAL_PROCESS	PID_ERA_GENOMIC_PATHWAY	NEGATIVE_REGULATION_OF_CELL_PROLIFERATION	NEGATIVE_REGULATION_OF_HYDROLASE_ACTIVITY	REACTOME_REGULATION_OF_COMPLEMENT_CASCADE	KEGG_PURINE_METABOLISM	NABA_MATRISOME	PID_THROMBIN_PAR4_PATHWAY	KEGG_PPAR_SIGNALING_PATHWAY	PID_CDC42_PATHWAY	REACTOME_IMMUNOREGULATORY_INTERACTIONS_BETWEEN_A_LYMPHOID_AND_A_NON_LYMPHOID_CELL	EPIDERMIS_DEVELOPMENT	REACTOME_CHYLOMICRON_MEDIATED_LIPID_TRANSPORT	REACTOME_FORMATION_OF_ATP_BY_CHEMIOSMOTIC_COUPLING	RIBONUCLEOTIDE_METABOLIC_PROCESS	MULTICELLULAR_ORGANISMAL_DEVELOPMENT	ORGANELLE_ORGANIZATION_AND_BIOGENESIS	REACTOME_RESPIRATORY_ELECTRON_TRANSPORT	REACTOME_METABOLISM_OF_LIPIDS_AND_LIPOPROTEINS	ECTODERM_DEVELOPMENT	REACTOME_SYNTHESIS_AND_INTERCONVERSION_OF_NUCLEOTIDE_DI_AND_TRIPHOSPHATES	KEGG_HYPERTROPHIC_CARDIOMYOPATHY_HCM	BIOCARTA_NDKDYNAMIN_PATHWAY	REACTOME_RESPONSE_TO_ELEVATED_PLATELET_CYTOSOLIC_CA2_	REACTOME_METABOLISM_OF_AMINO_ACIDS_AND_DERIVATIVES	POSITIVE_REGULATION_OF_BIOLOGICAL_PROCESS	KEGG_DILATED_CARDIOMYOPATHY	CELLULAR_CARBOHYDRATE_CATABOLIC_PROCESS	CYTOSKELETON_ORGANIZATION_AND_BIOGENESIS	[illegible]

6232	RPS27
6193	RPS5
6205	RPS11
6135	RPL11
6169	RPL38
11224	RPL35
6146	RPL22
6158	RPL28
6160	RPL31
6170	RPL39
6171	RPL41
6207	RPS13
6222	RPS18
6224	RPS20
6231	RPS26
6168	RPL37A
6139	RPL17
9349	RPL23
6136	RPL12
6165	RPL35A
1964	EIF1AX
1933	EEF1B2
5683	PSMA2
131118	DNAJC19
336	APOA2
10289	EIF1B
132	ADK
2593	GAMT
4728	NDUFS8
6687	SPG7
58526	MID1IP1
9530	BAG4
2597	GAPDH
1327	COX4I1
522	ATP5J
10476	ATP5H
4696	NDUFA3
487	ATP2A1
229	ALDOB
7167	TPI1

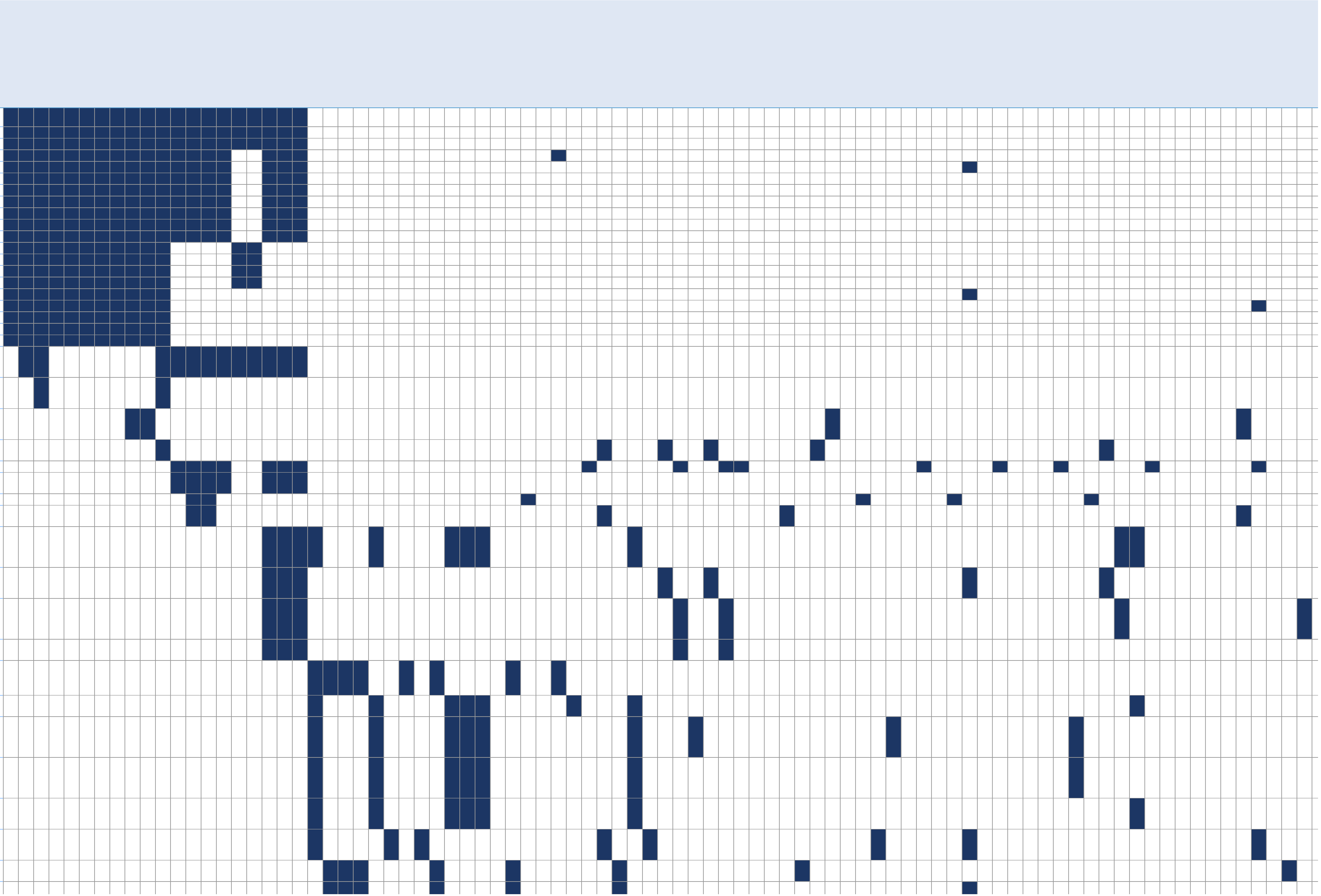

226	ALDOA
4633	MYL2
999	CDH1
7168	TPM1
2	A2M
2244	FGB
718	C3
3075	CFH
4831	NME2
4830	NME1
272	AMPD3
7139	TNNT2
338	APOB
1160	CKMT2
3043	HBB
2151	F2RL2
2173	FABP7
22985	ACIN1
5948	RBP2
686	BTD
58529	MYOZ1
6694	SPP2
8655	DYNLL1
8462	KLF11
259	AMBP
7494	XBP1
445	ASS1
3039	HBA1
2169	FABP2
567	B2M
9663	LPIN2
9524	TECR
25824	PRDX5
390502	SERPINA2
268	AMH
100507436	MICA
3240	HP
25949	SYF2
4619	MYH1
94239	H2AFV
689	BTF3
10181	RBM5
2879	GPX4

3029	HAGH
9388	LIPG
4677	NARS
10384	BTN3A3
10962	MLLT11
118980	SFXN2
2286	FKBP2
23744	ATP5EP1
3495	IGHD
352999	C6orf58
51506	UFC1
54749	EPDR1
55629	PNRC2
5816	PVALB
58476	TP53INP2
58505	OSTC
6253	RTN2
7140	TNNT3
79144	PPDPF
79803	HPS6
844	CASQ1

MSigDB database v5.0 updated March 2015
GSEA/MSigDB web site v5.0 released March 2015
